# Evolutionary History of the *Poecilia picta* Sex Chromosomes

**DOI:** 10.1093/gbe/evad030

**Published:** 2023-02-21

**Authors:** Lydia J M Fong, Iulia Darolti, David C H Metzger, Jake Morris, Yuying Lin, Benjamin A Sandkam, Judith E Mank

**Affiliations:** Department of Zoology and Biodiversity Research Centre, University of British Columbia, Canada; Department of Zoology and Biodiversity Research Centre, University of British Columbia, Canada; Department of Zoology and Biodiversity Research Centre, University of British Columbia, Canada; Department of Zoology, University of Cambridge, United Kingdom; Department of Zoology and Biodiversity Research Centre, University of British Columbia, Canada; Department of Neurobiology & Behavior, Cornell University; Department of Zoology and Biodiversity Research Centre, University of British Columbia, Canada

**Keywords:** sex chromosome, y chromosome, degeneration

## Abstract

The degree of divergence between the sex chromosomes is not always proportional to their age. In poeciliids, four closely related species all exhibit a male heterogametic sex chromosome system on the same linkage group, yet show a remarkable diversity in X and Y divergence. In *Poecilia reticulata* and *P. wingei*, the sex chromosomes remain homomorphic, yet *P. picta* and *P. parae* have a highly degraded Y chromosome. To test alternative theories about the origin of their sex chromosomes, we used a combination of pedigrees and RNA-seq data from *P. picta* families in conjunction with DNA-seq data collected from *P. reticulata*, *P. wingei*, *P. parae*, and *P. picta*. Phylogenetic clustering analysis of X and Y orthologs, identified through segregation patterns, and their orthologous sequences in closely related species demonstrates a similar time of origin for both the *P. picta* and *P. reticulata* sex chromosomes. We next used k-mer analysis to identify shared ancestral Y sequence across all four species, suggesting a single origin to the sex chromosome system in this group. Together, our results provide key insights into the origin and evolution of the poeciliid Y chromosome and illustrate that the rate of sex chromosome divergence is often highly heterogenous, even over relatively short evolutionary time frames.

**Significance**

The rate of sex chromosome divergence has been a topic of much recent interest. Specifically, it is not clear whether highly heteromorphic sex chromosomes can be associated with recent recombination arrest, or whether divergence is a more gradual process. The sex chromosomes in the poeciliids have become a promising model system for studying sex chromosome divergence rates, as several species share the same sex chromosome but show remarkably different rates of divergence. Our results suggest a single recent origin of recombination arrest in the immediate ancestor, followed by remarkable variation in the evolution of complete sex chromosome dosage compensation and Y chromosome degeneration.

## Introduction

The rate of sex chromosome divergence has been a topic of much recent interest (Bachtrog et al. 2011, 2014; Furman et al. 2020; Kratochvíl et al. 2021). It is clear that the degree of sex chromosome heteromorphism, or the magnitude of sequence difference between X and Y chromosomes, is not linear as a function of the time since they stopped recombining. In particular, old sex chromosomes may remain homomorphic (Mank and Ellegren 2007; Stöck et al. 2011; Kuhl et al. 2021), indicating that Y degeneration is neither inevitable nor the rate of degeneration predicted by age. It is less clear whether highly heteromorphic sex chromosomes can be associated with recent recombination arrest, or whether divergence, where it occurs, is a more gradual process.

The sex chromosomes in the poeciliids have become a promising model system for studying sex chromosome divergence rates. Four species, *Poecilia reticulata*, *P. picta*, *P. wingei*, and *P. parae* all exhibit a male heterogametic sex chromosome on the same linkage group, corresponding to Chromosome 12 in *P. reticulata* (Darolti et al. 2019; Sandkam et al. 2021), whereas two closely related outgroup species, *P. latipinna* and *Gambusia holbrooki*, do not exhibit sex chromosomes on this chromosome (Darolti et al. 2019). *P. wingei* and *P. reticulata* show low levels of X–Y divergence and accumulation of male-specific sequence in a small region (Wright et al. 2017; Darolti et al. 2019; Almeida et al. 2020; Fraser et al. 2020; Sigeman et al. 2021; Qiu et al. 2022). Phylogenetic analysis of X and Y sequence as well as shared Y sequence has shown recombination suppression in this region occurred in the ancestor of these two species (Morris et al. 2018; Almeida et al. 2020; Darolti et al. 2020). In contrast, *P. picta* and *P. parae* show high levels of X–Y divergence across nearly the entire chromosome and they both utilize complete X chromosome dosage compensation (Darolti et al. 2020; Metzger et al. 2021; Sandkam et al. 2021).

**Fig. 1.**
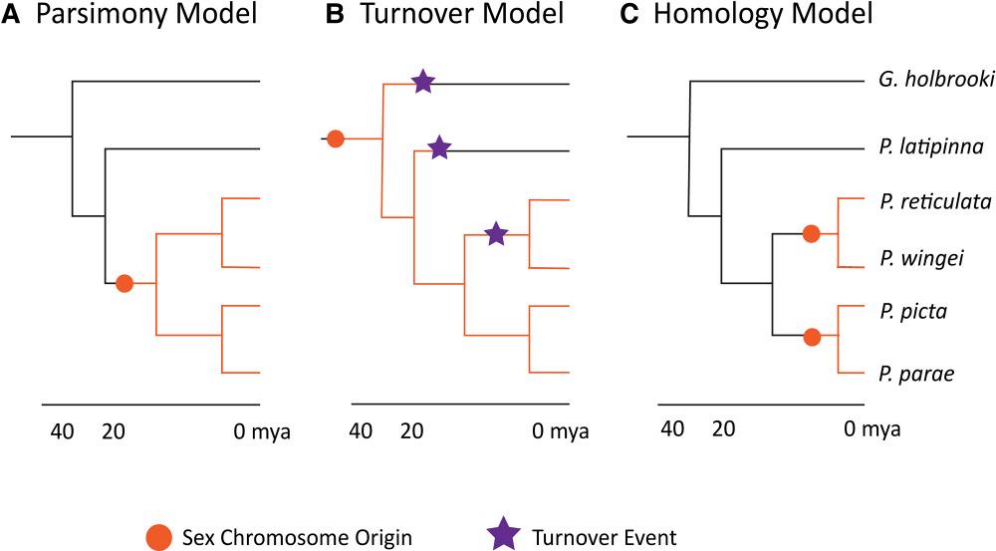
Three proposed models for the evolution of the poeciliid sex chromosomes. Black branches indicate lineages where the guppy Chromosome 12 is not implicated as the sex chromosome. Dots indicate the proposed origin of the sex chromosome. Stars indicate the turnover events. (*A*) The Parsimony Model, depicting a single-sex chromosome origin from the shared ancestor of *Poecilia reticulata*, *P. wingei*, *P. picta*, and *P. parae* ∼20 Mya. (*B*) The Turnover Model, depicting an ancient origin of the sex chromosome before the split with *Gambusia holbrooki* and *P. latipinna*. (*C*) The Homology Model, depicting two separate sex chromosome origins of *P. reticulata*, *P. wingei*, *P. picta*, and *P. parae*. One origin is in the ancestor of *P. reticulata* and *P. wingei* and one in the ancestor of *P. picta* and *P. parae* (Kirkpatrick et al. 2021).

Three alternative models have been proposed for the evolutionary history of the sex chromosomes in this clade (fig. 1). The Parsimony Model, based on the minimal number of evolutionary steps required, posits that the sex chromosome arose once in the common ancestor of *P. reticulata*, *P. wingei*, *P. picta*, and *P. parae*, roughly 20 million years ago (Mya) (Darolti et al. 2021; Metzger et al. 2021). This model implies differences in the rate of sex chromosome divergence after this single origin, with slow divergence in the immediate ancestor of *P. reticulata* and *P. wingei*, and rapid divergence in the *P. picta–P. parae* lineage. The rapid Y degeneration in the latter clade would theoretically be possible due to the recent and swift origin of complete X chromosome dosage compensation in the ancestor of *P. picta* and *P. parae* (Lenormand et al. 2020; Metzger et al. 2021).

In contrast to the Parsimony Model, others have proposed the Turnover Model based on the extensive degeneration of the *P. picta* Y chromosomes (Charlesworth, Bergero, et al. 2021). This model proposes that the sex chromosomes must be far older due to their extensive divergence, evolving before the common ancestor with *P. latipinna* and *G. holbrooki*. After this ancient origin, the model posits that the sex chromosomes have undergone turnover events in the ancestor of *P. reticulata* and *P. wingei*, where the Y was lost and the X began to diverge again into X and Y chromosomes. This model implies that turnovers also occurred in *P. latipinna* and *G. holbrooki*, where the X must have reverted to being an autosome.

The third model, which we refer to as the Homology Model, suggests that the sex chromosomes in *P. reticulata* and *P. wingei* evolved separately on the same chromosome as *P. picta* and *P. parae*, but after the divergence with *P. latipinna* (Kirkpatrick et al. 2021). This model suggests that the guppy chromosome 12 evolved twice as the sex chromosome, independently in each clade. Under this model, there would be no Y-specific sequence shared between the *P. picta–P. parae* and *P. reticulata–P.wingei* clades.

Which of these three models is correct has important implications for the potential rate of sex chromosome divergence, in particular, the potential for rapid evolution of complete sex chromosome dosage compensation and Y chromosome degeneration. Definitive evidence for one or the other models requires Y sequence in order to date recombination suppression in this lineage. To this end, we established four *P. picta* families and used RNA-Seq data from parents and offspring coupled with segregation patterns to determine X and Y coding sequences. Our results reveal similar levels of X–Y divergence on the *P. picta* sex chromosome compared with that of *P. reticulata* (Darolti et al. 2020), suggesting a similar timing for recombination suppression. We then used k-mer analysis on male and female genomes of *P. reticulata*, *P. picta*, *P. parae*, and *P. wingei* to determine the extent of k-mer sharing on the Y chromosomes of these species, another measure of the shared ancestry of the Y chromosome (Torres et al. 2018; Kabir et al. 2022). We found a large amount of shared male-specific sequence, indicating shared ancestry and a single evolutionary origin between our four species. Together, our results suggest a single recent origin of recombination arrest in the immediate ancestor of all four species.

## Results

### Identifying *X*-*Y* Coding Sequence in *P. picta*

The *de novo* transcriptome assembly from the pooled *P. picta* samples resulted in a total of 25,378 transcripts after filtering (supplementary table S1, Supplementary Material online). SEX-DETector, a probabilistic pedigree-based segregation method to identify X- and Y-linked genes from RNA-Seq data, was able to assign an inheritance pattern to 48.5% of the genes, of which 98.0% were autosomal (11,839 genes) and 2.0% were sex-linked (259 genes) (supplementary fig. S1, Supplementary Material online). Of the sex-linked genes, 209 were X0 genes, for which there was no corresponding Y-expressed gene, and 50 were XY (table 1). In total, 202 X0 and 35 XY genes mapped to the *P. picta* X chromosome, the seven X0 genes and 11 XY genes mapped to autosomes, and the remaining four XY genes were unplaced (table 1).

**Table 1:** Number of Sex-Linked Genes and False Positive Rate in *P. Picta*

Segregation Type	Inferred from SEX-DETector	Mapped to Chromosome 12	Mapped to Chromosome 12 Outside of the PAR	False Positives
XY	50	35	8	15
X0	209	202	198	7

Note.—False positives were genes that did not map to the reference *P. picta* chromosome 12.

We next removed sex-linked genes that mapped to the pseudoautosomal region (PAR) (supplementary fig. S2, Supplementary Material online, Materials and Methods). Although regions on the PAR-sex chromosome boundary may have experienced a recent loss of X–Y recombination suppression, it would not have occurred with sufficient time to allow for complete lineage sorting between X and Y orthologs, and therefore XY genes in this region are not phylogenetically informative. Of the sex-linked genes that mapped to the sex chromosome, 8 XY genes and 198 X0 genes were located outside the PAR boundary.

Because SEX-DETector may fail to identify highly diverged Y sequence, and in order to not bias our X–Y orthologs toward those with recent divergence, we also used expression characteristics to find additional Y genes. Using a male:female fold-change threshold of >2, we identified 27 genes with highly male-biased expression that we would expect for highly diverged Y genes where X-linked reads no longer map through homology (supplementary fig. S3, Supplementary Material online). Three of these genes were already called as XY genes by SEX-DETector. The 22 remaining male-biased genes mapped to the autosome of the female *P. picta* reference genome, one gene mapped to the PAR (TRINITY_DN52522_c0_g1_i6.p1; ∼30.7mb) on the X chromosome, and two genes did not map to the genome. When mapped to the male *P. picta* reference genome (Charlesworth, Graham, et al. 2021), all male-biased genes successfully mapped to the contigs, with one (TRINITY_DN52522_c0_g1_i6.p1) mapping to an X-contig and the remaining genes mapped to autosomal contigs (supplementary table S2, Supplementary Material online).

### Divergence Estimates Indicate Similar Timing of Recombination Suppression Across Species

**Fig. 2.**
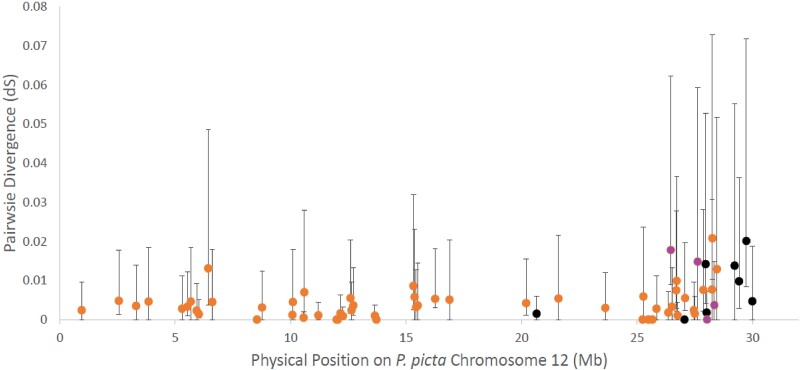
Synonymous substitution rate (d_S_) of *P. picta* and *P. reticulata* sex-linked genes mapped to the physical position of *P. picta* X Chromosome. *P. picta* XY genes are in black (*n* = 8), and *P. reticulata* XY genes (from Darolti et al. 2020) are in orange and purple (*n* = 57). Whiskers represent the standard error. Highlighted in purple are four genes that showed phylogenetic clustering consistent with ancestral recombination suppression in *P. reticulata* and *P. wingei* as identified in Darolti et al. (2020) (in order from left to right: *alad*, *LOC103474035*, *dnajc25*, *LOC103473940*). For clarity, X–Y genes inferred in the *P. picta* PAR, from 30MB, are not shown.

Synonymous substitution (d_S_) rate estimates between X and Y orthologs provide a relative measure of the time since recombination was halted between the orthologs. Genes within a region with similar d_S_ values suggest that recombination was halted around the same time in that region. We, therefore, estimated d_S_ for the eight XY genes that mapped to the sex chromosome outside the PAR (supplementary table S3, Supplementary Material online). The eight XY genes that mapped to the sex chromosome exhibit a d_S_ range 0.00–0.020 (average = 0.0082, fig. 2, supplementary table S3, Supplementary Material online).

In comparison, we mapped d_S_ values for *P. reticulata* identified with similar pedigree-based segregation methods and SEX-DETector (Darolti et al. 2020). If the sex chromosomes in *P. picta* diverged before the common ancestor with *Gambusia*, we would expect higher d_S_ values than we previously observed in *P. reticulata*, all demographic and selective factors otherwise being equal. If they diverged at a similar time, we would expect similar d_S_ values. We recovered very similar d_S_ patterns in *P. picta* compared with *P. reticulata* (fig. 2), with overlapping bootstrap values, consistent with a similar timing of recombination loss between the X and Y chromosomes in these two species.

### Phylogenetic Dating of Recombination Suppression Between the *P. picta* X and Y Chromosomes

**Fig. 3.**
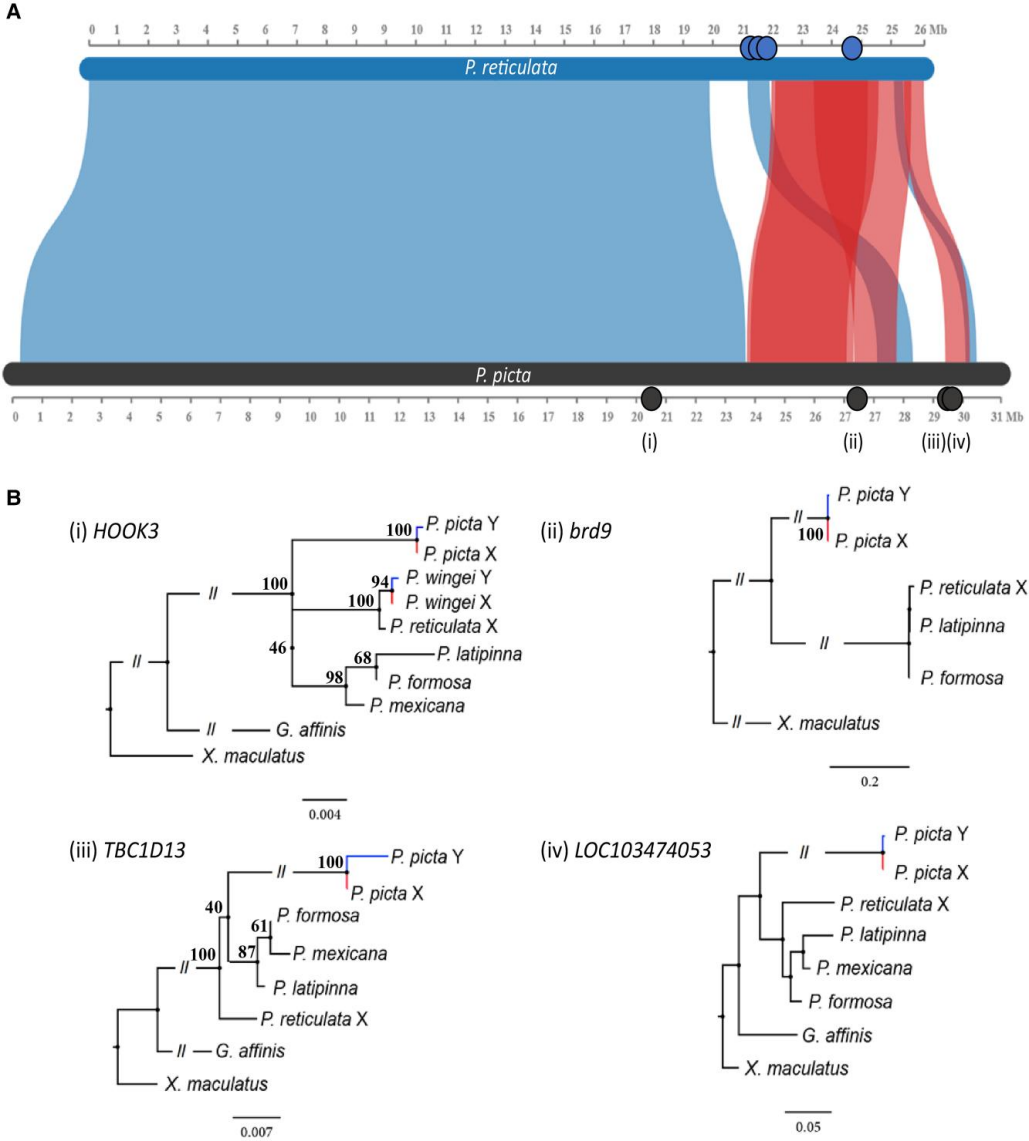
*P. reticulata* and *P. picta* sex chromosome; genes with orthologous sequences to the SEX-DETector X and Y sequences are highlighted. (*A*) Synteny plot between *P. reticulata* (top, blue) and *P. picta* (bottom, black) sex chromosomes. Red regions connecting the chromosomes represent inversions. Blue dots on *P. reticulata* chromosome are genes that showed phylogenetic clustering consistent with ancestral recombination suppression in *P. reticulata* and *P. wingei* (Darolti et al. 2020). Black dots (i, ii, iii, and iv) on the *P. picta* chromosome represent the location of X/Y sex-linked genes that had identified orthologs in at least one species. (*B*) Phylogenetic trees of *P. picta* genes and the corresponding orthologs that were found in the outgroups. Numbers by nodes represent bootstrap values from 100 permutations. All branches are bifurcating and apparent polytomies are due to short branch lengths.

The phylogenetic pattern of XY genes across multiple species can identify the branch in which recombination was halted (Wright et al. 2014). We identified orthologs in related species for the eight XY genes (supplementary table S4, Supplementary Material online), four of which remained after filtering for orthologs in related species (fig. 3). We previously identified four genes with phylogenetic patterns consistent with recombination suppression in the ancestor of *P. reticulata* and *P. wingei* (Darolti et al. 2020); however, none of these genes were present in our *P. picta* XY genes list, thereby making it impossible to use phylogenetic methods to date recombination suppression between these three species.

Although we cannot use our data to test for phylogenetic clustering of Y sequence across species in this group in the region of ancestral *P. reticulata-P. wingei* recombination suppression, we can use our XY genes to determine the timing of recombination suppression outside this relatively small region. If the *P. picta* sex chromosomes originated in the most recent common ancestor of *P. reticulata, P. wingei, P. picta*, and *P. parae* in the small region identified in Darolti et al. (2020) and then exhibited different rates of Y degeneration among the two daughter clades as recombination suppression spread independently (Parsimony Model), we would expect the *P. picta* Y ortholog to cluster most closely to the corresponding *P. picta* X chromosome. Alternatively, if X and Y divergence of the *P. picta* sex chromosome is more ancient, and the *P. reticulata* sex chromosomes arose from a turnover event (Turnover Model), we would expect the *P. picta* Y sequence to form an outgroup to the X sequences from all species within the group. Consistent with the Parsimony Model, maximum likelihood trees for all four genes show clustering of the *P. picta* X and Y sequences (fig. 3*b*).

### *P. picta* Y Chromosome Sequence

We constructed female and male k-mer profiles for *P. picta* (supplementary fig. S4*a*, Supplementary Material online). We expect any unique, non-repetitive Y-specific k-mers to show a coverage of roughly 15X given the haploid nature of the Y, and full diploid coverage at 30X. Indeed, we recovered a male k-mer peak around 10–15X, representing Y-unique k-mers. Repetitive regions enriched on the Y, though not necessarily Y-specific, would result in elevated k-mer coverage in males above the 30X average. Consistent with this, we see a slight increase in male k-mer coverage in the region of ≈45–80X coverage, representing Y-repetitive k-mers. This could be caused by an accumulation of repetitive elements on the Y chromosome (Mapleson et al. 2017; Morris et al. 2018).

Overall, our k-mer results suggest a modest amount of Y-unique and Y-repetitive sequence. To further explore this, we determined the total amount of sequence from putative Y contigs from the male *P. picta-*assembled genome (Charlesworth, Graham, et al. 2021) based on read depth comparisons between males and females. After removing contigs <10 kb, there were 1,271 contigs of which 96 were Y contigs, 168 were X contigs, and 1,007 were autosomal contigs (supplementary table S5, Supplementary Material online). This corresponds to 2.6 Mb of putative Y sequence (supplementary fig. S4*b*, Supplementary Material online, supplementary table S5, Supplementary Material online), compared with 29.6 Mb of putative X sequence and 700.1 Mb of putative autosomal/pseudo-autosomal sequence. This suggests that 0.35% of the male *P. picta* genome is Y-limited, which is broadly consistent with our k-mer-based profile analysis in this study as well as karyotype data (Nanda et al. 2022).

### Ancestral Y Chromosome Sequence

k-mer analysis has been a useful way to search for ancestral shared Y sequence among related species with a sex chromosome system of common origin (Torres et al. 2018; Kabir et al. 2022). To identify the ancestral Y chromosome sequence, we compared shared Y-mers (male-specific k-mers) across all four species, *P. reticulata*, *P. picta*, *P. parae*, and *P. wingei*. Although mitigated by looking for shared k-mers across multiple species, k-mer analysis can have a high Type I error rate (Almeida et al. 2020), so we calculated the number of shared female-specific k-mers as an estimate of the false positive rate.

**Fig. 4.**
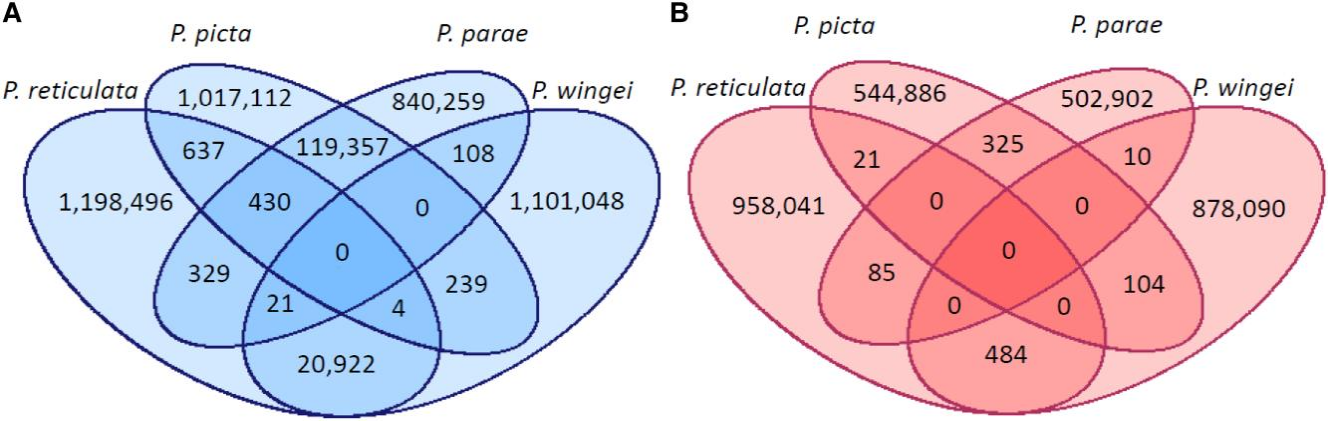
Male-specific (Y-mers) and female-specific k-mers of the four Poeciliid species of interest. (*A*) Y-mers of *P. reticulata*, *P. picta*, *P. parae*, and *P. wingei*. (*B*) Female-specific k-mers of *P. reticulata*, *P. picta*, *P. parae*, and *P. wingei,* representing the false positive rate.

If the sex chromosomes in *P. reticulata*, *P. picta*, *P. parae*, and *P. wingei* arose once in a common ancestor of all four species, we would expect more Y-mers shared across species than the female-specific k-mer false positive rate. If the sex chromosomes arose separately in *P. picta–P. parae* and *P. reticulata–P. wingei,* we would expect no more shared Y-mers across these species than the false positive rate. Overall, we found 130X more shared Y-mers compared with female-specific k-mers (142,047 vs. 1,029) (fig. 4).

Although most Y-mers were shared between *P. picta* and *P. parae* (119,357 vs. 325 female-specific k-mers) or between *P. reticulata* and *P. wingei* (20,922 vs. 484 female-specific k-mers), we also recovered a substantial number of Y-mers shared between at least one member of each clade (1,768 vs. 220 female-specific k-mers). This suggests a small region of recombination suppression ancestral to all four species followed by subsequent independent recombination suppression separately in the ancestor of *P. picta* and *P. parae* and in the ancestor of *P. reticulata* and *P. wingei* (Parsimony Model). Interestingly, we recovered no Y-mers shared across all four species. Although it is possible that synonymous substitutions in a shared Y-specific sex determining locus could eliminate Y-mers across all species, other Y-mer analyses across similar timescales and greater numbers of species have recovered the ancestral sex determining locus (Torres et al. 2018; Kabir et al. 2022). The lack of conserved Y-linked k-mers suggests that sex determination may be an X-linked dosage-sensitive mechanism.

## Discussion

It is clear that sex chromosome heteromorphism, or the magnitude of sequence difference between X and Y chromosomes, is not linear as a function of the time since they stopped recombining (Renner and Müller 2021). This disconnect between time and divergence was, until recently, largely thought to result from old sex chromosomes which retained homomorphy (Mank and Ellegren 2007; Stöck et al. 2011; Kuhl et al. 2021). However, recent work (Darolti et al. 2019; Prentout et al. 2021) has suggested that younger sex chromosomes may rapidly diverge, possibly aided in some cases by the evolution of dosage compensation (Lenormand et al. 2020). Poeciliids provide a unique model system to investigate the rate of divergence in sex chromosomes, as they contain species with both homomorphic and heteromorphic sex chromosomes on the same chromosome. However, establishing the evolutionary history of the sex chromosomes in this clade, particularly the timing, order, and number of recombination suppression events, is critical.

There are currently three models that have been proposed for the evolutionary history of the sex chromosomes in this clade (fig. 1). The Parsimony Model, based on the minimal number of evolutionary steps required, posits that the sex chromosomes arose in the ancestor to *P. reticulata*, *P. wingei*, *P. picta*, and *P. parae*, roughly 20 Mya (Darolti et al. 2021; Metzger et al. 2021). This single origin would have then been followed by differences in the rate of Y chromosome degeneration, slow in *P. reticulata* and *P. wingei*, and rapid in *P. picta* and *P. parae*. The rapid Y degeneration in the latter clade would theoretically be possible due to the recent and swift origin of complete X chromosome dosage compensation in the ancestor of *P. picta* and *P. parae* (Lenormand et al. 2020; Metzger et al. 2021).

There are two alternatives proposed to the Parsimony Model. The Turnover Model, based on the extensive degeneration of the *P. picta* Y chromosomes (Charlesworth, Bergero, et al. 2021), proposes that the sex chromosomes must be far older. Although Charlesworth, Bergero, et al. (2021) do not provide a specific estimate for the age of the sex chromosome, it implies it evolved before the common ancestor of at least *P. latipinna*, and also possibly *G. holbrooki*. This model posits turnover events in the ancestor of *P. reticulata* and *P. wingei*, where the Y was lost and the X began to diverge again into X and Y chromosomes, as well as in *P. latipinna* and possibly *G. holbrooki*, where presumably the X reverted to being an autosome. This model assumes that rapid degeneration of the Y is not possible despite the evolution of dosage compensation in the ancestor of *P. parae* and *P. picta* (Darolti et al. 2019; Lenormand et al. 2020; Metzger et al. 2021). The Homology Model suggests that the sex chromosomes in *P. reticulata* and *P. wingei* evolved separately on the same chromosome from those in *P. picta* and *P. parae*, but after the divergence with *P. latipinna* (Kirkpatrick et al. 2021).

Previous work (Darolti et al. 2021) has identified a Fast-X effect (Charlesworth et al. 1987) confined to *P. picta* and *P. parae*, consistent with either the Parsimony or Homology Models, however differentiating among all three requires X and Y sequence in species from both *P. picta–P. parae* and *P. reticulata–P. wingei* clades. We address that here, through both segregation analysis and pedigrees to determine X and Y coding sequence, as well as k-mer analysis, to determine shared Y sequence.

### Recombination Suppression on the *P. picta* sex Chromosome

We used M:F mapping coverage to delineate the PAR region. Because recombination persists in this region, there is no Y degeneration and M:F coverage values are statistically similar. Using this approach, we identified a clear PAR from 30 MB to the distal end of the chromosome (supplementary fig. S3, Supplementary Material online), and all X–Y genes in this region were removed from further analysis (and from fig. 1). Using SEX-DETector (Muyle et al. 2016) in conjunction with four family pedigrees, we identified eight XY sex-linked *P. picta* genes located outside of the predicted PAR. We were unable to find any additional Y-linked genes outside the PAR using gene expression data, suggestive that SEX-DETector was sufficient in calling XY genes. The low number of XY genes outside the PAR is consistent with widespread loss of Y chromosome coding content in *P. picta,* which has also been observed in *P. parae* (Darolti et al. 2019; Metzger et al. 2021). This gene loss is likely due to the evolution of X chromosome dosage compensation, which removes purifying selection on dosage-sensitive Y genes and has been predicted to lead to rapid degeneration of Y gene activity (Lenormand et al. 2020).

We used the rate of synonymous substitutions (d_S_) and phylogenetic clustering to determine the rate of divergence relative to what we previously observed in *P. reticulata* (Darolti et al. 2020). The Parsimony Model (Darolti et al. 2021) predicts that XY divergence will be similar in *P. reticulata* and *P. picta* in and around the vicinity of the region of ancestral recombination suppression. Outside of the ancestral non-recombining region, we expect *P. picta* X and Y orthologs to cluster most closely to each other. In contrast, if the Turnover Model (Charlesworth, Bergero, et al. 2021) is correct, XY divergence will be far greater in *P. picta*, and *P. picta* Y sequence will form an outgroup to X sequence from across the clade.

We found similar d_S_ values between *P. picta* and previously reported d_S_ values for *P. reticulata* (Darolti et al. 2020) (fig. 2), consistent with a similar time of recombination suppression. We also observe phylogenetic clustering of *P. picta* X and Y orthologs. This does not support the Turnover Model, which implies far greater XY divergence in *P. picta* compared with *P. reticulata*. Interestingly, the X–Y loci that are the most distant from the PAR boundary have lower point estimates for d_S_, although with overlapping confidence intervals, than X–Y genes nearer the boundary, suggesting the overall low X–Y divergence we observe is not a function of proximity to the PAR boundary.

We observed significant variation in X–Y d_S_ values, which ranged from 0.00 to 0.020 (supplementary table S3, Supplementary Material online). Younger sex chromosomes, and young strata within older sex chromosomes, can exhibit substantial heterogeneity in d_S_. For example, the threespine stickleback (*Gasterosteus aculeatus*) and white campion (*Silene latifolia*) X and Y chromosomes originated ∼30 and <10 Ma, respectively. Work on these systems indicates that recombination suppression evolves heterogeneously (Chibalina and Filatov 2011; Muyle et al. 2012; Bergero et al. 2013; Natri et al. 2013; Qiu et al. 2013; Roesti et al. 2013), suggesting that although selection to suppress recombination between the X and Y chromosomes occurs over large regions consistent with strata, within those regions, the effects are variable and genetic exchange between the sex chromosomes persists in some places. This is exactly what we observe in *Poecilia* (fig. 2). Importantly, although we observe high heterogeneity, all the estimates were very low (all ≤0.02), which is not compatible with the greater divergence expected under the Turnover Model.

It is also worth noting that we lack any informative XY sequence over the large span from 0 ≈ 20 Mb. This could have resulted from a large-scale deletion on the *P. picta* Y chromosome, as has also been observed in stickleback (Leder et al. 2010), although it is also possible that this represents an older, fully degenerate portion of the Y chromosome. However, the small size of the Y chromosome, estimated here via sequence and M:F coverage to be just 2.6 Mb, compared with 29.6 Mb of putative X-specific sequence (supplementary fig. S4*b*, Supplementary Material online, supplementary table S5, Supplementary Material online), the low overall d_S_ values we observe, the failure to detect highly diverged Y sequence from expression data, as well as recent cytogenetic evidence (Nanda et al. 2022), suggests large-scale deletions are more plausible.

### Ancestral Sequence of the Three Poecilid Species

We previously observed a small region of Y degradation in *P. reticulata* and *P. wingei* (Wright et al. 2017; Darolti et al. 2019; Almeida et al. 2020). Although some previous work has failed to detect X–Y divergence based on M:F coverage analysis in this region in *P. reticulata* (Bergero et al. 2019; Charlesworth et al. 2020; Kirkpatrick et al. 2021), studies that have used similar genomic methods all recovered patterns consistent with this region (Wright et al. 2017; Darolti et al. 2019; Almeida et al. 2020; Fraser et al. 2020; Sigeman et al. 2021; Darolti et al. 2022), as have other studies using male-specific sequence (Morris et al. 2018; Almeida et al. 2020; Darolti et al. 2020).

Within this region, four genes exhibit phylogenetic clustering consistent with recombination suppression in the ancestor of *P. reticulata* and *P. wingei* (Darolti et al. 2020); however, none of these genes were present in our *P. picta* XY gene list, making it impossible to use phylogenetic methods to date recombination suppression between these three species. In order to differentiate the Parsimony (Darolti et al. 2021) from the Homology (Kirkpatrick et al. 2021) models, we, therefore, used k-mer analysis framework, which has previously been shown to be a useful way to search for shared ancestral Y sequence among related species with a sex chromosome system of common origin (Torres et al. 2018; Kabir et al. 2022). If the sex chromosomes in *P. reticulata*, *P. picta*, *P. parae*, and *P. wingei* arose once in a common ancestor of all four species, we would expect more Y-mers shared across species than the female-specific k-mer false positive rate. If the sex chromosomes arose separately in *P. picta–P. parae* and *P. reticulata–P. wingei,* we would expect no more shared Y-mers across these species than the false positive rate.

Consistent with the Parsimony Model, we recovered a substantial number of Y-mers shared between at least one member of each clade (fig. 4). This suggests a small region of recombination suppression ancestral to all four species followed by subsequent independent recombination suppression separately in the ancestor of *P. picta* and *P. parae* and in the ancestor of *P. reticulata* and *P. wingei*, as the most shared Y-mers were found between the two sister species.

Y-mer analysis can have a high false positive rate (Almeida et al. 2020); however, this is estimable through the female-specific k-mer number. In theory, because there is no female-specific part of genome with X–Y sex chromosomes, the female-specific number of k-mers is a fair estimate of the false positive rate. In all comparisons, our estimated Y-mers are orders of magnitude larger than the false positive estimates through female-specific k-mers, indicating that although some of our Y-mers may represent false positives, the overall large number of Y-mers represents shared ancestry.

It is also important to note that the evolution of complete dosage compensation is expected to result in rapid Y chromosome degeneration, as selection to maintain the activity of dosage-sensitive genes is eliminated (Lenormand et al. 2020; Lenormand and Roze 2022). Indeed, neo-sex chromosomes and turnover of Y and W chromosomes in systems with existing dosage compensation all experience rapid degeneration (Bachtrog et al. 2008; Dai et al. 2022). Therefore, we would expect that the evolution of complete dosage compensation in *P. picta* and *P. parae* (Darolti et al. 2019; Metzger et al. 2021) would result in the rapid degeneration of Y chromosome coding content in these species. Incidentally, we would expect that this would further reduce shared Y-mers, making it all the more remarkable that our shared Y-mer numbers exceed the false positive rate by an order of magnitude.

Interestingly, although we recovered Y-mers spanning each sub-clade, we recovered no Y-mers shared across all four species. It is theoretically possible that extensive synonymous substitutions in an ancestral Y-linked sex-determining locus would preclude the discovery of Y-mers across all four species. However, Y-mer analyses across similar timescales and greater numbers of species have recovered ancestral sex-determining loci (Torres et al. 2018; Kabir et al. 2022). Moreover, the low overall X–Y divergence we observe here in *P. picta* and previously in *P. reticulata* and *P. wingei* (Darolti et al. 2020) suggests that this is unlikely. Instead, the lack of conserved Y-linked k-mers suggests that sex determination may be an X-linked dosage-sensitive mechanism as observed in birds (Ioannidis et al. 2021), *Drosophila melanogaster*, *Caenorhabditis elegans* (Hodgkin 1990), and many other species.

## Conclusion

Poeciliids are model systems to study the rate of divergence in sex chromosomes, where closely related species have different degrees of degeneration of their Y chromosome. We use pedigree-based segregation and k-mer analysis to identify XY orthologs and male-specific sequence to critically test alternative models of Poeciliid sex chromosome evolution. Overall, we detect similar divergence between XY orthologs in *P. picta* compared with recent similar estimates in *P. reticulata* and *P. wingei*, and phylogenetic clustering patterns consistent with a single recent origin (Parsimony Model) of this sex chromosome system.

## Materials and Methods

### Families and Samples

To determine sex-linked inheritance patterns of *P. picta*, we established four breeding families of *P. picta* by crossing a virgin female with a male. All families included five male and five female siblings, as this is the minimum number of offspring per family required to reliably identify sex-linked genes using SEX-DETector (Muyle et al. 2016). We collected somatic tissue from the posterior region of the fish behind the anal fin from each individual which we preserved in RNAlater, and RNA was extracted with RNeasy Kit (Qiagen), following the manufacturer's instructions. Libraries were prepared by Genome Quebec and sequenced using Illumina NovaSeq 6000 S4 with 100-bp paired-end reads (data availability in supplementary table S6, Supplementary Material online). We followed Darolti et al. (2020) for quality assessment and preprocessing. Sample quality was assessed using FastQC v.0.10.1 (http://www.bioinformatics.babraham.ac.uk/projects/fastqc/, last accessed May 12, 2021) followed by adaptor removal and trimming using Trimmomatic v.0.36 (Lohse et al. 2012). We trimmed regions with average Phred score <15 in sliding windows of four bases, reads with Phred score <3 for leading and trailing bases, as well as paired-end reads with either read pair shorter than 50 bp. Following trimming, we had an average of 82 million reads per sample (supplementary table S7, Supplementary Material online).

### Constructing Transcriptome Assemblies

To estimate gene expression and to recover X and Y coding sequence, we constructed a transcriptome using Trinity v2.9.1 (Grabherr et al. 2011) with default parameters, combining reads from all samples. The transcriptome was then filtered to remove redundancy, noncoding RNA, and transcripts without an open reading frame. We used the built-in Trinity align_and_estimate_abundance.pl script that maps reads to the de novo transcriptome using Bowtie2 (Langmead et al. 2009) to remove unpaired and discordant alignments and used RSEM v.1.2.28 (Li and Dewey 2011) to estimate transcript abundance for each sample. The best isoform was selected for each gene based on the highest average expression. If multiple isoforms had the highest expression, we selected the longest isoform as the best isoform. We filtered for noncoding RNA by removing transcripts with a BLAST hit to the ncRNA sequence database for to *Oryzias latipes* (MEDAKA1) obtained from Ensembl 93 (Hunt et al. 2018). We used Transdecorder v.5.5.0 (http://transdecoder.github.io, last accessed July 5, 2021) with default parameters to identify coding regions within transcripts and removed those without open-reading frames and those with open-reading frames <150 bp.

### Inferring X-Y Sequence

We used the probabilistic software, SEX-DETector (Muyle et al. 2016), which analyzes parental and F1 genotypes to infer whether each contig exhibits an autosomal, XY (sex linked with both the X and Y copies present) or X0 (sex linked with the X copy present but no Y copy) segregation pattern. SEX-DETector requires gametologs to coassemble in one single transcript and, therefore, does not accurately identify Y-specific loci that either lack X-linked orthologs or which have extensive divergence. To reduce this problem, we further assembled contigs from Transdecoder with CAP3 (Huang and Madan 1999), and then all trimmed reads were mapped to the CAP3 assembly using BWA 0.6.1 (Li and Durbin 2009). We then merged and sorted all libraries of each family separately using SAMtools 1.3.1 (Li et al. 2009).

We genotyped all individuals at each locus using reads2snp v.2.0 (http://kimura.univ-montp2.fr/PopPhyl/, last accessed July 22, 2021), with a minimum number of three reads for calling a genotype (-*min* 3), a minimum base quality of 20 (-*bqt* 20), a minimum mapping quality of 10 (-*rqt* 10), the -*aeb* option for allowing alleles to have different expression levels. This is important for sex chromosome analyses in the presence of dosage compensation as the Y copy exhibits substantially lower expression levels compared with the X copy (Darolti et al. 2020), and the paraclean option was disabled (-*par* 0) to avoid the removal of paralogous positions since X and Y copies can resemble paralogs. This allows for SEX-DETector to infer the segregation type for each transcript within each of our four families, using a minimum posterior segregation type probability of 0.8. After genes were identified as autosomal, XY, or X0, we pooled sex-linked genes across replicate families for all subsequent analyses. If the sex-linked (XY or X0) gene was found in more than one family, we selected the X and Y sequence pairs that contained the highest number of SNP differences as the representative sex-linked sequences for the species.

### Additional Y Coding Sequence Detection

In addition to using SEX-DETector to infer sex-linked genes, we also looked at expression characteristics to determine whether there are any highly diverged Y genes. From the full transcript assembly, we counted gene expression using salmon v.1.4.0 (Patro et al. 2017) and normalized counts using edgeR (Robinson et al. 2010). We calculated the median male: female log_2_ fold change and removed genes that had median counts < 2 RPKM (but included genes that were sex-limited in expression, i.e., 0 RPKM for one sex).

### Assigning Chromosomal Position

We determined positional information *of P. picta* sex-linked gene sequences inferred from SEX-DETector with BLASTN and an e-value cutoff of 1^e−10^, using the female *P. picta* reference genome (supplementary table S6, Supplementary Material online). We took the top BLAST hit based on the highest BLAST bit score for genes with multiple hits and removed any genes that were assigned as sex-linked by SEX-DETector but did not map to *P. picta* chromosome 12, as these represent either false positives or unassembled scaffolds in the genome assembly (table 1).

We next delineated the PAR of the *P. picta* sex chromosomes by aligning and mapping *P. picta* paired-end DNA-seq of three males and three females from Darolti et al. (2019) to the female *P. picta* reference genome using BWA 0.6.1 (Li and Durbin 2009). We sorted aligned reads using SAMtools 1.3.1. (Li et al. 2009). We used soap.coverage v2.7.7 (https://bio.tools/soap) to extract coverage of every individual and extracted uniquely mapped reads using grep “XT:A:U.” We calculated the average coverage for males and females separately and used these averages to calculate the male:female (M:F) coverage (log_2_ average male coverage/log_2_ average female coverage) for each scaffold. PARs retain X–Y recombination and, therefore, exhibit equal coverage between males and females (autosomal-like coverage). Thus, we used the 95% autosomal confidence interval to delineate the *P. picta* PAR. We used only those sex-linked genes that mapped outside the PAR region of chromosome 12 for all downstream analyses (table 1, supplementary fig. S2, Supplementary Material online).

Next, we identified the synteny between the sex chromosome of the female *P. reticulata* and *P. picta* reference genome, following the MCScanX pipeline (Wang et al. 2012), manually correcting the inversion on chromosome 12 of the reference genome which is absent from other sequenced populations (Künstner et al. 2016; Darolti et al. 2019; Almeida et al. 2020; Fraser et al. 2020) for downstream analyses. We used the Synvisio Web Browser (Bandi and Gutwin 2020) to plot the synteny between *P. reticulata* and *P. picta* chromosome 12.

### Phylogenetic Analysis

We calculated pairwise synonymous substitution estimates (d_S_) between *P. picta* X and Y sequences using the yn00 program in PAML v.4.9 (Yang 2007) following the Yang and Nielsen method (Yang and Nielsen 2000). In order to phylogenetically date recombination suppression between the *P. picta* X–Y gametologs, we obtained transcripts from *P. reticulata*, *O. latipes*, *Xiphophorus maculatus*, *G. affinis*, *P. latipinna*, *P. formosa*, and *P. mexicana* from Ensembl 93 and identified the longest transcript for each gene. We determined orthology across all these species using a reciprocal BLASTN with an e-value cutoff of 1^e−10^ and a minimum percentage identity of 30%. We then used BLASTX to identify open-reading frames in each orthologous group. We included sequences from Darolti et al. (2019) for *P. reticulata* and *P. wingei* in the BLASTX analysis in order to identify any X or Y orthology with *P. picta*. We aligned sequences with PRANK v.170427 (Löytynoja and Goldman 2008), removing gaps from alignments and only retained orthologs >300 bp after gap removal. We used RAxML v.8.1.20 (Stamatakis 2014) to generate maximum likelihood phylogenetic trees, using the rapid bootstrap algorithm with the GTRGAMMA model and 100 bootstraps, and visualized trees with FigTree v.1.4.4 (http://tree.bio.ed.ac.uk/software/figtree/, last accessed October 10, 2021).

### Characterizing the *P. picta* Y Chromosome

We compared male and female k-mer profiles to determine the relative proportion of sequence that is unique to the Y chromosome as well as Y enrichment of repetitive elements (Morris et al. 2018). We used Jellyfish v.2.3.3 (Marçais and Kingsford 2011) to identify the k-mer composition of three male and three female *P. picta* (data from [Darolti et al. 2019]). To do this, we pooled paired-end DNA-seq reads by sex, then used the count command, counting canonically (-*C*) with 31-mers (-*m* 31) and a hash with 100 million elements (-*s* 100 M). The k-mers and their respective counts were outputted using the dump command and the k-mer composition was produced using the histo command (histogram output). The frequency of each k-mer (counts) was normalized to a targeted 30× coverage for each set of pooled individuals by the total base pairs remaining after trimming. k-mer profiles were then plotted in R (R Core Team 2021).

We also used read depth between males and females to identify Y contigs in the assembled male *P. picta* genome (Charlesworth, Graham, et al. 2021). We expect M:F read depth ≥ 1 for Y contigs and scaffolds, where female coverage can range from 0 in the case of no sequence homology between X and Y to a fraction of male coverage for those contigs that retain some similarity. We, therefore, used paired-end DNA-seq data from three male and three female *P. picta* samples (Darolti et al. 2019) and mapped it to contigs from the male *P. picta* genome (Charlesworth, Graham, et al. 2021) using BWA 0.6.1 (Li and Durbin 2009). The mapped reads were summarized using SAMtools 1.3.1. idxstats (Li et al. 2009). We removed contigs <10 kb to reduce noise (supplementary fig. S5, Supplementary Material online) and normalized individual reads by dividing contig read lengths and the total number of reads per individual, with median read depth calculated separately for females and males. We then bootstrapped the autosomal contigs 1,000 times to determine the 95% confidence intervals for M:F read depth (CI; lower = 0.9576; upper = 1.0876).

### Identifying Ancestral Y Sequence

k-mer analysis has been a useful way to search for shared ancestral Y sequence among related species with a sex chromosome system of common origin (Torres et al. 2018; Kabir et al. 2022). We, therefore, used DNAseq reads from three males and three females of *P. picta*, *P. wingei* (Darolti et al. 2019), *P. parae* (Sandkam et al. 2021), and *P. reticulata* for k-mer analysis (supplementary tables S6 and S8, Supplementary Material online). We used the HAWK v. 1.7.0 pipeline (Rahman et al. 2018) to count and identify male-specific k-mers (Y-mers) for each species using counts from the outputted Bonferroni-corrected file. We identified shared Y-mers across each species comparison, using shared k-mers in females as the estimate of the rate of false positives.

## Supplementary Material

evad030_Supplementary_Data

## Data Availability

All trimmed data generated for *P. picta* RNA-seq analysis can be accessed through the National Center for Biotechnology Information Sequencing Read Archive (https://www.ncbi.nlm.nih.gov/sra) under the Project ID “PRJNA856299'. All trimmed data generated for *P. reticulata* k-mer analysis can be accessed from the same website under the Project ID “PRJNA858015.” All data concerning the *P. picta* reference genome can be accessed from the same website under Project ID “PRJNA862953.” All other data used in this project were collected in other studies and can be accessed by their respective Project ID (see supplementary table S6, Supplementary Material online). All scripts and pipelines can be found at https://github.com/ljmfong/Poecilia_picta_Evol_Hist.
