# Supplementary material for: Evolutionary History of the Poecilia picta Sex Chromosomes
Source: Genome Biol Evol. 2023 Feb 21;15(3):evad030. doi: 10.1093/gbe/evad030 (PMC10003743; doi:10.1093/gbe/evad030)
Supplement: evad030_Supplementary_Data [file evad030_supplementary_data.zip › GBE_Revised_Evolutionary_History_of_the_Poecilia_picta_Sex_Chromosomes_Supplemental_Figures.pdf]

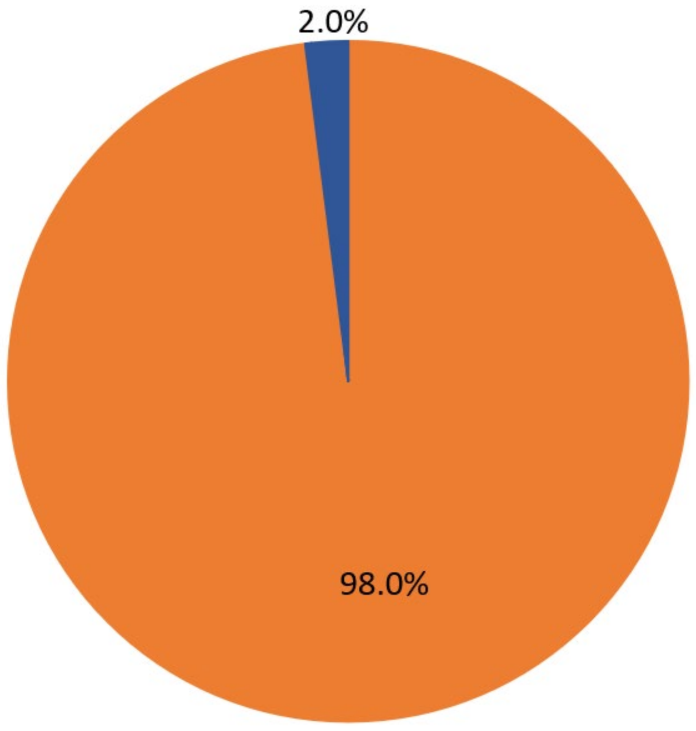

Supplemental Figure 1. Pie chart of the distribution of the SEX-DETector segregation types inferred from *P. picta* families. The orange area represents the autosomal genes (98.0%), and the dark blue area represents all inferred sex-linked genes (2.0%).

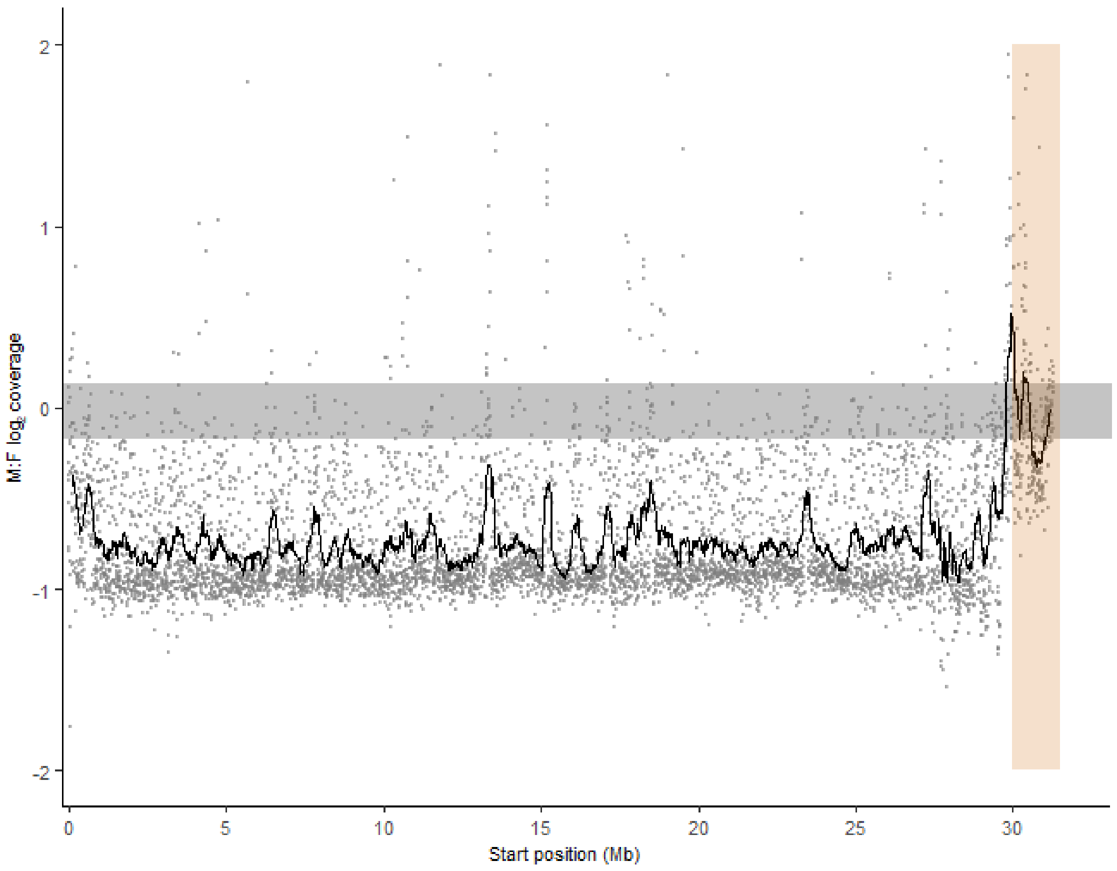

Supplemental Figure 2. Male: Female log2 coverage of *P. picta* chromosome 12 mapped to the *P. picta* female reference genome. The black line represents the moving average in 50kb windows. The grey horizontal bar is the 95% confidence interval computed from autosomal coverage. The orange region represents the estimated location of the PAR, ~30 – 31Mb.

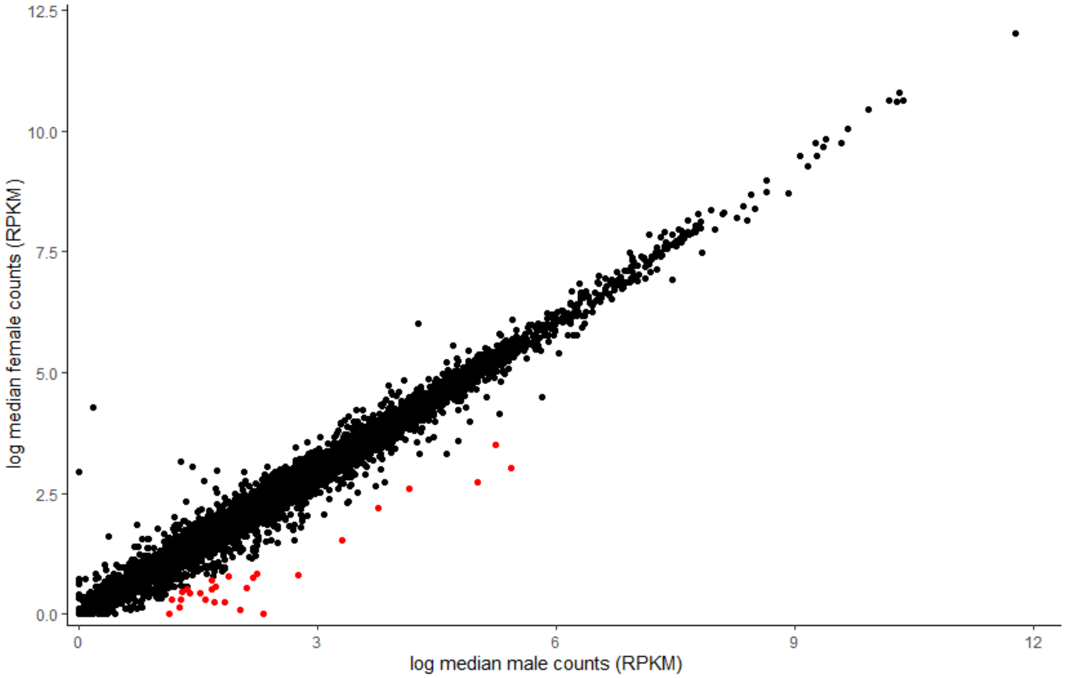

Supplemental Figure 3. The log median female and male gene counts in RPKM. All dots represent genes from the fully assembled *de novo* transcriptome. The red dots represent male-limited and male-biased gene expression (male fold-change >2). Genes that were lowly expressed (median counts < 2 RPKM) were removed from the analysis.

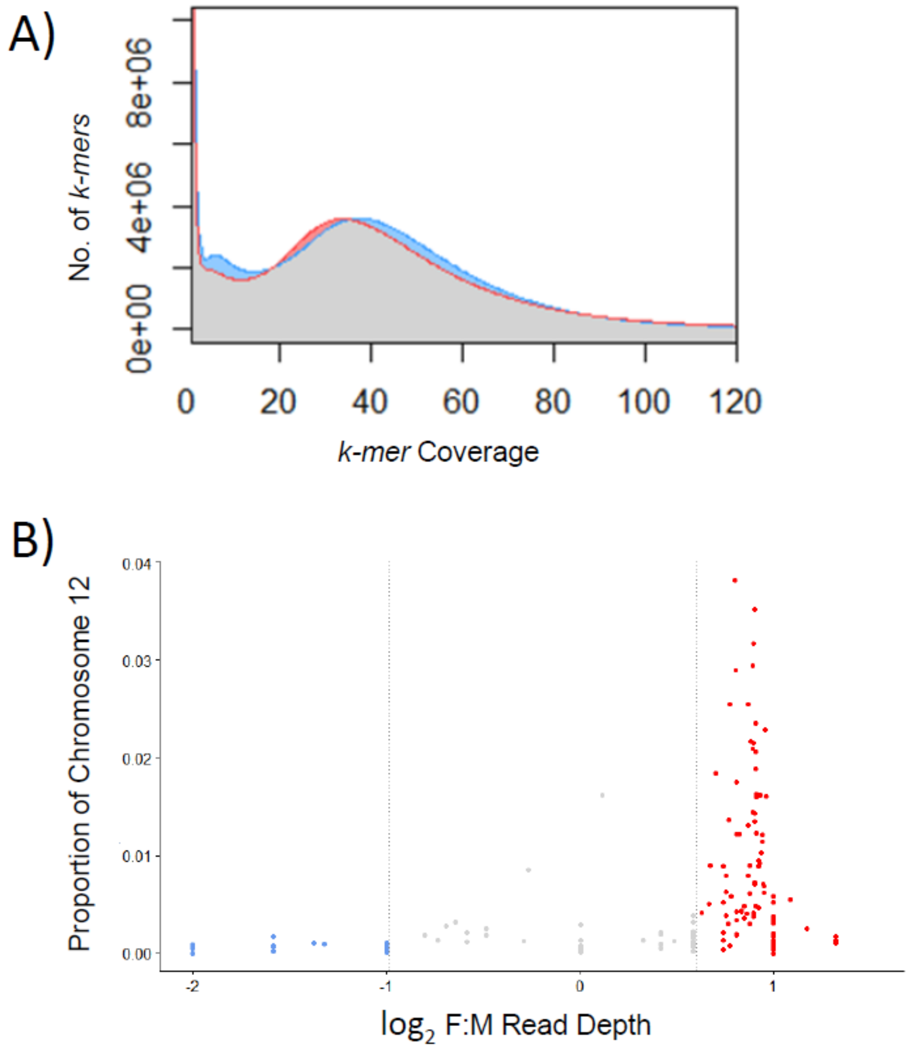

Supplemental Figure 4. The size of *P. picta* Y chromosome. A) The distribution of k-mers between the pooled male and female samples, normalized to 30x coverage. Shared k-mers are shaded in grey, male k-mers are shared in blue, and female k-mers are shared in red. B) The proportion of the sex chromosome (Chromosome 12) designated as Y contigs (blue; $\log_2$ F:M Read Depth ≤ -1), X contigs (red; $\log_2$ F:M Read Depth ≥ 0.6), and Autosomal contigs (grey; -1 < $\log_2$ F:M Read Depth < 0.6). The dotted line indicates the cut-off for the three contig types. Each dot represents a contig and its proportion was calculated as contig length/length of the X Chromosome (~31 Mb). Not all designated Y contigs from Supplemental Table 5 mapped to the X Chromosome and are not represented in this figure.

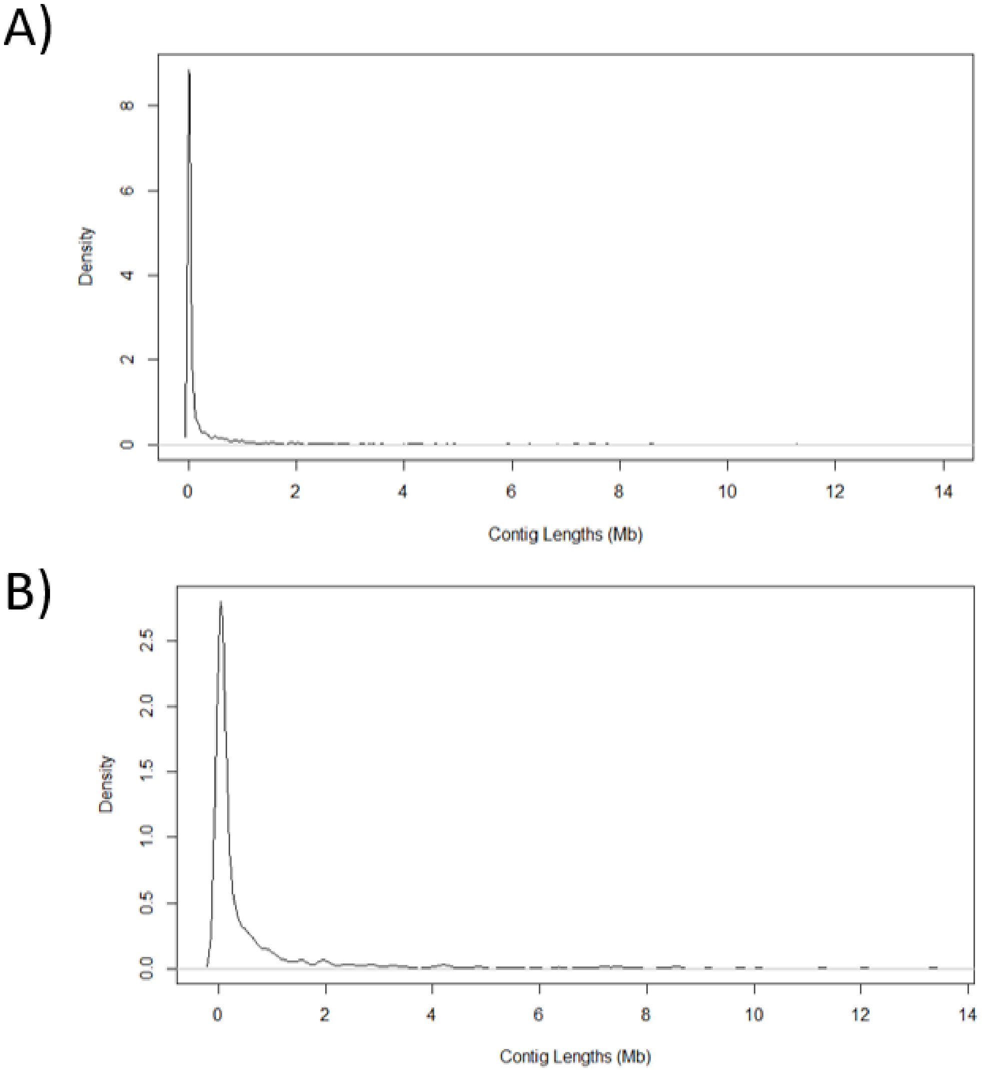

Supplemental Figure 5. Distribution of contig lengths (Mb) from Charlesworth, et al (2021). A) Distribution of the length of all contigs. B) Distribution of the length of contigs >10 kb. The distribution of contigs appears more normal.
