# Supplementary material for: Evolutionary History of the Poecilia picta Sex Chromosomes
Source: Genome Biol Evol. 2023 Feb 21;15(3):evad030. doi: 10.1093/gbe/evad030 (PMC10003743; doi:10.1093/gbe/evad030)
Supplement: evad030_Supplementary_Data [file evad030_supplementary_data.zip › GBE_Revision_Supplemental_Tables.pdf]

Supplemental Table 1. ***de novo*** **transcript assembly statistics of** ***P. picta*****.**

		Before filter	After best isoform filter	After ncRNA filter	After ORF filter	After CAP3 assembly
P. picta (n = 48)	No. of transcripts	583, 308	366, 609	366, 467	26, 433	25,378

Supplemental Table 2. BLAST results of male-biased expressed genes. Male-biased genes are called from the full *de novo P. picta* transcriptome.

Male-biased gene	Female *P. picta* reference genome chromosome	Start position (bp)	Male *P. picta* reference genome contig	Contig type
TRINITY_DN41484_c0_g1_i1.p1	Chromosome 18	27567105	ENA|CAJOCC010000027|CAJOCC010000027.1	autosomal
TRINITY_DN1465_c6_g1_i4.p2	Chromosome 20	541951	ENA|CAJOCC010000357|CAJOCC010000357.1	autosomal
TRINITY_DN13328_c0_g2_i1.p1			ENA|CAJOCC010000578|CAJOCC010000578.1	autosomal
TRINITY_DN17761_c0_g1_i2.p1	Chromosome 18	3307305	ENA|CAJOCC010000666|CAJOCC010000666.1	autosomal
TRINITY_DN5513_c1_g2_i1.p1	Chromosome 8	3947236	ENA|CAJOCC010000817|CAJOCC010000817.1	autosomal
TRINITY_DN5142_c0_g1_i19.p1	Chromosome 1	8581303	ENA|CAJOCC010001157|CAJOCC010001157.1	< 10kb
TRINITY_DN4515_c0_g4_i1.p1	Chromosome 9	10917910	ENA|CAJOCC010001392|CAJOCC010001392.1	autosomal
TRINITY_DN23869_c0_g1_i3.p1			ENA|CAJOCC010001652|CAJOCC010001652.1	autosomal
TRINITY_DN7183_c0_g1_i1.p1	Chromosome 9	32734400	ENA|CAJOCC010001652|CAJOCC010001652.1	autosomal
TRINITY_DN49157_c0_g1_i6.p1	Chromosome 3	16976201	ENA|CAJOCC010001664|CAJOCC010001664.1	autosomal
TRINITY_DN1349_c1_g1_i2.p1	Unplaced Scaffold	20481	ENA|CAJOCC010001666|CAJOCC010001666.1	autosomal
TRINITY_DN51220_c0_g1_i2.p2	Chromosome 18	28062374	ENA|CAJOCC010001672|CAJOCC010001672.1	autosomal
TRINITY_DN660_c1_g1_i13.p1	Chromosome 2	22433162	ENA|CAJOCC010001691|CAJOCC010001691.1	x-contig
TRINITY_DN52522_c0_g1_i6.p1	Chromosome 12	30733568	ENA|CAJOCC010001823|CAJOCC010001823.1	autosomal
TRINITY_DN3103_c0_g1_i21.p1	Chromosome 2	28880973	ENA|CAJOCC010001882|CAJOCC010001882.1	autosomal
TRINITY_DN7235_c0_g1_i21.p1	Chromosome 1	8583526	ENA|CAJOCC010001882|CAJOCC010001882.1	autosomal
TRINITY_DN2805_c0_g2_i3.p1	Chromosome 13	5167626	ENA|CAJOCC010001891|CAJOCC010001891.1	autosomal
TRINITY_DN5650_c0_g1_i30.p1	Chromosome 9	10919138	ENA|CAJOCC010001923|CAJOCC010001923.1	autosomal
TRINITY_DN956_c1_g2_i1.p1	Chromosome 8	15204936	ENA|CAJOCC010001929|CAJOCC010001929.1	autosomal
TRINITY_DN222206_c0_g1_i1.p1	Chromosome 17	13561468	ENA|CAJOCC010001929|CAJOCC010001929.1	autosomal
TRINITY_DN8129_c1_g1_i6.p1	Chromosome 4	14086107	ENA|CAJOCC010001930|CAJOCC010001930.1	autosomal
TRINITY_DN66859_c1_g1_i7.p1	Chromosome 9	17012287	ENA|CAJOCC010001934|CAJOCC010001934.1	autosomal
TRINITY_DN2528_c0_g1_i15.p2	Chromosome 7	9582977	ENA|CAJOCC010001936|CAJOCC010001936.1	autosomal
TRINITY_DN6218_c4_g1_i1.p1	Chromosome 15	9674990	ENA|CAJOCC010001946|CAJOCC010001946.1	autosomal

Supplemental Table 3. Pairwise divergence synonymous substitution rate of *P. picta* genes located on Chromosome 12 outside the PAR. Bootstrapping with 1000 replicates was used to determine standard error. Bolded cells indicate the genes with identified orthologs in at least one species.

Transcript	Position (bp)	dS ± SE
**TRINITY_DN580_c0_g1_i46.p1**	**20647794**	**0.0015 ± 0.0015**
TRINITY_DN1465_c6_g1_i4.p1	27052392	0. 0000 ± 0.0000
**TRINITY_DN22219_c0_g1_i4.p1**	**27965017**	**0.0142 ± 0.0101**
**TRINITY_DN241_c0_g1_i6.p1**	**28011155**	**0.0018 ± 0.0018**
**TRINITY_DN184_c2_g1_i9.p2**	**29223515**	**0.0138 ± 0.0138**
TRINITY_DN341_c1_g2_i5.p1	29412889	0.0098 ± 0.0069
TRINITY_DN258_c0_g1_i39.p1	29727262	0. 0201 ± 0.0116
TRINITY_DN5558_c0_g1_i6.p1	29989591	0. 0047 ± 0. 0047

Supplemental Table 4. *P. picta* genes, excluding PAR genes, and the orthologs in outgroups using Ensembl 93. Highlighted are the four genes that remained after filtering**.**

P. picta	*P. latipinna*	*P. formosa*	*P. mexicana*	*G. affinis*	*X. maculatus*	*P. reticulata*	*P. wingei*
TRINITY_DN5830_c0_g1_i46. p1	HOOK3	HOOK3	ENSPMEG00000008665	ENSGAFG00000006338	HOOK3	HOOK3	TRINITY_DN40933_c2_g1_i5. p1
TRINITY_DN1465_c6_g1_i4. p1	brd9	brd9	N/A	N/A	brd9	brd9	N/A
TRINITY_DN22219_c0_g1_i4. p1	ENSPLAG00000003413	ENSPFOG00000008933	ENSPMEG00000011326	N/A	ENSXMAG00000026616	ENSPREG00000012468	N/A
TRINITY_DN184_c2_g1_i9. p2	N/A	ENSPFOG00000004257	N/A	N/A	ENSXMAG00000019552	N/A	N/A
TRINITY_DN241_c0_g1_i6. p1	N/A	N/A	N/A	N/A	N/A	TRINITY_DN51150_c0_g1_i3. p1	Contig726
TRINITY_DN258_c0_g1_i39. p1	TBC1D13	TBC1D13	ENSPMEG00000001685	ENSGAFG00000008313	TBC1D13	TBC1D13	N/A
TRINITY_DN5558_c0_g1_i6. p1	ENSPLAG00000002572	ENSPFOG00000021874	ENSPMEG00000004726	ENSGAFG00000005023	ENSXMAG00000021293	ENSPREG00000006233 (LOC103474053)	N/A
TRINITY_DN341_c1_g2_i5. p1	N/A	N/A	N/A	N/A	N/A	N/A	N/A

Supplemental Table 5. Median read depth of mapped DNAseq to contigs >10kB. Contig type was designated as Y contigs, X contigs, and Autosomal contigs by using a log2 M:F Read Depth Threshold. Y contigs have log2 M:F Read Depth ⩾ 1, X contigs have log2 M:F Read Depth ⩽ -0.6, and Autosomal contigs are -0.6 < log2 M:F Read Depth < 1.

Contig	Contig type	Length (Kb)	Male Median Read Depth	Female Median Read Depth	M:F Read Depth	log2 M:F Read Depth
ENA|CAJOCC010000113|CAJOCC010000113.1	x_contig	51.738	1.00E-05	4.00E-05	2.50E-01	-2.0000
ENA|CAJOCC010000611|CAJOCC010000611.1	x_contig	23.552	1.00E-05	4.00E-05	2.50E-01	-2.0000
ENA|CAJOCC010000633|CAJOCC010000633.1	x_contig	25.775	1.00E-05	4.00E-05	2.50E-01	-2.0000
ENA|CAJOCC010000973|CAJOCC010000973.1	x_contig	33.516	1.00E-05	4.00E-05	2.50E-01	-2.0000
ENA|CAJOCC010001575|CAJOCC010001575.1	x_contig	23.321	1.00E-05	4.00E-05	2.50E-01	-2.0000
ENA|CAJOCC010000585|CAJOCC010000585.1	x_contig	47.441	1.00E-05	3.00E-05	3.33E-01	-1.5850
ENA|CAJOCC010001164|CAJOCC010001164.1	x_contig	54.384	1.00E-05	3.00E-05	3.33E-01	-1.5850
ENA|CAJOCC010001251|CAJOCC010001251.1	x_contig	4.152	1.00E-05	3.00E-05	3.33E-01	-1.5850
ENA|CAJOCC010001357|CAJOCC010001357.1	x_contig	39.785	1.00E-05	3.00E-05	3.33E-01	-1.5850
ENA|CAJOCC010001439|CAJOCC010001439.1	x_contig	10.746	1.00E-05	3.00E-05	3.33E-01	-1.5850
ENA|CAJOCC010001705|CAJOCC010001705.1	x_contig	33.049	1.00E-05	3.00E-05	3.33E-01	-1.5850
ENA|CAJOCC010000336|CAJOCC010000336.1	x_contig	42.187	2.00E-05	5.00E-05	4.00E-01	-1.3219
ENA|CAJOCC010001304|CAJOCC010001304.1	x_contig	54.324	2.00E-05	5.00E-05	4.00E-01	-1.3219
ENA|CAJOCC010001365|CAJOCC010001365.1	x_contig	32.745	2.00E-05	5.00E-05	4.00E-01	-1.3219
ENA|CAJOCC010001077|CAJOCC010001077.1	x_contig	78.041	4.00E-05	9.00E-05	4.44E-01	-1.1699
ENA|CAJOCC010000693|CAJOCC010000693.1	x_contig	171.945	8.00E-05	0.00017	4.71E-01	-1.0875
ENA|CAJOCC010000036|CAJOCC010000036.1	x_contig	19.215	1.00E-05	2.00E-05	5.00E-01	-1.0000
ENA|CAJOCC010000143|CAJOCC010000143.1	x_contig	15.16	1.00E-05	2.00E-05	5.00E-01	-1.0000
ENA|CAJOCC010000172|CAJOCC010000172.1	x_contig	4.067	1.00E-05	2.00E-05	5.00E-01	-1.0000
ENA|CAJOCC010000186|CAJOCC010000186.1	x_contig	77.69	2.00E-05	4.00E-05	5.00E-01	-1.0000
ENA|CAJOCC010000193|CAJOCC010000193.1	x_contig	61.996	2.00E-05	4.00E-05	5.00E-01	-1.0000
ENA|CAJOCC010000201|CAJOCC010000201.1	x_contig	51.09	1.00E-05	2.00E-05	5.00E-01	-1.0000
ENA|CAJOCC010000224|CAJOCC010000224.1	x_contig	118.946	7.00E-05	0.00014	5.00E-01	-1.0000
ENA|CAJOCC010000338|CAJOCC010000338.1	x_contig	15.241	1.00E-05	2.00E-05	5.00E-01	-1.0000
ENA|CAJOCC010000355|CAJOCC010000355.1	x_contig	26.329	1.00E-05	2.00E-05	5.00E-01	-1.0000
ENA|CAJOCC010000409|CAJOCC010000409.1	x_contig	20.87	2.00E-05	4.00E-05	5.00E-01	-1.0000

ENA|CAJOCC010000417|CAJOCC010000417.1	x_contig	163.901	1.00E-04	2.00E-04	5.00E-01	-1.0000
ENA|CAJOCC010000424|CAJOCC010000424.1	x_contig	5.591	1.00E-05	2.00E-05	5.00E-01	-1.0000
ENA|CAJOCC010000437|CAJOCC010000437.1	x_contig	19.722	1.00E-05	2.00E-05	5.00E-01	-1.0000
ENA|CAJOCC010000446|CAJOCC010000446.1	x_contig	1.244	1.00E-05	2.00E-05	5.00E-01	-1.0000
ENA|CAJOCC010000468|CAJOCC010000468.1	x_contig	28.124	1.00E-05	2.00E-05	5.00E-01	-1.0000
ENA|CAJOCC010000517|CAJOCC010000517.1	x_contig	12.417	1.00E-05	2.00E-05	5.00E-01	-1.0000
ENA|CAJOCC010000525|CAJOCC010000525.1	x_contig	112.376	6.00E-05	0.00012	5.00E-01	-1.0000
ENA|CAJOCC010000533|CAJOCC010000533.1	x_contig	34.567	1.00E-05	2.00E-05	5.00E-01	-1.0000
ENA|CAJOCC010000552|CAJOCC010000552.1	x_contig	21.685	1.00E-05	2.00E-05	5.00E-01	-1.0000
ENA|CAJOCC010000564|CAJOCC010000564.1	x_contig	11.777	1.00E-05	2.00E-05	5.00E-01	-1.0000
ENA|CAJOCC010000591|CAJOCC010000591.1	x_contig	33.466	2.00E-05	4.00E-05	5.00E-01	-1.0000
ENA|CAJOCC010000599|CAJOCC010000599.1	x_contig	57.163	3.00E-05	6.00E-05	5.00E-01	-1.0000
ENA|CAJOCC010000613|CAJOCC010000613.1	x_contig	95.92	5.00E-05	1.00E-04	5.00E-01	-1.0000
ENA|CAJOCC010000627|CAJOCC010000627.1	x_contig	18.496	1.00E-05	2.00E-05	5.00E-01	-1.0000
ENA|CAJOCC010000725|CAJOCC010000725.1	x_contig	1.653	2.00E-05	4.00E-05	5.00E-01	-1.0000
ENA|CAJOCC010000729|CAJOCC010000729.1	x_contig	6.582	1.00E-05	2.00E-05	5.00E-01	-1.0000
ENA|CAJOCC010000809|CAJOCC010000809.1	x_contig	47.771	1.00E-05	2.00E-05	5.00E-01	-1.0000
ENA|CAJOCC010000854|CAJOCC010000854.1	x_contig	1.69	1.00E-05	2.00E-05	5.00E-01	-1.0000
ENA|CAJOCC010000891|CAJOCC010000891.1	x_contig	15.075	1.00E-05	2.00E-05	5.00E-01	-1.0000
ENA|CAJOCC010000899|CAJOCC010000899.1	x_contig	29.779	1.00E-05	2.00E-05	5.00E-01	-1.0000
ENA|CAJOCC010000922|CAJOCC010000922.1	x_contig	96.744	5.00E-05	1.00E-04	5.00E-01	-1.0000
ENA|CAJOCC010000941|CAJOCC010000941.1	x_contig	41.988	2.00E-05	4.00E-05	5.00E-01	-1.0000
ENA|CAJOCC010000977|CAJOCC010000977.1	x_contig	9.425	1.00E-05	2.00E-05	5.00E-01	-1.0000
ENA|CAJOCC010000988|CAJOCC010000988.1	x_contig	66.424	3.00E-05	6.00E-05	5.00E-01	-1.0000
ENA|CAJOCC010001014|CAJOCC010001014.1	x_contig	11.302	1.00E-05	2.00E-05	5.00E-01	-1.0000
ENA|CAJOCC010001054|CAJOCC010001054.1	x_contig	7.599	1.00E-05	2.00E-05	5.00E-01	-1.0000
ENA|CAJOCC010001058|CAJOCC010001058.1	x_contig	32.457	2.00E-05	4.00E-05	5.00E-01	-1.0000
ENA|CAJOCC010001065|CAJOCC010001065.1	x_contig	0.578	2.00E-05	4.00E-05	5.00E-01	-1.0000
ENA|CAJOCC010001167|CAJOCC010001167.1	x_contig	23	1.00E-05	2.00E-05	5.00E-01	-1.0000
ENA|CAJOCC010001199|CAJOCC010001199.1	x_contig	15.088	1.00E-05	2.00E-05	5.00E-01	-1.0000
ENA|CAJOCC010001310|CAJOCC010001310.1	x_contig	42.475	3.00E-05	6.00E-05	5.00E-01	-1.0000
ENA|CAJOCC010001321|CAJOCC010001321.1	x_contig	9.771	1.00E-05	2.00E-05	5.00E-01	-1.0000

ENA|CAJOCC010001350|CAJOCC010001350.1	x_contig	4.264	3.00E-05	6.00E-05	5.00E-01	-1.0000
ENA|CAJOCC010001351|CAJOCC010001351.1	x_contig	12.786	1.00E-05	2.00E-05	5.00E-01	-1.0000
ENA|CAJOCC010001371|CAJOCC010001371.1	x_contig	54.333	3.00E-05	6.00E-05	5.00E-01	-1.0000
ENA|CAJOCC010001375|CAJOCC010001375.1	x_contig	16.159	1.00E-05	2.00E-05	5.00E-01	-1.0000
ENA|CAJOCC010001389|CAJOCC010001389.1	x_contig	61.229	1.00E-05	2.00E-05	5.00E-01	-1.0000
ENA|CAJOCC010001390|CAJOCC010001390.1	x_contig	17.491	1.00E-05	2.00E-05	5.00E-01	-1.0000
ENA|CAJOCC010001403|CAJOCC010001403.1	x_contig	11.473	1.00E-05	2.00E-05	5.00E-01	-1.0000
ENA|CAJOCC010001423|CAJOCC010001423.1	x_contig	34.836	1.00E-05	2.00E-05	5.00E-01	-1.0000
ENA|CAJOCC010001426|CAJOCC010001426.1	x_contig	26.34	3.00E-05	6.00E-05	5.00E-01	-1.0000
ENA|CAJOCC010001433|CAJOCC010001433.1	x_contig	29.656	1.00E-05	2.00E-05	5.00E-01	-1.0000
ENA|CAJOCC010001492|CAJOCC010001492.1	x_contig	24.045	1.00E-05	2.00E-05	5.00E-01	-1.0000
ENA|CAJOCC010001501|CAJOCC010001501.1	x_contig	107.707	6.00E-05	0.00012	5.00E-01	-1.0000
ENA|CAJOCC010001510|CAJOCC010001510.1	x_contig	29.049	1.00E-05	2.00E-05	5.00E-01	-1.0000
ENA|CAJOCC010001520|CAJOCC010001520.1	x_contig	99.584	6.00E-05	0.00012	5.00E-01	-1.0000
ENA|CAJOCC010001560|CAJOCC010001560.1	x_contig	19.224	1.00E-05	2.00E-05	5.00E-01	-1.0000
ENA|CAJOCC010001578|CAJOCC010001578.1	x_contig	19.248	1.00E-05	2.00E-05	5.00E-01	-1.0000
ENA|CAJOCC010001608|CAJOCC010001608.1	x_contig	182.133	1.00E-04	2.00E-04	5.00E-01	-1.0000
ENA|CAJOCC010001744|CAJOCC010001744.1	x_contig	46.09	2.00E-05	4.00E-05	5.00E-01	-1.0000
ENA|CAJOCC010001760|CAJOCC010001760.1	x_contig	46.707	2.00E-05	4.00E-05	5.00E-01	-1.0000
ENA|CAJOCC010001768|CAJOCC010001768.1	x_contig	14.268	1.00E-05	2.00E-05	5.00E-01	-1.0000
ENA|CAJOCC010001836|CAJOCC010001836.1	x_contig	28.994	2.00E-05	4.00E-05	5.00E-01	-1.0000
ENA|CAJOCC010001858|CAJOCC010001858.1	x_contig	59.087	5.00E-05	1.00E-04	5.00E-01	-1.0000
ENA|CAJOCC010001865|CAJOCC010001865.1	x_contig	101.092	7.00E-05	0.00014	5.00E-01	-1.0000
ENA|CAJOCC010001285|CAJOCC010001285.1	x_contig	501.905	0.00035	0.00068	5.15E-01	-0.9582
ENA|CAJOCC010000875|CAJOCC010000875.1	x_contig	717.29	0.00047	0.00091	5.16E-01	-0.9532
ENA|CAJOCC010001134|CAJOCC010001134.1	x_contig	214.903	0.00015	0.00029	5.17E-01	-0.9511
ENA|CAJOCC010000707|CAJOCC010000707.1	x_contig	193.761	0.00014	0.00027	5.19E-01	-0.9475
ENA|CAJOCC010001566|CAJOCC010001566.1	x_contig	220.451	0.00013	0.00025	5.20E-01	-0.9434
ENA|CAJOCC010001490|CAJOCC010001490.1	x_contig	358.462	0.00025	0.00048	5.21E-01	-0.9411
ENA|CAJOCC010001647|CAJOCC010001647.1	x_contig	378.999	0.00024	0.00046	5.22E-01	-0.9386
ENA|CAJOCC010000394|CAJOCC010000394.1	x_contig	321.642	0.00022	0.00042	5.24E-01	-0.9329
ENA|CAJOCC010001911|CAJOCC010001911.1	x_contig	506.276	0.00032	0.00061	5.25E-01	-0.9307

ENA|CAJOCC010001851|CAJOCC010001851.1	x_contig	288.336	2.00E-04	0.00038	5.26E-01	-0.9260
ENA|CAJOCC010000018|CAJOCC010000018.1	x_contig	297.246	0.00019	0.00036	5.28E-01	-0.9220
ENA|CAJOCC010001190|CAJOCC010001190.1	x_contig	296.314	0.00019	0.00036	5.28E-01	-0.9220
ENA|CAJOCC010000646|CAJOCC010000646.1	x_contig	146.114	9.00E-05	0.00017	5.29E-01	-0.9175
ENA|CAJOCC010001815|CAJOCC010001815.1	x_contig	277.315	0.00018	0.00034	5.29E-01	-0.9175
ENA|CAJOCC010000885|CAJOCC010000885.1	x_contig	383.668	0.00025	0.00047	5.32E-01	-0.9107
ENA|CAJOCC010000929|CAJOCC010000929.1	x_contig	499.492	0.00033	0.00062	5.32E-01	-0.9098
ENA|CAJOCC010001509|CAJOCC010001509.1	x_contig	508.31	0.00033	0.00062	5.32E-01	-0.9098
ENA|CAJOCC010000231|CAJOCC010000231.1	x_contig	735.516	0.00048	9.00E-04	5.33E-01	-0.9069
ENA|CAJOCC010001307|CAJOCC010001307.1	x_contig	647.471	4.00E-04	0.00075	5.33E-01	-0.9069
ENA|CAJOCC010001583|CAJOCC010001583.1	x_contig	150.74	8.00E-05	0.00015	5.33E-01	-0.9069
ENA|CAJOCC010001643|CAJOCC010001643.1	x_contig	590.954	0.00039	0.00073	5.34E-01	-0.9044
ENA|CAJOCC010001895|CAJOCC010001895.1	x_contig	422.938	0.00038	0.00071	5.35E-01	-0.9018
ENA|CAJOCC010000242|CAJOCC010000242.1	x_contig	449.332	3.00E-04	0.00056	5.36E-01	-0.9005
ENA|CAJOCC010001758|CAJOCC010001758.1	x_contig	222.514	0.00015	0.00028	5.36E-01	-0.9005
ENA|CAJOCC010001849|CAJOCC010001849.1	x_contig	226.302	0.00015	0.00028	5.36E-01	-0.9005
ENA|CAJOCC010001004|CAJOCC010001004.1	x_contig	1099.831	0.00074	0.00138	5.36E-01	-0.8991
ENA|CAJOCC010000465|CAJOCC010000465.1	x_contig	671.792	0.00043	8.00E-04	5.38E-01	-0.8957
ENA|CAJOCC010001719|CAJOCC010001719.1	x_contig	991.437	0.00064	0.00119	5.38E-01	-0.8948
ENA|CAJOCC010000849|CAJOCC010000849.1	x_contig	120.031	7.00E-05	0.00013	5.38E-01	-0.8931
ENA|CAJOCC010001168|CAJOCC010001168.1	x_contig	117.593	7.00E-05	0.00013	5.38E-01	-0.8931
ENA|CAJOCC010001465|CAJOCC010001465.1	x_contig	5.867	7.00E-05	0.00013	5.38E-01	-0.8931
ENA|CAJOCC010001519|CAJOCC010001519.1	x_contig	130.897	7.00E-05	0.00013	5.38E-01	-0.8931
ENA|CAJOCC010000168|CAJOCC010000168.1	x_contig	922.631	0.00069	0.00128	5.39E-01	-0.8915
ENA|CAJOCC010000002|CAJOCC010000002.1	x_contig	655.012	0.00048	0.00089	5.39E-01	-0.8908
ENA|CAJOCC010000190|CAJOCC010000190.1	x_contig	452.658	0.00027	5.00E-04	5.40E-01	-0.8890
ENA|CAJOCC010001407|CAJOCC010001407.1	x_contig	677.989	0.00044	0.00081	5.43E-01	-0.8804
ENA|CAJOCC010000275|CAJOCC010000275.1	x_contig	93.95	6.00E-05	0.00011	5.45E-01	-0.8745
ENA|CAJOCC010001379|CAJOCC010001379.1	x_contig	281.445	0.00018	0.00033	5.45E-01	-0.8745
ENA|CAJOCC010001532|CAJOCC010001532.1	x_contig	10.597	6.00E-05	0.00011	5.45E-01	-0.8745
ENA|CAJOCC010001830|CAJOCC010001830.1	x_contig	190.173	0.00012	0.00022	5.45E-01	-0.8745
ENA|CAJOCC010001691|CAJOCC010001691.1	x_contig	248.273	0.00017	0.00031	5.48E-01	-0.8667

ENA|CAJOCC010001400|CAJOCC010001400.1	x_contig	408.778	0.00028	0.00051	5.49E-01	-0.8651
ENA|CAJOCC010001695|CAJOCC010001695.1	x_contig	799.185	0.00056	0.00102	5.49E-01	-0.8651
ENA|CAJOCC010000565|CAJOCC010000565.1	x_contig	126.265	0.00011	2.00E-04	5.50E-01	-0.8625
ENA|CAJOCC010001430|CAJOCC010001430.1	x_contig	150.328	1.00E-04	0.00018	5.56E-01	-0.8480
ENA|CAJOCC010001454|CAJOCC010001454.1	x_contig	111.404	5.00E-05	9.00E-05	5.56E-01	-0.8480
ENA|CAJOCC010000558|CAJOCC010000558.1	x_contig	136.957	9.00E-05	0.00016	5.63E-01	-0.8301
ENA|CAJOCC010000948|CAJOCC010000948.1	x_contig	131.66	9.00E-05	0.00016	5.63E-01	-0.8301
ENA|CAJOCC010001607|CAJOCC010001607.1	x_contig	383.343	0.00026	0.00046	5.65E-01	-0.8231
ENA|CAJOCC010000102|CAJOCC010000102.1	x_contig	381.992	0.00024	0.00042	5.71E-01	-0.8074
ENA|CAJOCC010001574|CAJOCC010001574.1	x_contig	549.258	0.00036	0.00063	5.71E-01	-0.8074
ENA|CAJOCC010000449|CAJOCC010000449.1	x_contig	58.144	4.00E-05	7.00E-05	5.71E-01	-0.8074
ENA|CAJOCC010000965|CAJOCC010000965.1	x_contig	131.774	8.00E-05	0.00014	5.71E-01	-0.8074
ENA|CAJOCC010001355|CAJOCC010001355.1	x_contig	61.123	4.00E-05	7.00E-05	5.71E-01	-0.8074
ENA|CAJOCC010001485|CAJOCC010001485.1	x_contig	4.639	4.00E-05	7.00E-05	5.71E-01	-0.8074
ENA|CAJOCC010001765|CAJOCC010001765.1	x_contig	107.35	8.00E-05	0.00014	5.71E-01	-0.8074
ENA|CAJOCC010000658|CAJOCC010000658.1	x_contig	908.343	0.00063	0.0011	5.73E-01	-0.8041
ENA|CAJOCC010000067|CAJOCC010000067.1	x_contig	1195.029	8.00E-04	0.00139	5.76E-01	-0.7970
ENA|CAJOCC010001710|CAJOCC010001710.1	x_contig	181.155	0.00014	0.00024	5.83E-01	-0.7776
ENA|CAJOCC010000918|CAJOCC010000918.1	x_contig	799.227	0.00052	0.00089	5.84E-01	-0.7753
ENA|CAJOCC010001604|CAJOCC010001604.1	x_contig	24.39	0.00041	7.00E-04	5.86E-01	-0.7717
ENA|CAJOCC010001506|CAJOCC010001506.1	x_contig	425.993	0.00034	0.00058	5.86E-01	-0.7705
ENA|CAJOCC010001441|CAJOCC010001441.1	x_contig	93.558	3.00E-04	0.00051	5.88E-01	-0.7655
ENA|CAJOCC010000418|CAJOCC010000418.1	x_contig	196.871	0.00016	0.00027	5.93E-01	-0.7549
ENA|CAJOCC010000399|CAJOCC010000399.1	x_contig	119.859	0.00019	0.00032	5.94E-01	-0.7521
ENA|CAJOCC010000780|CAJOCC010000780.1	x_contig	248.858	0.00019	0.00032	5.94E-01	-0.7521
ENA|CAJOCC010000071|CAJOCC010000071.1	x_contig	65.75	6.00E-05	1.00E-04	6.00E-01	-0.7370
ENA|CAJOCC010000243|CAJOCC010000243.1	x_contig	23.775	3.00E-05	5.00E-05	6.00E-01	-0.7370
ENA|CAJOCC010000456|CAJOCC010000456.1	x_contig	2.156	3.00E-05	5.00E-05	6.00E-01	-0.7370
ENA|CAJOCC010000739|CAJOCC010000739.1	x_contig	164.851	0.00012	2.00E-04	6.00E-01	-0.7370
ENA|CAJOCC010000801|CAJOCC010000801.1	x_contig	27.693	3.00E-05	5.00E-05	6.00E-01	-0.7370
ENA|CAJOCC010000853|CAJOCC010000853.1	x_contig	10.308	3.00E-05	5.00E-05	6.00E-01	-0.7370
ENA|CAJOCC010000963|CAJOCC010000963.1	x_contig	2.874	3.00E-05	5.00E-05	6.00E-01	-0.7370

ENA|CAJOCC010000993|CAJOCC010000993.1	x_contig	42.824	3.00E-05	5.00E-05	6.00E-01	-0.7370
ENA|CAJOCC010001055|CAJOCC010001055.1	x_contig	60.329	3.00E-05	5.00E-05	6.00E-01	-0.7370
ENA|CAJOCC010001339|CAJOCC010001339.1	x_contig	64.366	3.00E-05	5.00E-05	6.00E-01	-0.7370
ENA|CAJOCC010001420|CAJOCC010001420.1	x_contig	279.776	0.00024	4.00E-04	6.00E-01	-0.7370
ENA|CAJOCC010001644|CAJOCC010001644.1	x_contig	57.03	3.00E-05	5.00E-05	6.00E-01	-0.7370
ENA|CAJOCC010001577|CAJOCC010001577.1	x_contig	19.603	0.00014	0.00023	6.09E-01	-0.7162
ENA|CAJOCC010001028|CAJOCC010001028.1	x_contig	576.385	0.00056	0.00091	6.15E-01	-0.7004
ENA|CAJOCC010001415|CAJOCC010001415.1	x_contig	14.414	8.00E-05	0.00013	6.15E-01	-0.7004
ENA|CAJOCC010000774|CAJOCC010000774.1	x_contig	1.88	5.00E-05	8.00E-05	6.25E-01	-0.6781
ENA|CAJOCC010000631|CAJOCC010000631.1	x_contig	283.143	0.00022	0.00035	6.29E-01	-0.6699
ENA|CAJOCC010001717|CAJOCC010001717.1	x_contig	156.926	0.00017	0.00027	6.30E-01	-0.6674
ENA|CAJOCC010001140|CAJOCC010001140.1	x_contig	457.104	0.00118	0.00185	6.38E-01	-0.6487
ENA|CAJOCC010000979|CAJOCC010000979.1	x_contig	128.852	0.00011	0.00017	6.47E-01	-0.6280
ENA|CAJOCC010000655|CAJOCC010000655.1	x_contig	14.874	0.00015	0.00023	6.52E-01	-0.6167
ENA|CAJOCC010000061|CAJOCC010000061.1	autosomal	26.013	2.00E-05	3.00E-05	6.67E-01	-0.5850
ENA|CAJOCC010000066|CAJOCC010000066.1	autosomal	29.272	2.00E-05	3.00E-05	6.67E-01	-0.5850
ENA|CAJOCC010000108|CAJOCC010000108.1	autosomal	28.8	2.00E-05	3.00E-05	6.67E-01	-0.5850
ENA|CAJOCC010000120|CAJOCC010000120.1	autosomal	26.489	2.00E-05	3.00E-05	6.67E-01	-0.5850
ENA|CAJOCC010000188|CAJOCC010000188.1	autosomal	67.274	4.00E-05	6.00E-05	6.67E-01	-0.5850
ENA|CAJOCC010000251|CAJOCC010000251.1	autosomal	14.231	4.00E-05	6.00E-05	6.67E-01	-0.5850
ENA|CAJOCC010000365|CAJOCC010000365.1	autosomal	22.905	2.00E-05	3.00E-05	6.67E-01	-0.5850
ENA|CAJOCC010000458|CAJOCC010000458.1	autosomal	98.372	8.00E-05	0.00012	6.67E-01	-0.5850
ENA|CAJOCC010000490|CAJOCC010000490.1	autosomal	23.157	2.00E-05	3.00E-05	6.67E-01	-0.5850
ENA|CAJOCC010000518|CAJOCC010000518.1	autosomal	20.975	2.00E-05	3.00E-05	6.67E-01	-0.5850
ENA|CAJOCC010000528|CAJOCC010000528.1	autosomal	29.058	2.00E-05	3.00E-05	6.67E-01	-0.5850
ENA|CAJOCC010000547|CAJOCC010000547.1	autosomal	42.802	2.00E-05	3.00E-05	6.67E-01	-0.5850
ENA|CAJOCC010000615|CAJOCC010000615.1	autosomal	22.994	2.00E-05	3.00E-05	6.67E-01	-0.5850
ENA|CAJOCC010000659|CAJOCC010000659.1	autosomal	8.206	4.00E-05	6.00E-05	6.67E-01	-0.5850
ENA|CAJOCC010000692|CAJOCC010000692.1	autosomal	55.813	4.00E-05	6.00E-05	6.67E-01	-0.5850
ENA|CAJOCC010000791|CAJOCC010000791.1	autosomal	40.401	2.00E-05	3.00E-05	6.67E-01	-0.5850
ENA|CAJOCC010001123|CAJOCC010001123.1	autosomal	32.604	2.00E-05	3.00E-05	6.67E-01	-0.5850
ENA|CAJOCC010001131|CAJOCC010001131.1	autosomal	14.755	2.00E-05	3.00E-05	6.67E-01	-0.5850

ENA|CAJOCC010001150|CAJOCC010001150.1	autosomal	70.636	6.00E-05	9.00E-05	6.67E-01	-0.5850
ENA|CAJOCC010001256|CAJOCC010001256.1	autosomal	1.014	4.00E-05	6.00E-05	6.67E-01	-0.5850
ENA|CAJOCC010001274|CAJOCC010001274.1	autosomal	18.783	2.00E-05	3.00E-05	6.67E-01	-0.5850
ENA|CAJOCC010001299|CAJOCC010001299.1	autosomal	5.16	2.00E-05	3.00E-05	6.67E-01	-0.5850
ENA|CAJOCC010001416|CAJOCC010001416.1	autosomal	25.012	2.00E-05	3.00E-05	6.67E-01	-0.5850
ENA|CAJOCC010001440|CAJOCC010001440.1	autosomal	6.777	2.00E-05	3.00E-05	6.67E-01	-0.5850
ENA|CAJOCC010001481|CAJOCC010001481.1	autosomal	48.837	4.00E-05	6.00E-05	6.67E-01	-0.5850
ENA|CAJOCC010001504|CAJOCC010001504.1	autosomal	52.321	2.00E-05	3.00E-05	6.67E-01	-0.5850
ENA|CAJOCC010001518|CAJOCC010001518.1	autosomal	18.963	2.00E-05	3.00E-05	6.67E-01	-0.5850
ENA|CAJOCC010001554|CAJOCC010001554.1	autosomal	35.122	2.00E-05	3.00E-05	6.67E-01	-0.5850
ENA|CAJOCC010001582|CAJOCC010001582.1	autosomal	41.271	2.00E-05	3.00E-05	6.67E-01	-0.5850
ENA|CAJOCC010001601|CAJOCC010001601.1	autosomal	25.454	2.00E-05	3.00E-05	6.67E-01	-0.5850
ENA|CAJOCC010001773|CAJOCC010001773.1	autosomal	24.618	2.00E-05	3.00E-05	6.67E-01	-0.5850
ENA|CAJOCC010001832|CAJOCC010001832.1	autosomal	45.497	2.00E-05	3.00E-05	6.67E-01	-0.5850
ENA|CAJOCC010001907|CAJOCC010001907.1	autosomal	29.187	2.00E-05	3.00E-05	6.67E-01	-0.5850
ENA|CAJOCC010000855|CAJOCC010000855.1	autosomal	17.432	1.00E-04	0.00015	6.67E-01	-0.5850
ENA|CAJOCC010001825|CAJOCC010001825.1	autosomal	121.972	1.00E-04	0.00015	6.67E-01	-0.5850
ENA|CAJOCC010001419|CAJOCC010001419.1	autosomal	15.757	0.00013	0.00019	6.84E-01	-0.5475
ENA|CAJOCC010001174|CAJOCC010001174.1	autosomal	23.146	0.00026	0.00037	7.03E-01	-0.5090
ENA|CAJOCC010000043|CAJOCC010000043.1	autosomal	20.915	5.00E-05	7.00E-05	7.14E-01	-0.4854
ENA|CAJOCC010000295|CAJOCC010000295.1	autosomal	39.24	5.00E-05	7.00E-05	7.14E-01	-0.4854
ENA|CAJOCC010000834|CAJOCC010000834.1	autosomal	3.079	5.00E-05	7.00E-05	7.14E-01	-0.4854
ENA|CAJOCC010001110|CAJOCC010001110.1	autosomal	67.082	1.00E-04	0.00014	7.14E-01	-0.4854
ENA|CAJOCC010000007|CAJOCC010000007.1	autosomal	56.14	0.00026	0.00036	7.22E-01	-0.4695
ENA|CAJOCC010001724|CAJOCC010001724.1	autosomal	50.025	0.00011	0.00015	7.33E-01	-0.4475
ENA|CAJOCC010000352|CAJOCC010000352.1	autosomal	29.626	3.00E-05	4.00E-05	7.50E-01	-0.4150
ENA|CAJOCC010000527|CAJOCC010000527.1	autosomal	59.43	6.00E-05	8.00E-05	7.50E-01	-0.4150
ENA|CAJOCC010000542|CAJOCC010000542.1	autosomal	13.952	3.00E-05	4.00E-05	7.50E-01	-0.4150
ENA|CAJOCC010000568|CAJOCC010000568.1	autosomal	15.904	3.00E-05	4.00E-05	7.50E-01	-0.4150
ENA|CAJOCC010000667|CAJOCC010000667.1	autosomal	43.397	3.00E-05	4.00E-05	7.50E-01	-0.4150
ENA|CAJOCC010001035|CAJOCC010001035.1	autosomal	1.234	3.00E-05	4.00E-05	7.50E-01	-0.4150
ENA|CAJOCC010001056|CAJOCC010001056.1	autosomal	24.117	3.00E-05	4.00E-05	7.50E-01	-0.4150

ENA|CAJOCC010001111|CAJOCC010001111.1	autosomal	66.142	3.00E-05	4.00E-05	7.50E-01	-0.4150
ENA|CAJOCC010001369|CAJOCC010001369.1	autosomal	22.774	3.00E-05	4.00E-05	7.50E-01	-0.4150
ENA|CAJOCC010001405|CAJOCC010001405.1	autosomal	4.211	3.00E-05	4.00E-05	7.50E-01	-0.4150
ENA|CAJOCC010001475|CAJOCC010001475.1	autosomal	39.325	3.00E-05	4.00E-05	7.50E-01	-0.4150
ENA|CAJOCC010001500|CAJOCC010001500.1	autosomal	27.294	3.00E-05	4.00E-05	7.50E-01	-0.4150
ENA|CAJOCC010001511|CAJOCC010001511.1	autosomal	35.401	3.00E-05	4.00E-05	7.50E-01	-0.4150
ENA|CAJOCC010001594|CAJOCC010001594.1	autosomal	42.536	3.00E-05	4.00E-05	7.50E-01	-0.4150
ENA|CAJOCC010001740|CAJOCC010001740.1	autosomal	47.892	3.00E-05	4.00E-05	7.50E-01	-0.4150
ENA|CAJOCC010000470|CAJOCC010000470.1	autosomal	40.822	9.00E-05	0.00012	7.50E-01	-0.4150
ENA|CAJOCC010001499|CAJOCC010001499.1	autosomal	799.463	0.0014	0.00185	7.57E-01	-0.4021
ENA|CAJOCC010001787|CAJOCC010001787.1	autosomal	349.364	6.00E-04	0.00079	7.59E-01	-0.3969
ENA|CAJOCC010000358|CAJOCC010000358.1	autosomal	116.357	0.00023	3.00E-04	7.67E-01	-0.3833
ENA|CAJOCC010001540|CAJOCC010001540.1	autosomal	20.031	7.00E-05	9.00E-05	7.78E-01	-0.3626
ENA|CAJOCC010000148|CAJOCC010000148.1	autosomal	509.365	0.00107	0.00136	7.87E-01	-0.3460
ENA|CAJOCC010001674|CAJOCC010001674.1	autosomal	50.489	0.00125	0.00158	7.91E-01	-0.3380
ENA|CAJOCC010000475|CAJOCC010000475.1	autosomal	137.262	0.00023	0.00029	7.93E-01	-0.3344
ENA|CAJOCC010001401|CAJOCC010001401.1	autosomal	198.088	0.00035	0.00044	7.95E-01	-0.3301
ENA|CAJOCC010000133|CAJOCC010000133.1	autosomal	42.015	4.00E-05	5.00E-05	8.00E-01	-0.3219
ENA|CAJOCC010000291|CAJOCC010000291.1	autosomal	92.86	8.00E-05	1.00E-04	8.00E-01	-0.3219
ENA|CAJOCC010000670|CAJOCC010000670.1	autosomal	3.116	4.00E-05	5.00E-05	8.00E-01	-0.3219
ENA|CAJOCC010000778|CAJOCC010000778.1	autosomal	20.635	4.00E-05	5.00E-05	8.00E-01	-0.3219
ENA|CAJOCC010000866|CAJOCC010000866.1	autosomal	23.245	4.00E-05	5.00E-05	8.00E-01	-0.3219
ENA|CAJOCC010001337|CAJOCC010001337.1	autosomal	18.979	4.00E-05	5.00E-05	8.00E-01	-0.3219
ENA|CAJOCC010001393|CAJOCC010001393.1	autosomal	73.228	8.00E-05	1.00E-04	8.00E-01	-0.3219
ENA|CAJOCC010001404|CAJOCC010001404.1	autosomal	44.798	4.00E-05	5.00E-05	8.00E-01	-0.3219
ENA|CAJOCC010001514|CAJOCC010001514.1	autosomal	9.689	4.00E-05	5.00E-05	8.00E-01	-0.3219
ENA|CAJOCC010001530|CAJOCC010001530.1	autosomal	17.424	8.00E-05	1.00E-04	8.00E-01	-0.3219
ENA|CAJOCC010001595|CAJOCC010001595.1	autosomal	125.216	8.00E-05	1.00E-04	8.00E-01	-0.3219
ENA|CAJOCC010001602|CAJOCC010001602.1	autosomal	51.64	4.00E-05	5.00E-05	8.00E-01	-0.3219
ENA|CAJOCC010001622|CAJOCC010001622.1	autosomal	16.589	8.00E-05	1.00E-04	8.00E-01	-0.3219
ENA|CAJOCC010001656|CAJOCC010001656.1	autosomal	53.328	4.00E-05	5.00E-05	8.00E-01	-0.3219
ENA|CAJOCC010000167|CAJOCC010000167.1	autosomal	236.919	0.00037	0.00046	8.04E-01	-0.3141

ENA|CAJOCC010000852|CAJOCC010000852.1	autosomal	252.279	0.00038	0.00047	8.09E-01	-0.3067
ENA|CAJOCC010000122|CAJOCC010000122.1	autosomal	625.53	0.00098	0.00121	8.10E-01	-0.3042
ENA|CAJOCC010001380|CAJOCC010001380.1	autosomal	66.311	0.00013	0.00016	8.13E-01	-0.2996
ENA|CAJOCC010001755|CAJOCC010001755.1	autosomal	518.997	0.00087	0.00107	8.13E-01	-0.2985
ENA|CAJOCC010000165|CAJOCC010000165.1	autosomal	254.667	0.00048	0.00059	8.14E-01	-0.2977
ENA|CAJOCC010000765|CAJOCC010000765.1	autosomal	65.979	9.00E-05	0.00011	8.18E-01	-0.2895
ENA|CAJOCC010001814|CAJOCC010001814.1	autosomal	130.383	0.00032	0.00039	8.21E-01	-0.2854
ENA|CAJOCC010000140|CAJOCC010000140.1	autosomal	440.826	0.00087	0.00106	8.21E-01	-0.2850
ENA|CAJOCC010000274|CAJOCC010000274.1	autosomal	146.018	0.00023	0.00028	8.21E-01	-0.2838
ENA|CAJOCC010000412|CAJOCC010000412.1	autosomal	33.741	0.00014	0.00017	8.24E-01	-0.2801
ENA|CAJOCC010001861|CAJOCC010001861.1	autosomal	367.322	0.00063	0.00076	8.29E-01	-0.2706
ENA|CAJOCC010000363|CAJOCC010000363.1	autosomal	101.804	0.00015	0.00018	8.33E-01	-0.2630
ENA|CAJOCC010000085|CAJOCC010000085.1	autosomal	46.501	5.00E-05	6.00E-05	8.33E-01	-0.2630
ENA|CAJOCC010000210|CAJOCC010000210.1	autosomal	53.666	1.00E-04	0.00012	8.33E-01	-0.2630
ENA|CAJOCC010000384|CAJOCC010000384.1	autosomal	90.202	5.00E-05	6.00E-05	8.33E-01	-0.2630
ENA|CAJOCC010000744|CAJOCC010000744.1	autosomal	45.261	1.00E-04	0.00012	8.33E-01	-0.2630
ENA|CAJOCC010000805|CAJOCC010000805.1	autosomal	37.568	5.00E-05	6.00E-05	8.33E-01	-0.2630
ENA|CAJOCC010000881|CAJOCC010000881.1	autosomal	40.899	5.00E-05	6.00E-05	8.33E-01	-0.2630
ENA|CAJOCC010001374|CAJOCC010001374.1	autosomal	46.298	5.00E-05	6.00E-05	8.33E-01	-0.2630
ENA|CAJOCC010001428|CAJOCC010001428.1	autosomal	58.974	1.00E-04	0.00012	8.33E-01	-0.2630
ENA|CAJOCC010001563|CAJOCC010001563.1	autosomal	25.541	5.00E-05	6.00E-05	8.33E-01	-0.2630
ENA|CAJOCC010001069|CAJOCC010001069.1	autosomal	495.745	0.00077	0.00092	8.37E-01	-0.2568
ENA|CAJOCC010000811|CAJOCC010000811.1	autosomal	166.296	0.00031	0.00037	8.38E-01	-0.2553
ENA|CAJOCC010000679|CAJOCC010000679.1	autosomal	118.357	0.00026	0.00031	8.39E-01	-0.2538
ENA|CAJOCC010001327|CAJOCC010001327.1	autosomal	117.159	0.00021	0.00025	8.40E-01	-0.2515
ENA|CAJOCC010000950|CAJOCC010000950.1	autosomal	263.387	0.00048	0.00057	8.42E-01	-0.2479
ENA|CAJOCC010000790|CAJOCC010000790.1	autosomal	1226.167	0.00212	0.00251	8.45E-01	-0.2436
ENA|CAJOCC010001659|CAJOCC010001659.1	autosomal	947.272	0.00169	0.002	8.45E-01	-0.2430
ENA|CAJOCC010001645|CAJOCC010001645.1	autosomal	223.929	0.00033	0.00039	8.46E-01	-0.2410
ENA|CAJOCC010000942|CAJOCC010000942.1	autosomal	59.164	0.00011	0.00013	8.46E-01	-0.2410
ENA|CAJOCC010000300|CAJOCC010000300.1	autosomal	169.144	0.00017	2.00E-04	8.50E-01	-0.2345
ENA|CAJOCC010001667|CAJOCC010001667.1	autosomal	177.098	0.00034	4.00E-04	8.50E-01	-0.2345

ENA|CAJOCC010001221|CAJOCC010001221.1	autosomal	65.289	0.00023	0.00027	8.52E-01	-0.2313
ENA|CAJOCC010001746|CAJOCC010001746.1	autosomal	373.379	0.00052	0.00061	8.52E-01	-0.2303
ENA|CAJOCC010001749|CAJOCC010001749.1	autosomal	343.145	0.00064	0.00075	8.53E-01	-0.2288
ENA|CAJOCC010000306|CAJOCC010000306.1	autosomal	584.011	0.00105	0.00123	8.54E-01	-0.2283
ENA|CAJOCC010001712|CAJOCC010001712.1	autosomal	181.104	0.00035	0.00041	8.54E-01	-0.2283
ENA|CAJOCC010001805|CAJOCC010001805.1	autosomal	223.981	0.00035	0.00041	8.54E-01	-0.2283
ENA|CAJOCC010000062|CAJOCC010000062.1	autosomal	229.993	0.00048	0.00056	8.57E-01	-0.2224
ENA|CAJOCC010000557|CAJOCC010000557.1	autosomal	51.024	6.00E-05	7.00E-05	8.57E-01	-0.2224
ENA|CAJOCC010001108|CAJOCC010001108.1	autosomal	1.364	6.00E-05	7.00E-05	8.57E-01	-0.2224
ENA|CAJOCC010001690|CAJOCC010001690.1	autosomal	51.383	6.00E-05	7.00E-05	8.57E-01	-0.2224
ENA|CAJOCC010001392|CAJOCC010001392.1	autosomal	1526.437	0.00248	0.00289	8.58E-01	-0.2207
ENA|CAJOCC010000328|CAJOCC010000328.1	autosomal	408.612	0.00049	0.00057	8.60E-01	-0.2182
ENA|CAJOCC010000332|CAJOCC010000332.1	autosomal	312.994	0.00049	0.00057	8.60E-01	-0.2182
ENA|CAJOCC010000443|CAJOCC010000443.1	autosomal	190.419	0.00037	0.00043	8.60E-01	-0.2168
ENA|CAJOCC010001445|CAJOCC010001445.1	autosomal	227.986	0.00038	0.00044	8.64E-01	-0.2115
ENA|CAJOCC010001761|CAJOCC010001761.1	autosomal	233.526	0.00038	0.00044	8.64E-01	-0.2115
ENA|CAJOCC010001925|CAJOCC010001925.1	autosomal	884.054	0.00173	0.002	8.65E-01	-0.2092
ENA|CAJOCC010000162|CAJOCC010000162.1	autosomal	10.29	0.00013	0.00015	8.67E-01	-0.2065
ENA|CAJOCC010001615|CAJOCC010001615.1	autosomal	750.896	0.00132	0.00152	8.68E-01	-0.2035
ENA|CAJOCC010000176|CAJOCC010000176.1	autosomal	412.319	0.00061	7.00E-04	8.71E-01	-0.1985
ENA|CAJOCC010001722|CAJOCC010001722.1	autosomal	602.919	0.00096	0.0011	8.73E-01	-0.1964
ENA|CAJOCC010000211|CAJOCC010000211.1	autosomal	45.041	7.00E-05	8.00E-05	8.75E-01	-0.1926
ENA|CAJOCC010000472|CAJOCC010000472.1	autosomal	86.907	0.00014	0.00016	8.75E-01	-0.1926
ENA|CAJOCC010000938|CAJOCC010000938.1	autosomal	34.495	7.00E-05	8.00E-05	8.75E-01	-0.1926
ENA|CAJOCC010001586|CAJOCC010001586.1	autosomal	102.5	7.00E-05	8.00E-05	8.75E-01	-0.1926
ENA|CAJOCC010001462|CAJOCC010001462.1	autosomal	203.191	0.00049	0.00056	8.75E-01	-0.1926
ENA|CAJOCC010001255|CAJOCC010001255.1	autosomal	744.188	0.00128	0.00146	8.77E-01	-0.1898
ENA|CAJOCC010000286|CAJOCC010000286.1	autosomal	293.093	0.00043	0.00049	8.78E-01	-0.1884
ENA|CAJOCC010001287|CAJOCC010001287.1	autosomal	245.038	0.00044	5.00E-04	8.80E-01	-0.1844
ENA|CAJOCC010000009|CAJOCC010000009.1	autosomal	197.85	3.00E-04	0.00034	8.82E-01	-0.1806
ENA|CAJOCC010001599|CAJOCC010001599.1	autosomal	98.086	0.00015	0.00017	8.82E-01	-0.1806
ENA|CAJOCC010000323|CAJOCC010000323.1	autosomal	172.241	0.00053	6.00E-04	8.83E-01	-0.1790

ENA|CAJOCC010001913|CAJOCC010001913.1	autosomal	447.393	0.00076	0.00086	8.84E-01	-0.1783
ENA|CAJOCC010001022|CAJOCC010001022.1	autosomal	190.483	0.00031	0.00035	8.86E-01	-0.1751
ENA|CAJOCC010001864|CAJOCC010001864.1	autosomal	1556.748	0.00227	0.00256	8.87E-01	-0.1735
ENA|CAJOCC010000146|CAJOCC010000146.1	autosomal	29.079	8.00E-05	9.00E-05	8.89E-01	-0.1699
ENA|CAJOCC010000180|CAJOCC010000180.1	autosomal	73.123	8.00E-05	9.00E-05	8.89E-01	-0.1699
ENA|CAJOCC010000217|CAJOCC010000217.1	autosomal	256.634	0.00032	0.00036	8.89E-01	-0.1699
ENA|CAJOCC010000909|CAJOCC010000909.1	autosomal	31.293	8.00E-05	9.00E-05	8.89E-01	-0.1699
ENA|CAJOCC010001396|CAJOCC010001396.1	autosomal	40.977	0.00296	0.00333	8.89E-01	-0.1699
ENA|CAJOCC010001535|CAJOCC010001535.1	autosomal	67.437	8.00E-05	9.00E-05	8.89E-01	-0.1699
ENA|CAJOCC010001541|CAJOCC010001541.1	autosomal	59.314	8.00E-05	9.00E-05	8.89E-01	-0.1699
ENA|CAJOCC010001692|CAJOCC010001692.1	autosomal	56.806	8.00E-05	9.00E-05	8.89E-01	-0.1699
ENA|CAJOCC010000341|CAJOCC010000341.1	autosomal	512.389	0.00089	0.001	8.90E-01	-0.1681
ENA|CAJOCC010001893|CAJOCC010001893.1	autosomal	1128.673	0.0017	0.00191	8.90E-01	-0.1680
ENA|CAJOCC010001347|CAJOCC010001347.1	autosomal	48.195	5.00E-04	0.00056	8.93E-01	-0.1635
ENA|CAJOCC010001278|CAJOCC010001278.1	autosomal	443.256	0.00084	0.00094	8.94E-01	-0.1623
ENA|CAJOCC010001194|CAJOCC010001194.1	autosomal	15.772	0.00017	0.00019	8.95E-01	-0.1605
ENA|CAJOCC010001694|CAJOCC010001694.1	autosomal	318.212	0.00034	0.00038	8.95E-01	-0.1605
ENA|CAJOCC010001816|CAJOCC010001816.1	autosomal	505.894	0.00094	0.00105	8.95E-01	-0.1597
ENA|CAJOCC010001668|CAJOCC010001668.1	autosomal	382.703	6.00E-04	0.00067	8.96E-01	-0.1592
ENA|CAJOCC010001770|CAJOCC010001770.1	autosomal	503.677	0.00069	0.00077	8.96E-01	-0.1583
ENA|CAJOCC010001026|CAJOCC010001026.1	autosomal	232.111	0.00035	0.00039	8.97E-01	-0.1561
ENA|CAJOCC010001941|CAJOCC010001941.1	autosomal	718.821	0.00105	0.00117	8.97E-01	-0.1561
ENA|CAJOCC010000362|CAJOCC010000362.1	autosomal	24.922	9.00E-05	1.00E-04	9.00E-01	-0.1520
ENA|CAJOCC010000574|CAJOCC010000574.1	autosomal	73.807	9.00E-05	1.00E-04	9.00E-01	-0.1520
ENA|CAJOCC010001889|CAJOCC010001889.1	autosomal	306.351	0.00082	0.00091	9.01E-01	-0.1502
ENA|CAJOCC010001751|CAJOCC010001751.1	autosomal	946.081	0.00158	0.00175	9.03E-01	-0.1474
ENA|CAJOCC010001455|CAJOCC010001455.1	autosomal	166.769	0.00028	0.00031	9.03E-01	-0.1468
ENA|CAJOCC010001593|CAJOCC010001593.1	autosomal	963.425	0.00149	0.00164	9.09E-01	-0.1384
ENA|CAJOCC010001700|CAJOCC010001700.1	autosomal	31.916	2.00E-04	0.00022	9.09E-01	-0.1375
ENA|CAJOCC010001916|CAJOCC010001916.1	autosomal	691.828	0.0013	0.00143	9.09E-01	-0.1375
ENA|CAJOCC010001847|CAJOCC010001847.1	autosomal	565.481	0.00091	0.001	9.10E-01	-0.1361
ENA|CAJOCC010001623|CAJOCC010001623.1	autosomal	547.86	0.00113	0.00124	9.11E-01	-0.1340

ENA|CAJOCC010000488|CAJOCC010000488.1	autosomal	21.567	0.00021	0.00023	9.13E-01	-0.1312
ENA|CAJOCC010001791|CAJOCC010001791.1	autosomal	175.836	0.00021	0.00023	9.13E-01	-0.1312
ENA|CAJOCC010001092|CAJOCC010001092.1	autosomal	659.738	0.00108	0.00118	9.15E-01	-0.1278
ENA|CAJOCC010001207|CAJOCC010001207.1	autosomal	203.08	0.00033	0.00036	9.17E-01	-0.1255
ENA|CAJOCC010000345|CAJOCC010000345.1	autosomal	140.357	0.00022	0.00024	9.17E-01	-0.1255
ENA|CAJOCC010000502|CAJOCC010000502.1	autosomal	83.858	0.00011	0.00012	9.17E-01	-0.1255
ENA|CAJOCC010001013|CAJOCC010001013.1	autosomal	85.068	0.00011	0.00012	9.17E-01	-0.1255
ENA|CAJOCC010001322|CAJOCC010001322.1	autosomal	63.417	0.00011	0.00012	9.17E-01	-0.1255
ENA|CAJOCC010001324|CAJOCC010001324.1	autosomal	75.689	0.00011	0.00012	9.17E-01	-0.1255
ENA|CAJOCC010001661|CAJOCC010001661.1	autosomal	3.566	0.00011	0.00012	9.17E-01	-0.1255
ENA|CAJOCC010000459|CAJOCC010000459.1	autosomal	616.374	0.00096	0.00104	9.23E-01	-0.1155
ENA|CAJOCC010001006|CAJOCC010001006.1	autosomal	87.832	0.00012	0.00013	9.23E-01	-0.1155
ENA|CAJOCC010001038|CAJOCC010001038.1	autosomal	12.431	0.00012	0.00013	9.23E-01	-0.1155
ENA|CAJOCC010001828|CAJOCC010001828.1	autosomal	99.621	0.00012	0.00013	9.23E-01	-0.1155
ENA|CAJOCC010001833|CAJOCC010001833.1	autosomal	134.199	0.00012	0.00013	9.23E-01	-0.1155
ENA|CAJOCC010000298|CAJOCC010000298.1	autosomal	505.821	0.00086	0.00093	9.25E-01	-0.1129
ENA|CAJOCC010000742|CAJOCC010000742.1	autosomal	161.754	0.00026	0.00028	9.29E-01	-0.1069
ENA|CAJOCC010001726|CAJOCC010001726.1	autosomal	142.329	0.00026	0.00028	9.29E-01	-0.1069
ENA|CAJOCC010001919|CAJOCC010001919.1	autosomal	713.949	0.00157	0.00169	9.29E-01	-0.1063
ENA|CAJOCC010000268|CAJOCC010000268.1	autosomal	326.816	0.00054	0.00058	9.31E-01	-0.1031
ENA|CAJOCC010000827|CAJOCC010000827.1	autosomal	172.753	0.00027	0.00029	9.31E-01	-0.1031
ENA|CAJOCC010000623|CAJOCC010000623.1	autosomal	404.685	0.00069	0.00074	9.32E-01	-0.1009
ENA|CAJOCC010000044|CAJOCC010000044.1	autosomal	124.87	0.00014	0.00015	9.33E-01	-0.0995
ENA|CAJOCC010000290|CAJOCC010000290.1	autosomal	159.567	0.00028	3.00E-04	9.33E-01	-0.0995
ENA|CAJOCC010001235|CAJOCC010001235.1	autosomal	107.74	0.00014	0.00015	9.33E-01	-0.0995
ENA|CAJOCC010001766|CAJOCC010001766.1	autosomal	83.738	0.00014	0.00015	9.33E-01	-0.0995
ENA|CAJOCC010001427|CAJOCC010001427.1	autosomal	381.086	0.00057	0.00061	9.34E-01	-0.0978
ENA|CAJOCC010001924|CAJOCC010001924.1	autosomal	707.257	0.001	0.00107	9.35E-01	-0.0976
ENA|CAJOCC010001841|CAJOCC010001841.1	autosomal	194.041	0.00029	0.00031	9.35E-01	-0.0962
ENA|CAJOCC010001902|CAJOCC010001902.1	autosomal	979.101	0.0014	0.00149	9.40E-01	-0.0899
ENA|CAJOCC010000161|CAJOCC010000161.1	autosomal	322.459	0.00047	5.00E-04	9.40E-01	-0.0893
ENA|CAJOCC010000005|CAJOCC010000005.1	autosomal	115.862	0.00016	0.00017	9.41E-01	-0.0875

ENA|CAJOCC010000305|CAJOCC010000305.1	autosomal	743.109	0.00101	0.00107	9.44E-01	-0.0833
ENA|CAJOCC010000318|CAJOCC010000318.1	autosomal	1010.799	0.00152	0.00161	9.44E-01	-0.0830
ENA|CAJOCC010000632|CAJOCC010000632.1	autosomal	218.828	0.00017	0.00018	9.44E-01	-0.0825
ENA|CAJOCC010000131|CAJOCC010000131.1	autosomal	330.56	0.00069	0.00073	9.45E-01	-0.0813
ENA|CAJOCC010001621|CAJOCC010001621.1	autosomal	16.736	0.00018	0.00019	9.47E-01	-0.0780
ENA|CAJOCC010001783|CAJOCC010001783.1	autosomal	614.221	0.00092	0.00097	9.48E-01	-0.0764
ENA|CAJOCC010001576|CAJOCC010001576.1	autosomal	212.852	0.00037	0.00039	9.49E-01	-0.0759
ENA|CAJOCC010000800|CAJOCC010000800.1	autosomal	736.428	0.00113	0.00119	9.50E-01	-0.0746
ENA|CAJOCC010000342|CAJOCC010000342.1	autosomal	276.219	0.00038	4.00E-04	9.50E-01	-0.0740
ENA|CAJOCC010001657|CAJOCC010001657.1	autosomal	279.599	0.00039	0.00041	9.51E-01	-0.0721
ENA|CAJOCC010000256|CAJOCC010000256.1	autosomal	109.223	2.00E-04	0.00021	9.52E-01	-0.0704
ENA|CAJOCC010001590|CAJOCC010001590.1	autosomal	2485.802	0.00362	0.0038	9.53E-01	-0.0700
ENA|CAJOCC010001899|CAJOCC010001899.1	autosomal	1221.291	0.00164	0.00172	9.53E-01	-0.0687
ENA|CAJOCC010001609|CAJOCC010001609.1	autosomal	1967.895	0.003	0.00314	9.55E-01	-0.0658
ENA|CAJOCC010001122|CAJOCC010001122.1	autosomal	155.978	0.00022	0.00023	9.57E-01	-0.0641
ENA|CAJOCC010000202|CAJOCC010000202.1	autosomal	290.711	0.00046	0.00048	9.58E-01	-0.0614
ENA|CAJOCC010000238|CAJOCC010000238.1	autosomal	143.654	0.00023	0.00024	9.58E-01	-0.0614
ENA|CAJOCC010000837|CAJOCC010000837.1	autosomal	138.335	0.00023	0.00024	9.58E-01	-0.0614
ENA|CAJOCC010000029|CAJOCC010000029.1	autosomal	311.922	0.00047	0.00049	9.59E-01	-0.0601
ENA|CAJOCC010001579|CAJOCC010001579.1	autosomal	533.172	0.00075	0.00078	9.62E-01	-0.0566
ENA|CAJOCC010000054|CAJOCC010000054.1	autosomal	154.229	0.00025	0.00026	9.62E-01	-0.0566
ENA|CAJOCC010001771|CAJOCC010001771.1	autosomal	333.769	5.00E-04	0.00052	9.62E-01	-0.0566
ENA|CAJOCC010000441|CAJOCC010000441.1	autosomal	188.58	0.00026	0.00027	9.63E-01	-0.0544
ENA|CAJOCC010000052|CAJOCC010000052.1	autosomal	618.34	0.00079	0.00082	9.63E-01	-0.0538
ENA|CAJOCC010001361|CAJOCC010001361.1	autosomal	200.517	0.00028	0.00029	9.66E-01	-0.0506
ENA|CAJOCC010001874|CAJOCC010001874.1	autosomal	2355.337	0.00343	0.00355	9.66E-01	-0.0496
ENA|CAJOCC010001556|CAJOCC010001556.1	autosomal	208.729	3.00E-04	0.00031	9.68E-01	-0.0473
ENA|CAJOCC010001862|CAJOCC010001862.1	autosomal	43.182	0.00031	0.00032	9.69E-01	-0.0458
ENA|CAJOCC010001078|CAJOCC010001078.1	autosomal	612.613	0.00093	0.00096	9.69E-01	-0.0458
ENA|CAJOCC010001762|CAJOCC010001762.1	autosomal	1052.056	0.00128	0.00132	9.70E-01	-0.0444
ENA|CAJOCC010000096|CAJOCC010000096.1	autosomal	205.808	0.00033	0.00034	9.71E-01	-0.0431
ENA|CAJOCC010000484|CAJOCC010000484.1	autosomal	568.651	0.00066	0.00068	9.71E-01	-0.0431

ENA|CAJOCC010000255|CAJOCC010000255.1	autosomal	286.038	0.00035	0.00036	9.72E-01	-0.0406
ENA|CAJOCC010000515|CAJOCC010000515.1	autosomal	279.325	0.00035	0.00036	9.72E-01	-0.0406
ENA|CAJOCC010000114|CAJOCC010000114.1	autosomal	206.125	0.00036	0.00037	9.73E-01	-0.0395
ENA|CAJOCC010001204|CAJOCC010001204.1	autosomal	190.721	0.00037	0.00038	9.74E-01	-0.0385
ENA|CAJOCC010000390|CAJOCC010000390.1	autosomal	332.994	0.00043	0.00044	9.77E-01	-0.0332
ENA|CAJOCC010000031|CAJOCC010000031.1	autosomal	273.905	0.00044	0.00045	9.78E-01	-0.0324
ENA|CAJOCC010001878|CAJOCC010001878.1	autosomal	1349.055	0.00186	0.0019	9.79E-01	-0.0307
ENA|CAJOCC010001908|CAJOCC010001908.1	autosomal	617.707	0.00097	0.00099	9.80E-01	-0.0294
ENA|CAJOCC010001697|CAJOCC010001697.1	autosomal	363.29	5.00E-04	0.00051	9.80E-01	-0.0286
ENA|CAJOCC010001713|CAJOCC010001713.1	autosomal	348.41	0.00051	0.00052	9.81E-01	-0.0280
ENA|CAJOCC010001354|CAJOCC010001354.1	autosomal	462.895	0.00055	0.00056	9.82E-01	-0.0260
ENA|CAJOCC010001603|CAJOCC010001603.1	autosomal	707.813	0.00113	0.00115	9.83E-01	-0.0253
ENA|CAJOCC010000889|CAJOCC010000889.1	autosomal	4319.735	0.00585	0.00595	9.83E-01	-0.0245
ENA|CAJOCC010000388|CAJOCC010000388.1	autosomal	499.326	6.00E-04	0.00061	9.84E-01	-0.0238
ENA|CAJOCC010001618|CAJOCC010001618.1	autosomal	874.826	0.00128	0.0013	9.85E-01	-0.0224
ENA|CAJOCC010001892|CAJOCC010001892.1	autosomal	954.998	0.00131	0.00133	9.85E-01	-0.0219
ENA|CAJOCC010001939|CAJOCC010001939.1	autosomal	537.573	0.00067	0.00068	9.85E-01	-0.0214
ENA|CAJOCC010001497|CAJOCC010001497.1	autosomal	807.295	0.00135	0.00137	9.85E-01	-0.0212
ENA|CAJOCC010001632|CAJOCC010001632.1	autosomal	1943.207	0.00292	0.00296	9.86E-01	-0.0196
ENA|CAJOCC010001819|CAJOCC010001819.1	autosomal	1537.812	0.00224	0.00227	9.87E-01	-0.0192
ENA|CAJOCC010000457|CAJOCC010000457.1	autosomal	664.854	0.00079	8.00E-04	9.88E-01	-0.0181
ENA|CAJOCC010001688|CAJOCC010001688.1	autosomal	689.789	0.00083	0.00084	9.88E-01	-0.0173
ENA|CAJOCC010000279|CAJOCC010000279.1	autosomal	1232.37	0.00168	0.0017	9.88E-01	-0.0171
ENA|CAJOCC010001792|CAJOCC010001792.1	autosomal	643.883	0.00089	9.00E-04	9.89E-01	-0.0161
ENA|CAJOCC010001646|CAJOCC010001646.1	autosomal	2004.094	0.00281	0.00284	9.89E-01	-0.0153
ENA|CAJOCC010001775|CAJOCC010001775.1	autosomal	704.229	0.00096	0.00097	9.90E-01	-0.0150
ENA|CAJOCC010001605|CAJOCC010001605.1	autosomal	832.335	0.00097	0.00098	9.90E-01	-0.0148
ENA|CAJOCC010001923|CAJOCC010001923.1	autosomal	3196.981	0.00435	0.00439	9.91E-01	-0.0132
ENA|CAJOCC010000840|CAJOCC010000840.1	autosomal	684.103	0.00109	0.0011	9.91E-01	-0.0132
ENA|CAJOCC010000614|CAJOCC010000614.1	autosomal	1840.68	0.00235	0.00237	9.92E-01	-0.0122
ENA|CAJOCC010001658|CAJOCC010001658.1	autosomal	837.082	0.00121	0.00122	9.92E-01	-0.0119
ENA|CAJOCC010001723|CAJOCC010001723.1	autosomal	980.002	0.00137	0.00138	9.93E-01	-0.0105

ENA|CAJOCC010001747|CAJOCC010001747.1	autosomal	1012.335	0.00148	0.00149	9.93E-01	-0.0097
ENA|CAJOCC010001544|CAJOCC010001544.1	autosomal	2133.682	0.00298	0.003	9.93E-01	-0.0097
ENA|CAJOCC010001655|CAJOCC010001655.1	autosomal	1139.526	0.00167	0.00168	9.94E-01	-0.0086
ENA|CAJOCC010001672|CAJOCC010001672.1	autosomal	1899.376	0.00231	0.00232	9.96E-01	-0.0062
ENA|CAJOCC010001414|CAJOCC010001414.1	autosomal	1761.579	0.00271	0.00272	9.96E-01	-0.0053
ENA|CAJOCC010000003|CAJOCC010000003.1	autosomal	16.773	1.00E-05	1.00E-05	1.00E+00	0.0000
ENA|CAJOCC010000004|CAJOCC010000004.1	autosomal	9.473	1.00E-05	1.00E-05	1.00E+00	0.0000
ENA|CAJOCC010000020|CAJOCC010000020.1	autosomal	0.697	1.00E-05	1.00E-05	1.00E+00	0.0000
ENA|CAJOCC010000024|CAJOCC010000024.1	autosomal	5.255	1.00E-05	1.00E-05	1.00E+00	0.0000
ENA|CAJOCC010000028|CAJOCC010000028.1	autosomal	2.131	1.00E-05	1.00E-05	1.00E+00	0.0000
ENA|CAJOCC010000030|CAJOCC010000030.1	autosomal	13.788	1.00E-05	1.00E-05	1.00E+00	0.0000
ENA|CAJOCC010000033|CAJOCC010000033.1	autosomal	8.392	6.00E-05	6.00E-05	1.00E+00	0.0000
ENA|CAJOCC010000037|CAJOCC010000037.1	autosomal	37.406	2.00E-05	2.00E-05	1.00E+00	0.0000
ENA|CAJOCC010000038|CAJOCC010000038.1	autosomal	34.101	4.00E-05	4.00E-05	1.00E+00	0.0000
ENA|CAJOCC010000041|CAJOCC010000041.1	autosomal	11.682	5.00E-05	5.00E-05	1.00E+00	0.0000
ENA|CAJOCC010000042|CAJOCC010000042.1	autosomal	9.199	4.00E-05	4.00E-05	1.00E+00	0.0000
ENA|CAJOCC010000046|CAJOCC010000046.1	autosomal	231.484	0.00031	0.00031	1.00E+00	0.0000
ENA|CAJOCC010000047|CAJOCC010000047.1	autosomal	78.172	9.00E-05	9.00E-05	1.00E+00	0.0000
ENA|CAJOCC010000048|CAJOCC010000048.1	autosomal	18.107	4.00E-05	4.00E-05	1.00E+00	0.0000
ENA|CAJOCC010000064|CAJOCC010000064.1	autosomal	99.39	0.00013	0.00013	1.00E+00	0.0000
ENA|CAJOCC010000068|CAJOCC010000068.1	autosomal	27.982	4.00E-05	4.00E-05	1.00E+00	0.0000
ENA|CAJOCC010000069|CAJOCC010000069.1	autosomal	23.067	2.00E-05	2.00E-05	1.00E+00	0.0000
ENA|CAJOCC010000070|CAJOCC010000070.1	autosomal	53.261	3.00E-05	3.00E-05	1.00E+00	0.0000
ENA|CAJOCC010000084|CAJOCC010000084.1	autosomal	90.107	5.00E-05	5.00E-05	1.00E+00	0.0000
ENA|CAJOCC010000089|CAJOCC010000089.1	autosomal	13.547	1.00E-05	1.00E-05	1.00E+00	0.0000
ENA|CAJOCC010000093|CAJOCC010000093.1	autosomal	11.079	1.00E-05	1.00E-05	1.00E+00	0.0000
ENA|CAJOCC010000100|CAJOCC010000100.1	autosomal	48.8	3.00E-05	3.00E-05	1.00E+00	0.0000
ENA|CAJOCC010000104|CAJOCC010000104.1	autosomal	45.273	4.00E-05	4.00E-05	1.00E+00	0.0000
ENA|CAJOCC010000106|CAJOCC010000106.1	autosomal	1926.533	0.00272	0.00272	1.00E+00	0.0000
ENA|CAJOCC010000107|CAJOCC010000107.1	autosomal	80.086	9.00E-05	9.00E-05	1.00E+00	0.0000
ENA|CAJOCC010000111|CAJOCC010000111.1	autosomal	29.675	2.00E-05	2.00E-05	1.00E+00	0.0000
ENA|CAJOCC010000112|CAJOCC010000112.1	autosomal	94.744	0.00016	0.00016	1.00E+00	0.0000

ENA|CAJOCC010000115|CAJOCC010000115.1	autosomal	79.968	0.00011	0.00011	1.00E+00	0.0000
ENA|CAJOCC010000117|CAJOCC010000117.1	autosomal	9.988	2.00E-05	2.00E-05	1.00E+00	0.0000
ENA|CAJOCC010000121|CAJOCC010000121.1	autosomal	12.309	2.00E-05	2.00E-05	1.00E+00	0.0000
ENA|CAJOCC010000125|CAJOCC010000125.1	autosomal	6.702	1.00E-05	1.00E-05	1.00E+00	0.0000
ENA|CAJOCC010000127|CAJOCC010000127.1	autosomal	100.34	0.00012	0.00012	1.00E+00	0.0000
ENA|CAJOCC010000151|CAJOCC010000151.1	autosomal	42.622	3.00E-05	3.00E-05	1.00E+00	0.0000
ENA|CAJOCC010000153|CAJOCC010000153.1	autosomal	47.244	0.00024	0.00024	1.00E+00	0.0000
ENA|CAJOCC010000155|CAJOCC010000155.1	autosomal	336.901	0.00061	0.00061	1.00E+00	0.0000
ENA|CAJOCC010000156|CAJOCC010000156.1	autosomal	3.375	5.00E-05	5.00E-05	1.00E+00	0.0000
ENA|CAJOCC010000184|CAJOCC010000184.1	autosomal	25.405	1.00E-05	1.00E-05	1.00E+00	0.0000
ENA|CAJOCC010000195|CAJOCC010000195.1	autosomal	11.948	1.00E-05	1.00E-05	1.00E+00	0.0000
ENA|CAJOCC010000198|CAJOCC010000198.1	autosomal	24.464	5.00E-05	5.00E-05	1.00E+00	0.0000
ENA|CAJOCC010000207|CAJOCC010000207.1	autosomal	43.085	4.00E-05	4.00E-05	1.00E+00	0.0000
ENA|CAJOCC010000212|CAJOCC010000212.1	autosomal	1.342	1.00E-05	1.00E-05	1.00E+00	0.0000
ENA|CAJOCC010000216|CAJOCC010000216.1	autosomal	3.304	2.00E-05	2.00E-05	1.00E+00	0.0000
ENA|CAJOCC010000222|CAJOCC010000222.1	autosomal	60.064	7.00E-05	7.00E-05	1.00E+00	0.0000
ENA|CAJOCC010000223|CAJOCC010000223.1	autosomal	227.156	0.00033	0.00033	1.00E+00	0.0000
ENA|CAJOCC010000229|CAJOCC010000229.1	autosomal	198.376	0.00023	0.00023	1.00E+00	0.0000
ENA|CAJOCC010000230|CAJOCC010000230.1	autosomal	25.377	4.00E-05	4.00E-05	1.00E+00	0.0000
ENA|CAJOCC010000232|CAJOCC010000232.1	autosomal	8.387	3.00E-05	3.00E-05	1.00E+00	0.0000
ENA|CAJOCC010000240|CAJOCC010000240.1	autosomal	5.183	1.00E-05	1.00E-05	1.00E+00	0.0000
ENA|CAJOCC010000241|CAJOCC010000241.1	autosomal	7.874	1.00E-05	1.00E-05	1.00E+00	0.0000
ENA|CAJOCC010000246|CAJOCC010000246.1	autosomal	14.498	2.00E-05	2.00E-05	1.00E+00	0.0000
ENA|CAJOCC010000247|CAJOCC010000247.1	autosomal	1.544	1.00E-05	1.00E-05	1.00E+00	0.0000
ENA|CAJOCC010000249|CAJOCC010000249.1	autosomal	25.728	2.00E-05	2.00E-05	1.00E+00	0.0000
ENA|CAJOCC010000258|CAJOCC010000258.1	autosomal	16.292	1.00E-05	1.00E-05	1.00E+00	0.0000
ENA|CAJOCC010000261|CAJOCC010000261.1	autosomal	282.886	0.00037	0.00037	1.00E+00	0.0000
ENA|CAJOCC010000276|CAJOCC010000276.1	autosomal	4.975	1.00E-05	1.00E-05	1.00E+00	0.0000
ENA|CAJOCC010000281|CAJOCC010000281.1	autosomal	490.785	0.00064	0.00064	1.00E+00	0.0000
ENA|CAJOCC010000284|CAJOCC010000284.1	autosomal	24.055	1.00E-05	1.00E-05	1.00E+00	0.0000
ENA|CAJOCC010000294|CAJOCC010000294.1	autosomal	85.771	0.00012	0.00012	1.00E+00	0.0000
ENA|CAJOCC010000301|CAJOCC010000301.1	autosomal	41.657	4.00E-05	4.00E-05	1.00E+00	0.0000

ENA|CAJOCC010000302|CAJOCC010000302.1	autosomal	24.82	1.00E-05	1.00E-05	1.00E+00	0.0000
ENA|CAJOCC010000303|CAJOCC010000303.1	autosomal	42.561	5.00E-05	5.00E-05	1.00E+00	0.0000
ENA|CAJOCC010000304|CAJOCC010000304.1	autosomal	139.153	0.00019	0.00019	1.00E+00	0.0000
ENA|CAJOCC010000310|CAJOCC010000310.1	autosomal	5.113	4.00E-05	4.00E-05	1.00E+00	0.0000
ENA|CAJOCC010000322|CAJOCC010000322.1	autosomal	202.769	0.00026	0.00026	1.00E+00	0.0000
ENA|CAJOCC010000334|CAJOCC010000334.1	autosomal	23.438	2.00E-05	2.00E-05	1.00E+00	0.0000
ENA|CAJOCC010000343|CAJOCC010000343.1	autosomal	4.425	1.00E-05	1.00E-05	1.00E+00	0.0000
ENA|CAJOCC010000347|CAJOCC010000347.1	autosomal	327.964	0.00042	0.00042	1.00E+00	0.0000
ENA|CAJOCC010000348|CAJOCC010000348.1	autosomal	1184.928	0.00143	0.00143	1.00E+00	0.0000
ENA|CAJOCC010000349|CAJOCC010000349.1	autosomal	46.36	3.00E-05	3.00E-05	1.00E+00	0.0000
ENA|CAJOCC010000350|CAJOCC010000350.1	autosomal	56.342	2.00E-05	2.00E-05	1.00E+00	0.0000
ENA|CAJOCC010000354|CAJOCC010000354.1	autosomal	123.723	0.00015	0.00015	1.00E+00	0.0000
ENA|CAJOCC010000356|CAJOCC010000356.1	autosomal	19.895	1.00E-05	1.00E-05	1.00E+00	0.0000
ENA|CAJOCC010000357|CAJOCC010000357.1	autosomal	83.966	6.00E-05	6.00E-05	1.00E+00	0.0000
ENA|CAJOCC010000361|CAJOCC010000361.1	autosomal	120.638	0.00016	0.00016	1.00E+00	0.0000
ENA|CAJOCC010000366|CAJOCC010000366.1	autosomal	26.404	1.00E-05	1.00E-05	1.00E+00	0.0000
ENA|CAJOCC010000371|CAJOCC010000371.1	autosomal	21.159	2.00E-05	2.00E-05	1.00E+00	0.0000
ENA|CAJOCC010000372|CAJOCC010000372.1	autosomal	10.302	1.00E-05	1.00E-05	1.00E+00	0.0000
ENA|CAJOCC010000374|CAJOCC010000374.1	autosomal	22.106	1.00E-05	1.00E-05	1.00E+00	0.0000
ENA|CAJOCC010000376|CAJOCC010000376.1	autosomal	11.086	4.00E-05	4.00E-05	1.00E+00	0.0000
ENA|CAJOCC010000378|CAJOCC010000378.1	autosomal	36.032	3.00E-05	3.00E-05	1.00E+00	0.0000
ENA|CAJOCC010000386|CAJOCC010000386.1	autosomal	5.997	6.00E-05	6.00E-05	1.00E+00	0.0000
ENA|CAJOCC010000387|CAJOCC010000387.1	autosomal	17.547	1.00E-05	1.00E-05	1.00E+00	0.0000
ENA|CAJOCC010000391|CAJOCC010000391.1	autosomal	48.806	4.00E-05	4.00E-05	1.00E+00	0.0000
ENA|CAJOCC010000397|CAJOCC010000397.1	autosomal	1.168	3.00E-05	3.00E-05	1.00E+00	0.0000
ENA|CAJOCC010000403|CAJOCC010000403.1	autosomal	46.575	7.00E-05	7.00E-05	1.00E+00	0.0000
ENA|CAJOCC010000408|CAJOCC010000408.1	autosomal	17.804	3.00E-05	3.00E-05	1.00E+00	0.0000
ENA|CAJOCC010000426|CAJOCC010000426.1	autosomal	71.13	9.00E-05	9.00E-05	1.00E+00	0.0000
ENA|CAJOCC010000427|CAJOCC010000427.1	autosomal	35.014	4.00E-05	4.00E-05	1.00E+00	0.0000
ENA|CAJOCC010000428|CAJOCC010000428.1	autosomal	11.884	1.00E-05	1.00E-05	1.00E+00	0.0000
ENA|CAJOCC010000429|CAJOCC010000429.1	autosomal	46.226	6.00E-05	6.00E-05	1.00E+00	0.0000
ENA|CAJOCC010000436|CAJOCC010000436.1	autosomal	23.075	1.00E-05	1.00E-05	1.00E+00	0.0000

ENA|CAJOCC010000442|CAJOCC010000442.1	autosomal	17.67	1.00E-05	1.00E-05	1.00E+00	0.0000
ENA|CAJOCC010000447|CAJOCC010000447.1	autosomal	343.293	0.00043	0.00043	1.00E+00	0.0000
ENA|CAJOCC010000448|CAJOCC010000448.1	autosomal	49.906	1.00E-05	1.00E-05	1.00E+00	0.0000
ENA|CAJOCC010000450|CAJOCC010000450.1	autosomal	28.901	3.00E-05	3.00E-05	1.00E+00	0.0000
ENA|CAJOCC010000453|CAJOCC010000453.1	autosomal	27.058	2.00E-05	2.00E-05	1.00E+00	0.0000
ENA|CAJOCC010000455|CAJOCC010000455.1	autosomal	30.105	5.00E-05	5.00E-05	1.00E+00	0.0000
ENA|CAJOCC010000460|CAJOCC010000460.1	autosomal	43.665	3.00E-05	3.00E-05	1.00E+00	0.0000
ENA|CAJOCC010000461|CAJOCC010000461.1	autosomal	2.443	1.00E-05	1.00E-05	1.00E+00	0.0000
ENA|CAJOCC010000467|CAJOCC010000467.1	autosomal	378.263	5.00E-04	5.00E-04	1.00E+00	0.0000
ENA|CAJOCC010000471|CAJOCC010000471.1	autosomal	5.387	1.00E-05	1.00E-05	1.00E+00	0.0000
ENA|CAJOCC010000476|CAJOCC010000476.1	autosomal	21.566	2.00E-05	2.00E-05	1.00E+00	0.0000
ENA|CAJOCC010000477|CAJOCC010000477.1	autosomal	15.116	1.00E-05	1.00E-05	1.00E+00	0.0000
ENA|CAJOCC010000480|CAJOCC010000480.1	autosomal	17.383	3.00E-05	3.00E-05	1.00E+00	0.0000
ENA|CAJOCC010000481|CAJOCC010000481.1	autosomal	87.878	0.00011	0.00011	1.00E+00	0.0000
ENA|CAJOCC010000489|CAJOCC010000489.1	autosomal	19.531	2.00E-05	2.00E-05	1.00E+00	0.0000
ENA|CAJOCC010000494|CAJOCC010000494.1	autosomal	22.945	1.00E-05	1.00E-05	1.00E+00	0.0000
ENA|CAJOCC010000495|CAJOCC010000495.1	autosomal	1.241	2.00E-05	2.00E-05	1.00E+00	0.0000
ENA|CAJOCC010000499|CAJOCC010000499.1	autosomal	99.275	0.00012	0.00012	1.00E+00	0.0000
ENA|CAJOCC010000507|CAJOCC010000507.1	autosomal	28.368	2.00E-05	2.00E-05	1.00E+00	0.0000
ENA|CAJOCC010000513|CAJOCC010000513.1	autosomal	18.686	1.00E-05	1.00E-05	1.00E+00	0.0000
ENA|CAJOCC010000532|CAJOCC010000532.1	autosomal	5.862	1.00E-05	1.00E-05	1.00E+00	0.0000
ENA|CAJOCC010000534|CAJOCC010000534.1	autosomal	13.974	1.00E-05	1.00E-05	1.00E+00	0.0000
ENA|CAJOCC010000540|CAJOCC010000540.1	autosomal	15.203	2.00E-05	2.00E-05	1.00E+00	0.0000
ENA|CAJOCC010000548|CAJOCC010000548.1	autosomal	82.973	0.00014	0.00014	1.00E+00	0.0000
ENA|CAJOCC010000549|CAJOCC010000549.1	autosomal	7.059	3.00E-05	3.00E-05	1.00E+00	0.0000
ENA|CAJOCC010000550|CAJOCC010000550.1	autosomal	28.185	4.00E-05	4.00E-05	1.00E+00	0.0000
ENA|CAJOCC010000553|CAJOCC010000553.1	autosomal	6.498	3.00E-05	3.00E-05	1.00E+00	0.0000
ENA|CAJOCC010000566|CAJOCC010000566.1	autosomal	32.041	4.00E-05	4.00E-05	1.00E+00	0.0000
ENA|CAJOCC010000575|CAJOCC010000575.1	autosomal	44.049	5.00E-05	5.00E-05	1.00E+00	0.0000
ENA|CAJOCC010000578|CAJOCC010000578.1	autosomal	16.283	5.00E-05	5.00E-05	1.00E+00	0.0000
ENA|CAJOCC010000582|CAJOCC010000582.1	autosomal	16.705	2.00E-05	2.00E-05	1.00E+00	0.0000
ENA|CAJOCC010000596|CAJOCC010000596.1	autosomal	5.172	1.00E-05	1.00E-05	1.00E+00	0.0000

ENA|CAJOCC010000605|CAJOCC010000605.1	autosomal	21.203	1.00E-05	1.00E-05	1.00E+00	0.0000
ENA|CAJOCC010000620|CAJOCC010000620.1	autosomal	20.707	1.00E-05	1.00E-05	1.00E+00	0.0000
ENA|CAJOCC010000624|CAJOCC010000624.1	autosomal	7.601	2.00E-05	2.00E-05	1.00E+00	0.0000
ENA|CAJOCC010000626|CAJOCC010000626.1	autosomal	41.145	3.00E-04	3.00E-04	1.00E+00	0.0000
ENA|CAJOCC010000636|CAJOCC010000636.1	autosomal	17.99	1.00E-05	1.00E-05	1.00E+00	0.0000
ENA|CAJOCC010000641|CAJOCC010000641.1	autosomal	50.68	8.00E-05	8.00E-05	1.00E+00	0.0000
ENA|CAJOCC010000645|CAJOCC010000645.1	autosomal	4.688	1.00E-05	1.00E-05	1.00E+00	0.0000
ENA|CAJOCC010000649|CAJOCC010000649.1	autosomal	1.184	1.00E-05	1.00E-05	1.00E+00	0.0000
ENA|CAJOCC010000661|CAJOCC010000661.1	autosomal	102.937	1.00E-04	1.00E-04	1.00E+00	0.0000
ENA|CAJOCC010000665|CAJOCC010000665.1	autosomal	8.197	2.00E-05	2.00E-05	1.00E+00	0.0000
ENA|CAJOCC010000666|CAJOCC010000666.1	autosomal	127.155	1.00E-05	1.00E-05	1.00E+00	0.0000
ENA|CAJOCC010000681|CAJOCC010000681.1	autosomal	16.799	4.00E-05	4.00E-05	1.00E+00	0.0000
ENA|CAJOCC010000682|CAJOCC010000682.1	autosomal	35.304	2.00E-05	2.00E-05	1.00E+00	0.0000
ENA|CAJOCC010000687|CAJOCC010000687.1	autosomal	21.926	2.00E-05	2.00E-05	1.00E+00	0.0000
ENA|CAJOCC010000689|CAJOCC010000689.1	autosomal	2.278	1.00E-05	1.00E-05	1.00E+00	0.0000
ENA|CAJOCC010000697|CAJOCC010000697.1	autosomal	41.418	1.00E-05	1.00E-05	1.00E+00	0.0000
ENA|CAJOCC010000703|CAJOCC010000703.1	autosomal	23.324	2.00E-05	2.00E-05	1.00E+00	0.0000
ENA|CAJOCC010000705|CAJOCC010000705.1	autosomal	11.443	1.00E-05	1.00E-05	1.00E+00	0.0000
ENA|CAJOCC010000706|CAJOCC010000706.1	autosomal	9.108	3.00E-05	3.00E-05	1.00E+00	0.0000
ENA|CAJOCC010000710|CAJOCC010000710.1	autosomal	1.371	1.00E-05	1.00E-05	1.00E+00	0.0000
ENA|CAJOCC010000711|CAJOCC010000711.1	autosomal	83.38	0.00011	0.00011	1.00E+00	0.0000
ENA|CAJOCC010000724|CAJOCC010000724.1	autosomal	5.172	2.00E-05	2.00E-05	1.00E+00	0.0000
ENA|CAJOCC010000726|CAJOCC010000726.1	autosomal	45.846	7.00E-05	7.00E-05	1.00E+00	0.0000
ENA|CAJOCC010000727|CAJOCC010000727.1	autosomal	1.848	3.00E-05	3.00E-05	1.00E+00	0.0000
ENA|CAJOCC010000732|CAJOCC010000732.1	autosomal	40.38	2.00E-05	2.00E-05	1.00E+00	0.0000
ENA|CAJOCC010000745|CAJOCC010000745.1	autosomal	25.309	1.00E-05	1.00E-05	1.00E+00	0.0000
ENA|CAJOCC010000764|CAJOCC010000764.1	autosomal	7.224	1.00E-05	1.00E-05	1.00E+00	0.0000
ENA|CAJOCC010000770|CAJOCC010000770.1	autosomal	4.928	1.00E-05	1.00E-05	1.00E+00	0.0000
ENA|CAJOCC010000772|CAJOCC010000772.1	autosomal	11.961	1.00E-05	1.00E-05	1.00E+00	0.0000
ENA|CAJOCC010000798|CAJOCC010000798.1	autosomal	22.65	3.00E-05	3.00E-05	1.00E+00	0.0000
ENA|CAJOCC010000799|CAJOCC010000799.1	autosomal	40.4	3.00E-05	3.00E-05	1.00E+00	0.0000
ENA|CAJOCC010000802|CAJOCC010000802.1	autosomal	3.125	1.00E-05	1.00E-05	1.00E+00	0.0000

ENA|CAJOCC010000803|CAJOCC010000803.1	autosomal	2.078	1.00E-05	1.00E-05	1.00E+00	0.0000
ENA|CAJOCC010000804|CAJOCC010000804.1	autosomal	334.494	0.00043	0.00043	1.00E+00	0.0000
ENA|CAJOCC010000816|CAJOCC010000816.1	autosomal	42.329	3.00E-05	3.00E-05	1.00E+00	0.0000
ENA|CAJOCC010000820|CAJOCC010000820.1	autosomal	9.566	1.00E-05	1.00E-05	1.00E+00	0.0000
ENA|CAJOCC010000822|CAJOCC010000822.1	autosomal	34.06	4.00E-05	4.00E-05	1.00E+00	0.0000
ENA|CAJOCC010000832|CAJOCC010000832.1	autosomal	20.995	2.00E-05	2.00E-05	1.00E+00	0.0000
ENA|CAJOCC010000835|CAJOCC010000835.1	autosomal	16.689	1.00E-05	1.00E-05	1.00E+00	0.0000
ENA|CAJOCC010000836|CAJOCC010000836.1	autosomal	145.307	0.00021	0.00021	1.00E+00	0.0000
ENA|CAJOCC010000838|CAJOCC010000838.1	autosomal	5.201	2.00E-05	2.00E-05	1.00E+00	0.0000
ENA|CAJOCC010000856|CAJOCC010000856.1	autosomal	2.667	3.00E-05	3.00E-05	1.00E+00	0.0000
ENA|CAJOCC010000858|CAJOCC010000858.1	autosomal	9.605	1.00E-05	1.00E-05	1.00E+00	0.0000
ENA|CAJOCC010000865|CAJOCC010000865.1	autosomal	87.634	1.00E-05	1.00E-05	1.00E+00	0.0000
ENA|CAJOCC010000870|CAJOCC010000870.1	autosomal	22.559	2.00E-05	2.00E-05	1.00E+00	0.0000
ENA|CAJOCC010000873|CAJOCC010000873.1	autosomal	6.469	1.00E-05	1.00E-05	1.00E+00	0.0000
ENA|CAJOCC010000874|CAJOCC010000874.1	autosomal	20.609	1.00E-05	1.00E-05	1.00E+00	0.0000
ENA|CAJOCC010000876|CAJOCC010000876.1	autosomal	80.924	3.00E-05	3.00E-05	1.00E+00	0.0000
ENA|CAJOCC010000878|CAJOCC010000878.1	autosomal	14.165	2.00E-05	2.00E-05	1.00E+00	0.0000
ENA|CAJOCC010000894|CAJOCC010000894.1	autosomal	16.78	1.00E-05	1.00E-05	1.00E+00	0.0000
ENA|CAJOCC010000904|CAJOCC010000904.1	autosomal	10.058	1.00E-05	1.00E-05	1.00E+00	0.0000
ENA|CAJOCC010000908|CAJOCC010000908.1	autosomal	40.895	2.00E-05	2.00E-05	1.00E+00	0.0000
ENA|CAJOCC010000912|CAJOCC010000912.1	autosomal	38.595	2.00E-05	2.00E-05	1.00E+00	0.0000
ENA|CAJOCC010000914|CAJOCC010000914.1	autosomal	241.305	0.00032	0.00032	1.00E+00	0.0000
ENA|CAJOCC010000921|CAJOCC010000921.1	autosomal	16.308	1.00E-05	1.00E-05	1.00E+00	0.0000
ENA|CAJOCC010000931|CAJOCC010000931.1	autosomal	5.019	1.00E-05	1.00E-05	1.00E+00	0.0000
ENA|CAJOCC010000937|CAJOCC010000937.1	autosomal	126.726	0.00016	0.00016	1.00E+00	0.0000
ENA|CAJOCC010000943|CAJOCC010000943.1	autosomal	14.919	1.00E-05	1.00E-05	1.00E+00	0.0000
ENA|CAJOCC010000944|CAJOCC010000944.1	autosomal	40.988	1.00E-05	1.00E-05	1.00E+00	0.0000
ENA|CAJOCC010000949|CAJOCC010000949.1	autosomal	22.863	1.00E-05	1.00E-05	1.00E+00	0.0000
ENA|CAJOCC010000954|CAJOCC010000954.1	autosomal	1.088	5.00E-05	5.00E-05	1.00E+00	0.0000
ENA|CAJOCC010000957|CAJOCC010000957.1	autosomal	35.647	2.00E-05	2.00E-05	1.00E+00	0.0000
ENA|CAJOCC010000959|CAJOCC010000959.1	autosomal	20.579	5.00E-05	5.00E-05	1.00E+00	0.0000
ENA|CAJOCC010000962|CAJOCC010000962.1	autosomal	39.571	4.00E-05	4.00E-05	1.00E+00	0.0000

ENA|CAJOCC010000967|CAJOCC010000967.1	autosomal	6.709	2.00E-05	2.00E-05	1.00E+00	0.0000
ENA|CAJOCC010000984|CAJOCC010000984.1	autosomal	21.403	3.00E-05	3.00E-05	1.00E+00	0.0000
ENA|CAJOCC010000985|CAJOCC010000985.1	autosomal	7.572	5.00E-05	5.00E-05	1.00E+00	0.0000
ENA|CAJOCC010000991|CAJOCC010000991.1	autosomal	17.915	1.00E-05	1.00E-05	1.00E+00	0.0000
ENA|CAJOCC010001000|CAJOCC010001000.1	autosomal	36.853	3.00E-05	3.00E-05	1.00E+00	0.0000
ENA|CAJOCC010001010|CAJOCC010001010.1	autosomal	34.929	1.00E-05	1.00E-05	1.00E+00	0.0000
ENA|CAJOCC010001023|CAJOCC010001023.1	autosomal	13.253	1.00E-05	1.00E-05	1.00E+00	0.0000
ENA|CAJOCC010001024|CAJOCC010001024.1	autosomal	8.349	3.00E-05	3.00E-05	1.00E+00	0.0000
ENA|CAJOCC010001042|CAJOCC010001042.1	autosomal	3.077	1.00E-05	1.00E-05	1.00E+00	0.0000
ENA|CAJOCC010001062|CAJOCC010001062.1	autosomal	3.976	2.00E-05	2.00E-05	1.00E+00	0.0000
ENA|CAJOCC010001068|CAJOCC010001068.1	autosomal	8.706	1.00E-05	1.00E-05	1.00E+00	0.0000
ENA|CAJOCC010001075|CAJOCC010001075.1	autosomal	7.297	2.00E-05	2.00E-05	1.00E+00	0.0000
ENA|CAJOCC010001079|CAJOCC010001079.1	autosomal	121.203	0.00017	0.00017	1.00E+00	0.0000
ENA|CAJOCC010001083|CAJOCC010001083.1	autosomal	22.596	1.00E-05	1.00E-05	1.00E+00	0.0000
ENA|CAJOCC010001086|CAJOCC010001086.1	autosomal	4.741	1.00E-05	1.00E-05	1.00E+00	0.0000
ENA|CAJOCC010001088|CAJOCC010001088.1	autosomal	16.642	1.00E-05	1.00E-05	1.00E+00	0.0000
ENA|CAJOCC010001093|CAJOCC010001093.1	autosomal	15.925	2.00E-05	2.00E-05	1.00E+00	0.0000
ENA|CAJOCC010001094|CAJOCC010001094.1	autosomal	9.063	1.00E-05	1.00E-05	1.00E+00	0.0000
ENA|CAJOCC010001096|CAJOCC010001096.1	autosomal	32.218	5.00E-05	5.00E-05	1.00E+00	0.0000
ENA|CAJOCC010001104|CAJOCC010001104.1	autosomal	15.329	1.00E-05	1.00E-05	1.00E+00	0.0000
ENA|CAJOCC010001113|CAJOCC010001113.1	autosomal	56.336	7.00E-05	7.00E-05	1.00E+00	0.0000
ENA|CAJOCC010001118|CAJOCC010001118.1	autosomal	11.374	1.00E-05	1.00E-05	1.00E+00	0.0000
ENA|CAJOCC010001121|CAJOCC010001121.1	autosomal	32.224	1.00E-05	1.00E-05	1.00E+00	0.0000
ENA|CAJOCC010001127|CAJOCC010001127.1	autosomal	7.768	1.00E-05	1.00E-05	1.00E+00	0.0000
ENA|CAJOCC010001136|CAJOCC010001136.1	autosomal	68.023	2.00E-05	2.00E-05	1.00E+00	0.0000
ENA|CAJOCC010001138|CAJOCC010001138.1	autosomal	9.281	2.00E-05	2.00E-05	1.00E+00	0.0000
ENA|CAJOCC010001139|CAJOCC010001139.1	autosomal	16.318	1.00E-05	1.00E-05	1.00E+00	0.0000
ENA|CAJOCC010001151|CAJOCC010001151.1	autosomal	18.361	2.00E-05	2.00E-05	1.00E+00	0.0000
ENA|CAJOCC010001155|CAJOCC010001155.1	autosomal	133.831	0.00018	0.00018	1.00E+00	0.0000
ENA|CAJOCC010001171|CAJOCC010001171.1	autosomal	31.827	3.00E-05	3.00E-05	1.00E+00	0.0000
ENA|CAJOCC010001172|CAJOCC010001172.1	autosomal	34.536	2.00E-05	2.00E-05	1.00E+00	0.0000
ENA|CAJOCC010001183|CAJOCC010001183.1	autosomal	12.268	1.00E-05	1.00E-05	1.00E+00	0.0000

ENA|CAJOCC010001184|CAJOCC010001184.1	autosomal	873.858	0.00123	0.00123	1.00E+00	0.0000
ENA|CAJOCC010001185|CAJOCC010001185.1	autosomal	14.541	1.00E-05	1.00E-05	1.00E+00	0.0000
ENA|CAJOCC010001191|CAJOCC010001191.1	autosomal	20.338	6.00E-05	6.00E-05	1.00E+00	0.0000
ENA|CAJOCC010001193|CAJOCC010001193.1	autosomal	84.204	7.00E-05	7.00E-05	1.00E+00	0.0000
ENA|CAJOCC010001195|CAJOCC010001195.1	autosomal	6.68	1.00E-05	1.00E-05	1.00E+00	0.0000
ENA|CAJOCC010001200|CAJOCC010001200.1	autosomal	48.624	1.00E-05	1.00E-05	1.00E+00	0.0000
ENA|CAJOCC010001201|CAJOCC010001201.1	autosomal	0.687	3.00E-05	3.00E-05	1.00E+00	0.0000
ENA|CAJOCC010001212|CAJOCC010001212.1	autosomal	18.03	1.00E-05	1.00E-05	1.00E+00	0.0000
ENA|CAJOCC010001213|CAJOCC010001213.1	autosomal	14.411	3.00E-05	3.00E-05	1.00E+00	0.0000
ENA|CAJOCC010001215|CAJOCC010001215.1	autosomal	15.681	1.00E-05	1.00E-05	1.00E+00	0.0000
ENA|CAJOCC010001217|CAJOCC010001217.1	autosomal	7.659	1.00E-05	1.00E-05	1.00E+00	0.0000
ENA|CAJOCC010001222|CAJOCC010001222.1	autosomal	16.004	1.00E-05	1.00E-05	1.00E+00	0.0000
ENA|CAJOCC010001234|CAJOCC010001234.1	autosomal	499.997	0.00063	0.00063	1.00E+00	0.0000
ENA|CAJOCC010001236|CAJOCC010001236.1	autosomal	6.895	1.00E-05	1.00E-05	1.00E+00	0.0000
ENA|CAJOCC010001241|CAJOCC010001241.1	autosomal	11.493	1.00E-05	1.00E-05	1.00E+00	0.0000
ENA|CAJOCC010001244|CAJOCC010001244.1	autosomal	25.521	1.00E-05	1.00E-05	1.00E+00	0.0000
ENA|CAJOCC010001248|CAJOCC010001248.1	autosomal	23.474	3.00E-05	3.00E-05	1.00E+00	0.0000
ENA|CAJOCC010001252|CAJOCC010001252.1	autosomal	0.528	2.00E-05	2.00E-05	1.00E+00	0.0000
ENA|CAJOCC010001260|CAJOCC010001260.1	autosomal	5.532	1.00E-05	1.00E-05	1.00E+00	0.0000
ENA|CAJOCC010001273|CAJOCC010001273.1	autosomal	3.289	1.00E-05	1.00E-05	1.00E+00	0.0000
ENA|CAJOCC010001281|CAJOCC010001281.1	autosomal	25.478	4.00E-05	4.00E-05	1.00E+00	0.0000
ENA|CAJOCC010001286|CAJOCC010001286.1	autosomal	2.111	1.00E-05	1.00E-05	1.00E+00	0.0000
ENA|CAJOCC010001293|CAJOCC010001293.1	autosomal	40.964	4.00E-05	4.00E-05	1.00E+00	0.0000
ENA|CAJOCC010001298|CAJOCC010001298.1	autosomal	5.713	2.00E-05	2.00E-05	1.00E+00	0.0000
ENA|CAJOCC010001303|CAJOCC010001303.1	autosomal	16.183	1.00E-05	1.00E-05	1.00E+00	0.0000
ENA|CAJOCC010001305|CAJOCC010001305.1	autosomal	2.478	3.00E-05	3.00E-05	1.00E+00	0.0000
ENA|CAJOCC010001306|CAJOCC010001306.1	autosomal	1.857	1.00E-05	1.00E-05	1.00E+00	0.0000
ENA|CAJOCC010001308|CAJOCC010001308.1	autosomal	36.873	1.00E-05	1.00E-05	1.00E+00	0.0000
ENA|CAJOCC010001311|CAJOCC010001311.1	autosomal	15.163	1.00E-05	1.00E-05	1.00E+00	0.0000
ENA|CAJOCC010001316|CAJOCC010001316.1	autosomal	14.041	2.00E-05	2.00E-05	1.00E+00	0.0000
ENA|CAJOCC010001320|CAJOCC010001320.1	autosomal	16.639	1.00E-05	1.00E-05	1.00E+00	0.0000
ENA|CAJOCC010001323|CAJOCC010001323.1	autosomal	6.091	1.00E-05	1.00E-05	1.00E+00	0.0000

ENA|CAJOCC010001330|CAJOCC010001330.1	autosomal	31.001	2.00E-05	2.00E-05	1.00E+00	0.0000
ENA|CAJOCC010001341|CAJOCC010001341.1	autosomal	188.65	3.00E-04	3.00E-04	1.00E+00	0.0000
ENA|CAJOCC010001342|CAJOCC010001342.1	autosomal	12.577	1.00E-05	1.00E-05	1.00E+00	0.0000
ENA|CAJOCC010001344|CAJOCC010001344.1	autosomal	6.257	1.00E-05	1.00E-05	1.00E+00	0.0000
ENA|CAJOCC010001349|CAJOCC010001349.1	autosomal	79.309	6.00E-05	6.00E-05	1.00E+00	0.0000
ENA|CAJOCC010001356|CAJOCC010001356.1	autosomal	15.114	3.00E-05	3.00E-05	1.00E+00	0.0000
ENA|CAJOCC010001360|CAJOCC010001360.1	autosomal	51.788	5.00E-05	5.00E-05	1.00E+00	0.0000
ENA|CAJOCC010001363|CAJOCC010001363.1	autosomal	22.427	3.00E-05	3.00E-05	1.00E+00	0.0000
ENA|CAJOCC010001366|CAJOCC010001366.1	autosomal	9.476	1.00E-05	1.00E-05	1.00E+00	0.0000
ENA|CAJOCC010001367|CAJOCC010001367.1	autosomal	18.339	2.00E-05	2.00E-05	1.00E+00	0.0000
ENA|CAJOCC010001368|CAJOCC010001368.1	autosomal	6.191	3.00E-05	3.00E-05	1.00E+00	0.0000
ENA|CAJOCC010001377|CAJOCC010001377.1	autosomal	67.32	0.00011	0.00011	1.00E+00	0.0000
ENA|CAJOCC010001384|CAJOCC010001384.1	autosomal	15.094	1.00E-05	1.00E-05	1.00E+00	0.0000
ENA|CAJOCC010001387|CAJOCC010001387.1	autosomal	24.817	5.00E-05	5.00E-05	1.00E+00	0.0000
ENA|CAJOCC010001391|CAJOCC010001391.1	autosomal	59.584	4.00E-05	4.00E-05	1.00E+00	0.0000
ENA|CAJOCC010001397|CAJOCC010001397.1	autosomal	68.557	0.00012	0.00012	1.00E+00	0.0000
ENA|CAJOCC010001410|CAJOCC010001410.1	autosomal	45.953	5.00E-05	5.00E-05	1.00E+00	0.0000
ENA|CAJOCC010001411|CAJOCC010001411.1	autosomal	68.136	8.00E-05	8.00E-05	1.00E+00	0.0000
ENA|CAJOCC010001412|CAJOCC010001412.1	autosomal	48.804	3.00E-05	3.00E-05	1.00E+00	0.0000
ENA|CAJOCC010001413|CAJOCC010001413.1	autosomal	10.957	1.00E-05	1.00E-05	1.00E+00	0.0000
ENA|CAJOCC010001418|CAJOCC010001418.1	autosomal	22.249	3.00E-05	3.00E-05	1.00E+00	0.0000
ENA|CAJOCC010001421|CAJOCC010001421.1	autosomal	17.579	2.00E-05	2.00E-05	1.00E+00	0.0000
ENA|CAJOCC010001434|CAJOCC010001434.1	autosomal	97.551	4.00E-05	4.00E-05	1.00E+00	0.0000
ENA|CAJOCC010001438|CAJOCC010001438.1	autosomal	36.785	2.00E-05	2.00E-05	1.00E+00	0.0000
ENA|CAJOCC010001442|CAJOCC010001442.1	autosomal	23.303	3.00E-05	3.00E-05	1.00E+00	0.0000
ENA|CAJOCC010001443|CAJOCC010001443.1	autosomal	42.4	2.00E-05	2.00E-05	1.00E+00	0.0000
ENA|CAJOCC010001448|CAJOCC010001448.1	autosomal	11.671	1.00E-05	1.00E-05	1.00E+00	0.0000
ENA|CAJOCC010001449|CAJOCC010001449.1	autosomal	158.356	0.00021	0.00021	1.00E+00	0.0000
ENA|CAJOCC010001450|CAJOCC010001450.1	autosomal	29.258	4.00E-05	4.00E-05	1.00E+00	0.0000
ENA|CAJOCC010001451|CAJOCC010001451.1	autosomal	9.125	1.00E-05	1.00E-05	1.00E+00	0.0000
ENA|CAJOCC010001452|CAJOCC010001452.1	autosomal	81.39	0.00013	0.00013	1.00E+00	0.0000
ENA|CAJOCC010001456|CAJOCC010001456.1	autosomal	34.293	4.00E-05	4.00E-05	1.00E+00	0.0000

ENA|CAJOCC010001468|CAJOCC010001468.1	autosomal	63.654	0.00011	0.00011	1.00E+00	0.0000
ENA|CAJOCC010001469|CAJOCC010001469.1	autosomal	11.139	1.00E-05	1.00E-05	1.00E+00	0.0000
ENA|CAJOCC010001473|CAJOCC010001473.1	autosomal	29.816	2.00E-05	2.00E-05	1.00E+00	0.0000
ENA|CAJOCC010001478|CAJOCC010001478.1	autosomal	59.843	2.00E-05	2.00E-05	1.00E+00	0.0000
ENA|CAJOCC010001479|CAJOCC010001479.1	autosomal	12.109	1.00E-05	1.00E-05	1.00E+00	0.0000
ENA|CAJOCC010001480|CAJOCC010001480.1	autosomal	14.598	7.00E-05	7.00E-05	1.00E+00	0.0000
ENA|CAJOCC010001482|CAJOCC010001482.1	autosomal	6.687	5.00E-05	5.00E-05	1.00E+00	0.0000
ENA|CAJOCC010001483|CAJOCC010001483.1	autosomal	37.986	2.00E-05	2.00E-05	1.00E+00	0.0000
ENA|CAJOCC010001486|CAJOCC010001486.1	autosomal	16.211	1.00E-05	1.00E-05	1.00E+00	0.0000
ENA|CAJOCC010001487|CAJOCC010001487.1	autosomal	7.423	1.00E-05	1.00E-05	1.00E+00	0.0000
ENA|CAJOCC010001494|CAJOCC010001494.1	autosomal	195.476	0.00023	0.00023	1.00E+00	0.0000
ENA|CAJOCC010001495|CAJOCC010001495.1	autosomal	15.427	1.00E-05	1.00E-05	1.00E+00	0.0000
ENA|CAJOCC010001496|CAJOCC010001496.1	autosomal	11.353	1.00E-05	1.00E-05	1.00E+00	0.0000
ENA|CAJOCC010001498|CAJOCC010001498.1	autosomal	10.021	1.00E-05	1.00E-05	1.00E+00	0.0000
ENA|CAJOCC010001503|CAJOCC010001503.1	autosomal	43.997	1.00E-05	1.00E-05	1.00E+00	0.0000
ENA|CAJOCC010001515|CAJOCC010001515.1	autosomal	16.709	1.00E-05	1.00E-05	1.00E+00	0.0000
ENA|CAJOCC010001523|CAJOCC010001523.1	autosomal	16.248	1.00E-05	1.00E-05	1.00E+00	0.0000
ENA|CAJOCC010001525|CAJOCC010001525.1	autosomal	315.332	0.00042	0.00042	1.00E+00	0.0000
ENA|CAJOCC010001529|CAJOCC010001529.1	autosomal	27.182	1.00E-05	1.00E-05	1.00E+00	0.0000
ENA|CAJOCC010001536|CAJOCC010001536.1	autosomal	9.1	2.00E-05	2.00E-05	1.00E+00	0.0000
ENA|CAJOCC010001537|CAJOCC010001537.1	autosomal	74.23	9.00E-05	9.00E-05	1.00E+00	0.0000
ENA|CAJOCC010001539|CAJOCC010001539.1	autosomal	27.454	2.00E-05	2.00E-05	1.00E+00	0.0000
ENA|CAJOCC010001543|CAJOCC010001543.1	autosomal	41.167	2.00E-05	2.00E-05	1.00E+00	0.0000
ENA|CAJOCC010001545|CAJOCC010001545.1	autosomal	37.634	0.00019	0.00019	1.00E+00	0.0000
ENA|CAJOCC010001548|CAJOCC010001548.1	autosomal	171.103	0.00017	0.00017	1.00E+00	0.0000
ENA|CAJOCC010001549|CAJOCC010001549.1	autosomal	17.759	1.00E-05	1.00E-05	1.00E+00	0.0000
ENA|CAJOCC010001550|CAJOCC010001550.1	autosomal	15.48	1.00E-05	1.00E-05	1.00E+00	0.0000
ENA|CAJOCC010001553|CAJOCC010001553.1	autosomal	30.236	2.00E-05	2.00E-05	1.00E+00	0.0000
ENA|CAJOCC010001558|CAJOCC010001558.1	autosomal	11.871	0.00011	0.00011	1.00E+00	0.0000
ENA|CAJOCC010001559|CAJOCC010001559.1	autosomal	5.943	1.00E-05	1.00E-05	1.00E+00	0.0000
ENA|CAJOCC010001571|CAJOCC010001571.1	autosomal	54.104	2.00E-05	2.00E-05	1.00E+00	0.0000
ENA|CAJOCC010001585|CAJOCC010001585.1	autosomal	40.534	1.00E-05	1.00E-05	1.00E+00	0.0000

ENA|CAJOCC010001589|CAJOCC010001589.1	autosomal	479.42	0.00064	0.00064	1.00E+00	0.0000
ENA|CAJOCC010001600|CAJOCC010001600.1	autosomal	14.451	1.00E-05	1.00E-05	1.00E+00	0.0000
ENA|CAJOCC010001606|CAJOCC010001606.1	autosomal	45.216	3.00E-05	3.00E-05	1.00E+00	0.0000
ENA|CAJOCC010001611|CAJOCC010001611.1	autosomal	14.466	1.00E-05	1.00E-05	1.00E+00	0.0000
ENA|CAJOCC010001612|CAJOCC010001612.1	autosomal	8.13	1.00E-05	1.00E-05	1.00E+00	0.0000
ENA|CAJOCC010001613|CAJOCC010001613.1	autosomal	23.43	2.00E-05	2.00E-05	1.00E+00	0.0000
ENA|CAJOCC010001616|CAJOCC010001616.1	autosomal	90.112	0.00022	0.00022	1.00E+00	0.0000
ENA|CAJOCC010001620|CAJOCC010001620.1	autosomal	26.823	4.00E-05	4.00E-05	1.00E+00	0.0000
ENA|CAJOCC010001626|CAJOCC010001626.1	autosomal	23.22	1.00E-05	1.00E-05	1.00E+00	0.0000
ENA|CAJOCC010001627|CAJOCC010001627.1	autosomal	18.503	5.00E-05	5.00E-05	1.00E+00	0.0000
ENA|CAJOCC010001628|CAJOCC010001628.1	autosomal	12.917	1.00E-05	1.00E-05	1.00E+00	0.0000
ENA|CAJOCC010001629|CAJOCC010001629.1	autosomal	11.247	1.00E-05	1.00E-05	1.00E+00	0.0000
ENA|CAJOCC010001634|CAJOCC010001634.1	autosomal	24.184	3.00E-05	3.00E-05	1.00E+00	0.0000
ENA|CAJOCC010001640|CAJOCC010001640.1	autosomal	17.141	2.00E-05	2.00E-05	1.00E+00	0.0000
ENA|CAJOCC010001641|CAJOCC010001641.1	autosomal	22.856	1.00E-05	1.00E-05	1.00E+00	0.0000
ENA|CAJOCC010001651|CAJOCC010001651.1	autosomal	74.073	0.00012	0.00012	1.00E+00	0.0000
ENA|CAJOCC010001653|CAJOCC010001653.1	autosomal	16.327	3.00E-05	3.00E-05	1.00E+00	0.0000
ENA|CAJOCC010001654|CAJOCC010001654.1	autosomal	9.349	1.00E-05	1.00E-05	1.00E+00	0.0000
ENA|CAJOCC010001665|CAJOCC010001665.1	autosomal	1844.195	0.00246	0.00246	1.00E+00	0.0000
ENA|CAJOCC010001673|CAJOCC010001673.1	autosomal	81.939	0.00012	0.00012	1.00E+00	0.0000
ENA|CAJOCC010001677|CAJOCC010001677.1	autosomal	122.429	0.00013	0.00013	1.00E+00	0.0000
ENA|CAJOCC010001679|CAJOCC010001679.1	autosomal	635.329	0.00082	0.00082	1.00E+00	0.0000
ENA|CAJOCC010001685|CAJOCC010001685.1	autosomal	38.044	4.00E-05	4.00E-05	1.00E+00	0.0000
ENA|CAJOCC010001686|CAJOCC010001686.1	autosomal	38.798	6.00E-05	6.00E-05	1.00E+00	0.0000
ENA|CAJOCC010001693|CAJOCC010001693.1	autosomal	25.806	4.00E-05	4.00E-05	1.00E+00	0.0000
ENA|CAJOCC010001702|CAJOCC010001702.1	autosomal	1404.348	0.00193	0.00193	1.00E+00	0.0000
ENA|CAJOCC010001703|CAJOCC010001703.1	autosomal	13.393	1.00E-05	1.00E-05	1.00E+00	0.0000
ENA|CAJOCC010001707|CAJOCC010001707.1	autosomal	9.097	1.00E-05	1.00E-05	1.00E+00	0.0000
ENA|CAJOCC010001708|CAJOCC010001708.1	autosomal	15.836	2.00E-05	2.00E-05	1.00E+00	0.0000
ENA|CAJOCC010001716|CAJOCC010001716.1	autosomal	7.593	5.00E-05	5.00E-05	1.00E+00	0.0000
ENA|CAJOCC010001721|CAJOCC010001721.1	autosomal	35.579	4.00E-05	4.00E-05	1.00E+00	0.0000
ENA|CAJOCC010001731|CAJOCC010001731.1	autosomal	25.338	1.00E-05	1.00E-05	1.00E+00	0.0000

ENA|CAJOCC010001734|CAJOCC010001734.1	autosomal	58.681	2.00E-05	2.00E-05	1.00E+00	0.0000
ENA|CAJOCC010001738|CAJOCC010001738.1	autosomal	93.605	1.00E-04	1.00E-04	1.00E+00	0.0000
ENA|CAJOCC010001748|CAJOCC010001748.1	autosomal	71.23	2.00E-05	2.00E-05	1.00E+00	0.0000
ENA|CAJOCC010001750|CAJOCC010001750.1	autosomal	179.409	0.00024	0.00024	1.00E+00	0.0000
ENA|CAJOCC010001752|CAJOCC010001752.1	autosomal	622.749	9.00E-04	9.00E-04	1.00E+00	0.0000
ENA|CAJOCC010001753|CAJOCC010001753.1	autosomal	24.748	2.00E-05	2.00E-05	1.00E+00	0.0000
ENA|CAJOCC010001754|CAJOCC010001754.1	autosomal	57.198	3.00E-05	3.00E-05	1.00E+00	0.0000
ENA|CAJOCC010001756|CAJOCC010001756.1	autosomal	69.645	3.00E-05	3.00E-05	1.00E+00	0.0000
ENA|CAJOCC010001763|CAJOCC010001763.1	autosomal	445.503	0.00062	0.00062	1.00E+00	0.0000
ENA|CAJOCC010001764|CAJOCC010001764.1	autosomal	308.158	0.00048	0.00048	1.00E+00	0.0000
ENA|CAJOCC010001772|CAJOCC010001772.1	autosomal	101.943	0.00013	0.00013	1.00E+00	0.0000
ENA|CAJOCC010001776|CAJOCC010001776.1	autosomal	103.094	4.00E-05	4.00E-05	1.00E+00	0.0000
ENA|CAJOCC010001777|CAJOCC010001777.1	autosomal	10.833	3.00E-05	3.00E-05	1.00E+00	0.0000
ENA|CAJOCC010001780|CAJOCC010001780.1	autosomal	15.349	2.00E-05	2.00E-05	1.00E+00	0.0000
ENA|CAJOCC010001782|CAJOCC010001782.1	autosomal	298.247	0.00039	0.00039	1.00E+00	0.0000
ENA|CAJOCC010001790|CAJOCC010001790.1	autosomal	20.601	1.00E-05	1.00E-05	1.00E+00	0.0000
ENA|CAJOCC010001793|CAJOCC010001793.1	autosomal	32.392	5.00E-05	5.00E-05	1.00E+00	0.0000
ENA|CAJOCC010001794|CAJOCC010001794.1	autosomal	26.926	2.00E-05	2.00E-05	1.00E+00	0.0000
ENA|CAJOCC010001799|CAJOCC010001799.1	autosomal	23.35	1.00E-05	1.00E-05	1.00E+00	0.0000
ENA|CAJOCC010001801|CAJOCC010001801.1	autosomal	1035.784	0.00155	0.00155	1.00E+00	0.0000
ENA|CAJOCC010001803|CAJOCC010001803.1	autosomal	13.523	1.00E-05	1.00E-05	1.00E+00	0.0000
ENA|CAJOCC010001804|CAJOCC010001804.1	autosomal	16.661	1.00E-05	1.00E-05	1.00E+00	0.0000
ENA|CAJOCC010001809|CAJOCC010001809.1	autosomal	804.363	0.0011	0.0011	1.00E+00	0.0000
ENA|CAJOCC010001820|CAJOCC010001820.1	autosomal	24.193	2.00E-05	2.00E-05	1.00E+00	0.0000
ENA|CAJOCC010001824|CAJOCC010001824.1	autosomal	63.387	5.00E-05	5.00E-05	1.00E+00	0.0000
ENA|CAJOCC010001844|CAJOCC010001844.1	autosomal	70.329	1.00E-04	1.00E-04	1.00E+00	0.0000
ENA|CAJOCC010001854|CAJOCC010001854.1	autosomal	18.379	2.00E-05	2.00E-05	1.00E+00	0.0000
ENA|CAJOCC010001866|CAJOCC010001866.1	autosomal	8.646	2.00E-05	2.00E-05	1.00E+00	0.0000
ENA|CAJOCC010001868|CAJOCC010001868.1	autosomal	35.307	2.00E-05	2.00E-05	1.00E+00	0.0000
ENA|CAJOCC010001896|CAJOCC010001896.1	autosomal	9.859	1.00E-05	1.00E-05	1.00E+00	0.0000
ENA|CAJOCC010001904|CAJOCC010001904.1	autosomal	624.314	9.00E-04	9.00E-04	1.00E+00	0.0000
ENA|CAJOCC010001905|CAJOCC010001905.1	autosomal	21.593	1.00E-05	1.00E-05	1.00E+00	0.0000

ENA|CAJOCC010001906|CAJOCC010001906.1	autosomal	4109.572	0.00563	0.00563	1.00E+00	0.0000
ENA|CAJOCC010001917|CAJOCC010001917.1	autosomal	21.561	3.00E-05	3.00E-05	1.00E+00	0.0000
ENA|CAJOCC010001937|CAJOCC010001937.1	autosomal	3441.071	0.00437	0.00437	1.00E+00	0.0000
ENA|CAJOCC010001940|CAJOCC010001940.1	autosomal	546.92	0.00078	0.00078	1.00E+00	0.0000
ENA|CAJOCC010001743|CAJOCC010001743.1	autosomal	2251.459	0.00296	0.00295	1.00E+00	0.0049
ENA|CAJOCC010001382|CAJOCC010001382.1	autosomal	1427.975	0.00206	0.00205	1.00E+00	0.0070
ENA|CAJOCC010001927|CAJOCC010001927.1	autosomal	1437.41	0.00204	0.00203	1.00E+00	0.0071
ENA|CAJOCC010001909|CAJOCC010001909.1	autosomal	1070.613	0.00159	0.00158	1.01E+00	0.0091
ENA|CAJOCC010001781|CAJOCC010001781.1	autosomal	2312.949	0.00317	0.00315	1.01E+00	0.0091
ENA|CAJOCC010001885|CAJOCC010001885.1	autosomal	6425.179	0.00876	0.0087	1.01E+00	0.0099
ENA|CAJOCC010001610|CAJOCC010001610.1	autosomal	895.625	0.00126	0.00125	1.01E+00	0.0115
ENA|CAJOCC010000086|CAJOCC010000086.1	autosomal	3431.874	0.00477	0.00473	1.01E+00	0.0121
ENA|CAJOCC010001880|CAJOCC010001880.1	autosomal	1623.89	0.00216	0.00214	1.01E+00	0.0134
ENA|CAJOCC010001561|CAJOCC010001561.1	autosomal	2550.993	0.00317	0.00314	1.01E+00	0.0137
ENA|CAJOCC010001826|CAJOCC010001826.1	autosomal	734.437	0.00104	0.00103	1.01E+00	0.0139
ENA|CAJOCC010001742|CAJOCC010001742.1	autosomal	2855.481	0.004	0.00396	1.01E+00	0.0145
ENA|CAJOCC010001876|CAJOCC010001876.1	autosomal	2933.963	0.00368	0.00364	1.01E+00	0.0158
ENA|CAJOCC010000861|CAJOCC010000861.1	autosomal	607.571	0.00087	0.00086	1.01E+00	0.0167
ENA|CAJOCC010000503|CAJOCC010000503.1	autosomal	598.587	0.00086	0.00085	1.01E+00	0.0169
ENA|CAJOCC010001018|CAJOCC010001018.1	autosomal	610.203	0.00079	0.00078	1.01E+00	0.0184
ENA|CAJOCC010001729|CAJOCC010001729.1	autosomal	653.739	0.00077	0.00076	1.01E+00	0.0189
ENA|CAJOCC010001730|CAJOCC010001730.1	autosomal	1163.694	0.00151	0.00149	1.01E+00	0.0192
ENA|CAJOCC010001823|CAJOCC010001823.1	autosomal	523.514	0.00074	0.00073	1.01E+00	0.0196
ENA|CAJOCC010001706|CAJOCC010001706.1	autosomal	5632.433	0.00781	0.0077	1.01E+00	0.0205
ENA|CAJOCC010000136|CAJOCC010000136.1	autosomal	1005.162	0.00137	0.00135	1.01E+00	0.0212
ENA|CAJOCC010001928|CAJOCC010001928.1	autosomal	11290.493	0.01542	0.01519	1.02E+00	0.0217
ENA|CAJOCC010001160|CAJOCC010001160.1	autosomal	486.998	0.00067	0.00066	1.02E+00	0.0217
ENA|CAJOCC010000313|CAJOCC010000313.1	autosomal	4573.195	0.00661	0.00651	1.02E+00	0.0220
ENA|CAJOCC010001835|CAJOCC010001835.1	autosomal	928.188	0.00132	0.0013	1.02E+00	0.0220
ENA|CAJOCC010001736|CAJOCC010001736.1	autosomal	900.808	0.00123	0.00121	1.02E+00	0.0237
ENA|CAJOCC010001718|CAJOCC010001718.1	autosomal	403.431	6.00E-04	0.00059	1.02E+00	0.0242
ENA|CAJOCC010000843|CAJOCC010000843.1	autosomal	1297.312	0.00175	0.00172	1.02E+00	0.0249

ENA|CAJOCC010001614|CAJOCC010001614.1	autosomal	2024.438	0.00286	0.00281	1.02E+00	0.0254
ENA|CAJOCC010001848|CAJOCC010001848.1	autosomal	431.409	0.00057	0.00056	1.02E+00	0.0255
ENA|CAJOCC010001944|CAJOCC010001944.1	autosomal	2642.293	0.00391	0.00384	1.02E+00	0.0261
ENA|CAJOCC010001759|CAJOCC010001759.1	autosomal	2486.354	0.00333	0.00327	1.02E+00	0.0262
ENA|CAJOCC010000235|CAJOCC010000235.1	autosomal	4062.897	0.00553	0.00543	1.02E+00	0.0263
ENA|CAJOCC010001840|CAJOCC010001840.1	autosomal	1595.198	0.00221	0.00217	1.02E+00	0.0264
ENA|CAJOCC010000236|CAJOCC010000236.1	autosomal	435.143	0.00054	0.00053	1.02E+00	0.0270
ENA|CAJOCC010001373|CAJOCC010001373.1	autosomal	1685.028	0.00214	0.0021	1.02E+00	0.0272
ENA|CAJOCC010001011|CAJOCC010001011.1	autosomal	1626.922	0.00211	0.00207	1.02E+00	0.0276
ENA|CAJOCC010000124|CAJOCC010000124.1	autosomal	2852.184	0.00364	0.00357	1.02E+00	0.0280
ENA|CAJOCC010000250|CAJOCC010000250.1	autosomal	4591.042	0.00623	0.00611	1.02E+00	0.0281
ENA|CAJOCC010001625|CAJOCC010001625.1	autosomal	6987.528	0.01003	0.00983	1.02E+00	0.0291
ENA|CAJOCC010001903|CAJOCC010001903.1	autosomal	7223.03	0.00977	0.00957	1.02E+00	0.0298
ENA|CAJOCC010000773|CAJOCC010000773.1	autosomal	7198.922	0.00976	0.00956	1.02E+00	0.0299
ENA|CAJOCC010001855|CAJOCC010001855.1	autosomal	2805.253	0.0039	0.00382	1.02E+00	0.0299
ENA|CAJOCC010000012|CAJOCC010000012.1	autosomal	1020.203	0.00142	0.00139	1.02E+00	0.0308
ENA|CAJOCC010000016|CAJOCC010000016.1	autosomal	2970.83	0.00376	0.00368	1.02E+00	0.0310
ENA|CAJOCC010001884|CAJOCC010001884.1	autosomal	836.361	0.00094	0.00092	1.02E+00	0.0310
ENA|CAJOCC010000961|CAJOCC010000961.1	autosomal	2082.716	0.00281	0.00275	1.02E+00	0.0311
ENA|CAJOCC010001683|CAJOCC010001683.1	autosomal	1498.561	0.00187	0.00183	1.02E+00	0.0312
ENA|CAJOCC010001249|CAJOCC010001249.1	autosomal	4773.404	0.00606	0.00593	1.02E+00	0.0313
ENA|CAJOCC010000674|CAJOCC010000674.1	autosomal	4171.595	0.00558	0.00546	1.02E+00	0.0314
ENA|CAJOCC010000094|CAJOCC010000094.1	autosomal	1958.153	0.00277	0.00271	1.02E+00	0.0316
ENA|CAJOCC010001624|CAJOCC010001624.1	autosomal	1380.691	0.00184	0.0018	1.02E+00	0.0317
ENA|CAJOCC010001652|CAJOCC010001652.1	autosomal	4347.094	0.00588	0.00575	1.02E+00	0.0323
ENA|CAJOCC010000271|CAJOCC010000271.1	autosomal	383.114	0.00045	0.00044	1.02E+00	0.0324
ENA|CAJOCC010001638|CAJOCC010001638.1	autosomal	701.566	9.00E-04	0.00088	1.02E+00	0.0324
ENA|CAJOCC010001837|CAJOCC010001837.1	autosomal	343.333	0.00045	0.00044	1.02E+00	0.0324
ENA|CAJOCC010001921|CAJOCC010001921.1	autosomal	3256.283	0.0045	0.0044	1.02E+00	0.0324
ENA|CAJOCC010001538|CAJOCC010001538.1	autosomal	1874.256	0.00268	0.00262	1.02E+00	0.0327
ENA|CAJOCC010001802|CAJOCC010001802.1	autosomal	2615.83	0.00353	0.00345	1.02E+00	0.0331
ENA|CAJOCC010000702|CAJOCC010000702.1	autosomal	355.634	0.00044	0.00043	1.02E+00	0.0332

ENA|CAJOCC010000014|CAJOCC010000014.1	autosomal	2557.849	0.00349	0.00341	1.02E+00	0.0335
ENA|CAJOCC010001704|CAJOCC010001704.1	autosomal	3608.431	0.0052	0.00508	1.02E+00	0.0337
ENA|CAJOCC010001741|CAJOCC010001741.1	autosomal	866.322	0.0013	0.00127	1.02E+00	0.0337
ENA|CAJOCC010000331|CAJOCC010000331.1	autosomal	1510.282	0.00211	0.00206	1.02E+00	0.0346
ENA|CAJOCC010000072|CAJOCC010000072.1	autosomal	649.425	0.00084	0.00082	1.02E+00	0.0348
ENA|CAJOCC010000272|CAJOCC010000272.1	autosomal	284.383	0.00042	0.00041	1.02E+00	0.0348
ENA|CAJOCC010000237|CAJOCC010000237.1	autosomal	1015.069	0.00125	0.00122	1.02E+00	0.0350
ENA|CAJOCC010001051|CAJOCC010001051.1	autosomal	5461.187	0.00708	0.00691	1.02E+00	0.0351
ENA|CAJOCC010001926|CAJOCC010001926.1	autosomal	9754.672	0.0128	0.01249	1.02E+00	0.0354
ENA|CAJOCC010001635|CAJOCC010001635.1	autosomal	360.764	0.00041	4.00E-04	1.03E+00	0.0356
ENA|CAJOCC010001853|CAJOCC010001853.1	autosomal	1937.48	0.00242	0.00236	1.03E+00	0.0362
ENA|CAJOCC010001897|CAJOCC010001897.1	autosomal	7456.152	0.01	0.00975	1.03E+00	0.0365
ENA|CAJOCC010001942|CAJOCC010001942.1	autosomal	556.228	8.00E-04	0.00078	1.03E+00	0.0365
ENA|CAJOCC010001664|CAJOCC010001664.1	autosomal	7869.553	0.00995	0.0097	1.03E+00	0.0367
ENA|CAJOCC010001670|CAJOCC010001670.1	autosomal	7494.891	0.01022	0.00996	1.03E+00	0.0372
ENA|CAJOCC010000430|CAJOCC010000430.1	autosomal	2040.779	0.00275	0.00268	1.03E+00	0.0372
ENA|CAJOCC010000144|CAJOCC010000144.1	autosomal	276.971	0.00039	0.00038	1.03E+00	0.0375
ENA|CAJOCC010000485|CAJOCC010000485.1	autosomal	331.849	0.00039	0.00038	1.03E+00	0.0375
ENA|CAJOCC010001362|CAJOCC010001362.1	autosomal	458.083	0.00039	0.00038	1.03E+00	0.0375
ENA|CAJOCC010000966|CAJOCC010000966.1	autosomal	8469.088	0.01168	0.01138	1.03E+00	0.0375
ENA|CAJOCC010000396|CAJOCC010000396.1	autosomal	1138.653	0.00155	0.00151	1.03E+00	0.0377
ENA|CAJOCC010000882|CAJOCC010000882.1	autosomal	3362.663	0.00463	0.00451	1.03E+00	0.0379
ENA|CAJOCC010001698|CAJOCC010001698.1	autosomal	525.801	0.00077	0.00075	1.03E+00	0.0380
ENA|CAJOCC010000205|CAJOCC010000205.1	autosomal	864.085	0.00115	0.00112	1.03E+00	0.0381
ENA|CAJOCC010001838|CAJOCC010001838.1	autosomal	896.219	0.00115	0.00112	1.03E+00	0.0381
ENA|CAJOCC010001778|CAJOCC010001778.1	autosomal	5527.469	0.00762	0.00742	1.03E+00	0.0384
ENA|CAJOCC010000147|CAJOCC010000147.1	autosomal	562.02	0.00075	0.00073	1.03E+00	0.0390
ENA|CAJOCC010001912|CAJOCC010001912.1	autosomal	867.744	0.00112	0.00109	1.03E+00	0.0392
ENA|CAJOCC010001932|CAJOCC010001932.1	autosomal	6844.495	0.00844	0.00821	1.03E+00	0.0399
ENA|CAJOCC010001934|CAJOCC010001934.1	autosomal	9137.612	0.01205	0.01172	1.03E+00	0.0401
ENA|CAJOCC010000001|CAJOCC010000001.1	autosomal	4208.005	0.00584	0.00568	1.03E+00	0.0401
ENA|CAJOCC010001850|CAJOCC010001850.1	autosomal	1017.037	0.00146	0.00142	1.03E+00	0.0401

ENA|CAJOCC010000907|CAJOCC010000907.1	autosomal	722.255	0.00107	0.00104	1.03E+00	0.0410
ENA|CAJOCC010000466|CAJOCC010000466.1	autosomal	568.217	0.00071	0.00069	1.03E+00	0.0412
ENA|CAJOCC010000164|CAJOCC010000164.1	autosomal	3254.389	0.00425	0.00413	1.03E+00	0.0413
ENA|CAJOCC010001226|CAJOCC010001226.1	autosomal	3090.253	0.00421	0.00409	1.03E+00	0.0417
ENA|CAJOCC010000425|CAJOCC010000425.1	autosomal	522.652	0.00069	0.00067	1.03E+00	0.0424
ENA|CAJOCC010001735|CAJOCC010001735.1	autosomal	495.602	0.00069	0.00067	1.03E+00	0.0424
ENA|CAJOCC010001806|CAJOCC010001806.1	autosomal	2851.248	0.00379	0.00368	1.03E+00	0.0425
ENA|CAJOCC010001882|CAJOCC010001882.1	autosomal	8250.349	0.01137	0.01104	1.03E+00	0.0425
ENA|CAJOCC010001779|CAJOCC010001779.1	autosomal	1010.745	0.00137	0.00133	1.03E+00	0.0427
ENA|CAJOCC010001648|CAJOCC010001648.1	autosomal	3570.771	0.00477	0.00463	1.03E+00	0.0430
ENA|CAJOCC010001633|CAJOCC010001633.1	autosomal	2075.758	0.00272	0.00264	1.03E+00	0.0431
ENA|CAJOCC010001376|CAJOCC010001376.1	autosomal	2031.972	0.00268	0.0026	1.03E+00	0.0437
ENA|CAJOCC010000535|CAJOCC010000535.1	autosomal	1585.389	0.00199	0.00193	1.03E+00	0.0442
ENA|CAJOCC010000248|CAJOCC010000248.1	autosomal	267.189	0.00033	0.00032	1.03E+00	0.0444
ENA|CAJOCC010001687|CAJOCC010001687.1	autosomal	767.695	0.00098	0.00095	1.03E+00	0.0449
ENA|CAJOCC010000059|CAJOCC010000059.1	autosomal	1916.834	0.0026	0.00252	1.03E+00	0.0451
ENA|CAJOCC010000075|CAJOCC010000075.1	autosomal	485.357	0.00065	0.00063	1.03E+00	0.0451
ENA|CAJOCC010000554|CAJOCC010000554.1	autosomal	714.37	0.00096	0.00093	1.03E+00	0.0458
ENA|CAJOCC010001943|CAJOCC010001943.1	autosomal	3880.886	0.00544	0.00527	1.03E+00	0.0458
ENA|CAJOCC010001869|CAJOCC010001869.1	autosomal	18.152	0.00064	0.00062	1.03E+00	0.0458
ENA|CAJOCC010001879|CAJOCC010001879.1	autosomal	211.131	0.00032	0.00031	1.03E+00	0.0458
ENA|CAJOCC010000479|CAJOCC010000479.1	autosomal	16.552	0.00127	0.00123	1.03E+00	0.0462
ENA|CAJOCC010001875|CAJOCC010001875.1	autosomal	12077.294	0.01548	0.01499	1.03E+00	0.0464
ENA|CAJOCC010001785|CAJOCC010001785.1	autosomal	1591.026	0.00221	0.00214	1.03E+00	0.0464
ENA|CAJOCC010001682|CAJOCC010001682.1	autosomal	1292.141	0.00156	0.00151	1.03E+00	0.0470
ENA|CAJOCC010001817|CAJOCC010001817.1	autosomal	1192.449	0.00156	0.00151	1.03E+00	0.0470
ENA|CAJOCC010001894|CAJOCC010001894.1	autosomal	7160.674	0.00966	0.00935	1.03E+00	0.0471
ENA|CAJOCC010001888|CAJOCC010001888.1	autosomal	259.592	0.00031	3.00E-04	1.03E+00	0.0473
ENA|CAJOCC010001786|CAJOCC010001786.1	autosomal	930.99	0.00123	0.00119	1.03E+00	0.0477
ENA|CAJOCC010001886|CAJOCC010001886.1	autosomal	7499.477	0.00983	0.00951	1.03E+00	0.0477
ENA|CAJOCC010001946|CAJOCC010001946.1	autosomal	4138.529	0.00552	0.00534	1.03E+00	0.0478
ENA|CAJOCC010000173|CAJOCC010000173.1	autosomal	1449.126	0.00184	0.00178	1.03E+00	0.0478

ENA|CAJOCC010000600|CAJOCC010000600.1	autosomal	704.029	0.00092	0.00089	1.03E+00	0.0478
ENA|CAJOCC010000065|CAJOCC010000065.1	autosomal	429.237	0.00061	0.00059	1.03E+00	0.0481
ENA|CAJOCC010000053|CAJOCC010000053.1	autosomal	2356.78	0.00299	0.00289	1.03E+00	0.0491
ENA|CAJOCC010000923|CAJOCC010000923.1	autosomal	7525.002	0.01014	0.0098	1.03E+00	0.0492
ENA|CAJOCC010001662|CAJOCC010001662.1	autosomal	6307.363	0.00852	0.00823	1.04E+00	0.0500
ENA|CAJOCC010001891|CAJOCC010001891.1	autosomal	4946.341	0.00641	0.00619	1.04E+00	0.0504
ENA|CAJOCC010001867|CAJOCC010001867.1	autosomal	1376.608	0.00173	0.00167	1.04E+00	0.0509
ENA|CAJOCC010001807|CAJOCC010001807.1	autosomal	609.161	0.00085	0.00082	1.04E+00	0.0518
ENA|CAJOCC010000701|CAJOCC010000701.1	autosomal	7387.097	0.00986	0.00951	1.04E+00	0.0521
ENA|CAJOCC010001900|CAJOCC010001900.1	autosomal	1279.186	0.00169	0.00163	1.04E+00	0.0522
ENA|CAJOCC010001660|CAJOCC010001660.1	autosomal	1554.739	0.00224	0.00216	1.04E+00	0.0525
ENA|CAJOCC010001696|CAJOCC010001696.1	autosomal	1094.29	0.0014	0.00135	1.04E+00	0.0525
ENA|CAJOCC010001845|CAJOCC010001845.1	autosomal	1223.572	0.00168	0.00162	1.04E+00	0.0525
ENA|CAJOCC010001872|CAJOCC010001872.1	autosomal	2696.044	0.00362	0.00349	1.04E+00	0.0528
ENA|CAJOCC010001037|CAJOCC010001037.1	autosomal	389.249	0.00055	0.00053	1.04E+00	0.0534
ENA|CAJOCC010001016|CAJOCC010001016.1	autosomal	1685.979	0.00216	0.00208	1.04E+00	0.0544
ENA|CAJOCC010001930|CAJOCC010001930.1	autosomal	13339.726	0.01754	0.01688	1.04E+00	0.0553
ENA|CAJOCC010001898|CAJOCC010001898.1	autosomal	5912.803	0.00766	0.00737	1.04E+00	0.0557
ENA|CAJOCC010001938|CAJOCC010001938.1	autosomal	1907.46	0.00263	0.00253	1.04E+00	0.0559
ENA|CAJOCC010000817|CAJOCC010000817.1	autosomal	6701.841	0.00892	0.00858	1.04E+00	0.0561
ENA|CAJOCC010001671|CAJOCC010001671.1	autosomal	2253.655	0.00287	0.00276	1.04E+00	0.0564
ENA|CAJOCC010001877|CAJOCC010001877.1	autosomal	4271.449	0.00574	0.00552	1.04E+00	0.0564
ENA|CAJOCC010000498|CAJOCC010000498.1	autosomal	185.945	0.00026	0.00025	1.04E+00	0.0566
ENA|CAJOCC010001812|CAJOCC010001812.1	autosomal	1078.422	0.00156	0.0015	1.04E+00	0.0566
ENA|CAJOCC010001910|CAJOCC010001910.1	autosomal	1451.441	0.00181	0.00174	1.04E+00	0.0569
ENA|CAJOCC010001666|CAJOCC010001666.1	autosomal	7229.544	0.00916	0.0088	1.04E+00	0.0578
ENA|CAJOCC010001931|CAJOCC010001931.1	autosomal	2466.729	0.0033	0.00317	1.04E+00	0.0580
ENA|CAJOCC010001767|CAJOCC010001767.1	autosomal	1075.808	0.00152	0.00146	1.04E+00	0.0581
ENA|CAJOCC010000543|CAJOCC010000543.1	autosomal	180.013	0.00025	0.00024	1.04E+00	0.0589
ENA|CAJOCC010001831|CAJOCC010001831.1	autosomal	165.229	0.00025	0.00024	1.04E+00	0.0589
ENA|CAJOCC010001881|CAJOCC010001881.1	autosomal	4208.093	0.00574	0.00551	1.04E+00	0.0590
ENA|CAJOCC010001887|CAJOCC010001887.1	autosomal	1581.871	0.00199	0.00191	1.04E+00	0.0592

ENA|CAJOCC010000145|CAJOCC010000145.1	autosomal	8600.033	0.01139	0.01093	1.04E+00	0.0595
ENA|CAJOCC010001394|CAJOCC010001394.1	autosomal	570.735	0.00074	0.00071	1.04E+00	0.0597
ENA|CAJOCC010001727|CAJOCC010001727.1	autosomal	2968.798	0.00391	0.00375	1.04E+00	0.0603
ENA|CAJOCC010001813|CAJOCC010001813.1	autosomal	549.341	0.00073	7.00E-04	1.04E+00	0.0605
ENA|CAJOCC010000288|CAJOCC010000288.1	autosomal	369.105	0.00048	0.00046	1.04E+00	0.0614
ENA|CAJOCC010000419|CAJOCC010000419.1	autosomal	149.385	0.00024	0.00023	1.04E+00	0.0614
ENA|CAJOCC010000432|CAJOCC010000432.1	autosomal	151.706	0.00024	0.00023	1.04E+00	0.0614
ENA|CAJOCC010001619|CAJOCC010001619.1	autosomal	5963.26	0.00788	0.00755	1.04E+00	0.0617
ENA|CAJOCC010001859|CAJOCC010001859.1	autosomal	360.337	0.00047	0.00045	1.04E+00	0.0627
ENA|CAJOCC010000594|CAJOCC010000594.1	autosomal	1248.921	0.00164	0.00157	1.04E+00	0.0629
ENA|CAJOCC010000192|CAJOCC010000192.1	autosomal	2437.464	0.00303	0.0029	1.04E+00	0.0633
ENA|CAJOCC010000215|CAJOCC010000215.1	autosomal	3994.342	0.00509	0.00487	1.05E+00	0.0637
ENA|CAJOCC010000577|CAJOCC010000577.1	autosomal	561.463	0.00069	0.00066	1.05E+00	0.0641
ENA|CAJOCC010000584|CAJOCC010000584.1	autosomal	171.924	0.00023	0.00022	1.05E+00	0.0641
ENA|CAJOCC010001202|CAJOCC010001202.1	autosomal	166.22	0.00023	0.00022	1.05E+00	0.0641
ENA|CAJOCC010001918|CAJOCC010001918.1	autosomal	4913.3	0.00618	0.00591	1.05E+00	0.0644
ENA|CAJOCC010000317|CAJOCC010000317.1	autosomal	511.975	0.00068	0.00065	1.05E+00	0.0651
ENA|CAJOCC010001935|CAJOCC010001935.1	autosomal	10089.821	0.01313	0.01255	1.05E+00	0.0652
ENA|CAJOCC010000487|CAJOCC010000487.1	autosomal	8561.44	0.01127	0.01077	1.05E+00	0.0655
ENA|CAJOCC010001370|CAJOCC010001370.1	autosomal	5271.605	0.00691	0.0066	1.05E+00	0.0662
ENA|CAJOCC010001567|CAJOCC010001567.1	autosomal	7750.396	0.00956	0.00913	1.05E+00	0.0664
ENA|CAJOCC010000869|CAJOCC010000869.1	autosomal	7773.793	0.01019	0.00973	1.05E+00	0.0666
ENA|CAJOCC010000010|CAJOCC010000010.1	autosomal	1382.216	0.00177	0.00169	1.05E+00	0.0667
ENA|CAJOCC010001829|CAJOCC010001829.1	autosomal	1709.438	0.00221	0.00211	1.05E+00	0.0668
ENA|CAJOCC010001846|CAJOCC010001846.1	autosomal	50.781	0.00022	0.00021	1.05E+00	0.0671
ENA|CAJOCC010001945|CAJOCC010001945.1	autosomal	6351.669	0.0083	0.00792	1.05E+00	0.0676
ENA|CAJOCC010001591|CAJOCC010001591.1	autosomal	871.153	0.00109	0.00104	1.05E+00	0.0677
ENA|CAJOCC010001822|CAJOCC010001822.1	autosomal	460.864	0.00065	0.00062	1.05E+00	0.0682
ENA|CAJOCC010000076|CAJOCC010000076.1	autosomal	805.61	0.00108	0.00103	1.05E+00	0.0684
ENA|CAJOCC010001720|CAJOCC010001720.1	autosomal	1861.24	0.00259	0.00247	1.05E+00	0.0684
ENA|CAJOCC010001839|CAJOCC010001839.1	autosomal	2750.509	0.00364	0.00347	1.05E+00	0.0690
ENA|CAJOCC010001796|CAJOCC010001796.1	autosomal	90.312	0.00021	2.00E-04	1.05E+00	0.0704

ENA|CAJOCC010000227|CAJOCC010000227.1	autosomal	947.869	0.00125	0.00119	1.05E+00	0.0710
ENA|CAJOCC010001130|CAJOCC010001130.1	autosomal	3330.911	0.00458	0.00436	1.05E+00	0.0710
ENA|CAJOCC010000314|CAJOCC010000314.1	autosomal	311.935	0.00041	0.00039	1.05E+00	0.0721
ENA|CAJOCC010000299|CAJOCC010000299.1	autosomal	443.518	6.00E-04	0.00057	1.05E+00	0.0740
ENA|CAJOCC010000171|CAJOCC010000171.1	autosomal	294.959	4.00E-04	0.00038	1.05E+00	0.0740
ENA|CAJOCC010000369|CAJOCC010000369.1	autosomal	290.58	4.00E-04	0.00038	1.05E+00	0.0740
ENA|CAJOCC010001406|CAJOCC010001406.1	autosomal	203.2	2.00E-04	0.00019	1.05E+00	0.0740
ENA|CAJOCC010000179|CAJOCC010000179.1	autosomal	609.985	0.00079	0.00075	1.05E+00	0.0750
ENA|CAJOCC010001737|CAJOCC010001737.1	autosomal	2234.212	0.00295	0.0028	1.05E+00	0.0753
ENA|CAJOCC010001631|CAJOCC010001631.1	autosomal	3199.981	0.0039	0.0037	1.05E+00	0.0759
ENA|CAJOCC010001883|CAJOCC010001883.1	autosomal	331.558	0.00039	0.00037	1.05E+00	0.0759
ENA|CAJOCC010001422|CAJOCC010001422.1	autosomal	2042.157	0.00291	0.00276	1.05E+00	0.0764
ENA|CAJOCC010001936|CAJOCC010001936.1	autosomal	4275.16	0.00561	0.00532	1.05E+00	0.0766
ENA|CAJOCC010000316|CAJOCC010000316.1	autosomal	895.675	0.00116	0.0011	1.05E+00	0.0766
ENA|CAJOCC010001739|CAJOCC010001739.1	autosomal	2094.07	0.00288	0.00273	1.05E+00	0.0772
ENA|CAJOCC010000478|CAJOCC010000478.1	autosomal	2007.44	0.00248	0.00235	1.06E+00	0.0777
ENA|CAJOCC010000233|CAJOCC010000233.1	autosomal	443.291	0.00055	0.00052	1.06E+00	0.0809
ENA|CAJOCC010000651|CAJOCC010000651.1	autosomal	4813.078	0.00602	0.00569	1.06E+00	0.0813
ENA|CAJOCC010000008|CAJOCC010000008.1	autosomal	851.711	0.00108	0.00102	1.06E+00	0.0825
ENA|CAJOCC010000239|CAJOCC010000239.1	autosomal	154.754	0.00018	0.00017	1.06E+00	0.0825
ENA|CAJOCC010000863|CAJOCC010000863.1	autosomal	133.234	0.00018	0.00017	1.06E+00	0.0825
ENA|CAJOCC010001914|CAJOCC010001914.1	autosomal	187.508	0.00018	0.00017	1.06E+00	0.0825
ENA|CAJOCC010000346|CAJOCC010000346.1	autosomal	860.344	0.00106	0.001	1.06E+00	0.0841
ENA|CAJOCC010000364|CAJOCC010000364.1	autosomal	399.743	0.00051	0.00048	1.06E+00	0.0875
ENA|CAJOCC010000074|CAJOCC010000074.1	autosomal	1597.581	0.002	0.00188	1.06E+00	0.0893
ENA|CAJOCC010000034|CAJOCC010000034.1	autosomal	1034.716	0.00149	0.0014	1.06E+00	0.0899
ENA|CAJOCC010000218|CAJOCC010000218.1	autosomal	483.209	0.00066	0.00062	1.06E+00	0.0902
ENA|CAJOCC010001929|CAJOCC010001929.1	autosomal	8596.879	0.01161	0.01088	1.07E+00	0.0937
ENA|CAJOCC010000830|CAJOCC010000830.1	autosomal	2393.017	0.00302	0.00282	1.07E+00	0.0989
ENA|CAJOCC010000415|CAJOCC010000415.1	autosomal	75.702	0.00015	0.00014	1.07E+00	0.0995
ENA|CAJOCC010001129|CAJOCC010001129.1	autosomal	1.826	0.00015	0.00014	1.07E+00	0.0995
ENA|CAJOCC010001472|CAJOCC010001472.1	autosomal	244.693	0.00029	0.00027	1.07E+00	0.1031

ENA|CAJOCC010001557|CAJOCC010001557.1	autosomal	231.018	0.00029	0.00027	1.07E+00	0.1031
ENA|CAJOCC010000166|CAJOCC010000166.1	autosomal	322.888	0.00042	0.00039	1.08E+00	0.1069
ENA|CAJOCC010001757|CAJOCC010001757.1	autosomal	981.704	0.00136	0.00126	1.08E+00	0.1102
ENA|CAJOCC010001639|CAJOCC010001639.1	autosomal	857.611	0.00122	0.00113	1.08E+00	0.1106
ENA|CAJOCC010001901|CAJOCC010001901.1	autosomal	495.112	0.00066	0.00061	1.08E+00	0.1137
ENA|CAJOCC010000463|CAJOCC010000463.1	autosomal	24.868	0.00013	0.00012	1.08E+00	0.1155
ENA|CAJOCC010001471|CAJOCC010001471.1	autosomal	123.203	0.00013	0.00012	1.08E+00	0.1155
ENA|CAJOCC010001922|CAJOCC010001922.1	autosomal	105.046	0.00013	0.00012	1.08E+00	0.1155
ENA|CAJOCC010000118|CAJOCC010000118.1	autosomal	2842.285	0.00384	0.00353	1.09E+00	0.1214
ENA|CAJOCC010000325|CAJOCC010000325.1	autosomal	97.825	0.00012	0.00011	1.09E+00	0.1255
ENA|CAJOCC010000413|CAJOCC010000413.1	autosomal	203.301	0.00024	0.00022	1.09E+00	0.1255
ENA|CAJOCC010000733|CAJOCC010000733.1	autosomal	3.232	0.00012	0.00011	1.09E+00	0.1255
ENA|CAJOCC010001034|CAJOCC010001034.1	autosomal	83.685	0.00012	0.00011	1.09E+00	0.1255
ENA|CAJOCC010001675|CAJOCC010001675.1	autosomal	86.115	0.00012	0.00011	1.09E+00	0.1255
ENA|CAJOCC010000435|CAJOCC010000435.1	autosomal	284.477	0.00035	0.00032	1.09E+00	0.1293
ENA|CAJOCC010000098|CAJOCC010000098.1	autosomal	171.091	0.00022	2.00E-04	1.10E+00	0.1375
ENA|CAJOCC010001810|CAJOCC010001810.1	autosomal	157.709	0.00021	0.00019	1.11E+00	0.1444
ENA|CAJOCC010001890|CAJOCC010001890.1	autosomal	161.32	0.00021	0.00019	1.11E+00	0.1444
ENA|CAJOCC010001920|CAJOCC010001920.1	autosomal	301.201	0.00042	0.00038	1.11E+00	0.1444
ENA|CAJOCC010001085|CAJOCC010001085.1	autosomal	23.66	1.00E-04	9.00E-05	1.11E+00	0.1520
ENA|CAJOCC010001399|CAJOCC010001399.1	autosomal	78.767	1.00E-04	9.00E-05	1.11E+00	0.1520
ENA|CAJOCC010001436|CAJOCC010001436.1	autosomal	16.867	1.00E-04	9.00E-05	1.11E+00	0.1520
ENA|CAJOCC010001745|CAJOCC010001745.1	autosomal	73.483	1.00E-04	9.00E-05	1.11E+00	0.1520
ENA|CAJOCC010001598|CAJOCC010001598.1	autosomal	116.974	0.00019	0.00017	1.12E+00	0.1605
ENA|CAJOCC010001551|CAJOCC010001551.1	autosomal	4.223	9.00E-05	8.00E-05	1.13E+00	0.1699
ENA|CAJOCC010000208|CAJOCC010000208.1	autosomal	89.743	8.00E-05	7.00E-05	1.14E+00	0.1926
ENA|CAJOCC010000311|CAJOCC010000311.1	autosomal	62.592	8.00E-05	7.00E-05	1.14E+00	0.1926
ENA|CAJOCC010000559|CAJOCC010000559.1	autosomal	7.326	8.00E-05	7.00E-05	1.14E+00	0.1926
ENA|CAJOCC010000625|CAJOCC010000625.1	autosomal	56.504	8.00E-05	7.00E-05	1.14E+00	0.1926
ENA|CAJOCC010001681|CAJOCC010001681.1	autosomal	184.725	3.00E-04	0.00026	1.15E+00	0.2065
ENA|CAJOCC010000169|CAJOCC010000169.1	autosomal	26.7	7.00E-05	6.00E-05	1.17E+00	0.2224
ENA|CAJOCC010000287|CAJOCC010000287.1	autosomal	60.769	7.00E-05	6.00E-05	1.17E+00	0.2224

ENA|CAJOCC010000510|CAJOCC010000510.1	autosomal	50.995	7.00E-05	6.00E-05	1.17E+00	0.2224
ENA|CAJOCC010001818|CAJOCC010001818.1	autosomal	58.565	7.00E-05	6.00E-05	1.17E+00	0.2224
ENA|CAJOCC010000027|CAJOCC010000027.1	autosomal	348.192	0.00041	0.00035	1.17E+00	0.2283
ENA|CAJOCC010001788|CAJOCC010001788.1	autosomal	376.882	0.00032	0.00027	1.19E+00	0.2451
ENA|CAJOCC010001860|CAJOCC010001860.1	autosomal	1158.245	0.00164	0.00138	1.19E+00	0.2490
ENA|CAJOCC010001915|CAJOCC010001915.1	autosomal	661.715	0.00127	0.00106	1.20E+00	0.2608
ENA|CAJOCC010000975|CAJOCC010000975.1	autosomal	13.013	6.00E-05	5.00E-05	1.20E+00	0.2630
ENA|CAJOCC010001458|CAJOCC010001458.1	autosomal	3.551	6.00E-05	5.00E-05	1.20E+00	0.2630
ENA|CAJOCC010000013|CAJOCC010000013.1	autosomal	265.925	0.00053	0.00044	1.20E+00	0.2685
ENA|CAJOCC010001210|CAJOCC010001210.1	autosomal	50.522	0.00011	9.00E-05	1.22E+00	0.2895
ENA|CAJOCC010001319|CAJOCC010001319.1	autosomal	39.683	0.00011	9.00E-05	1.22E+00	0.2895
ENA|CAJOCC010001142|CAJOCC010001142.1	autosomal	20.403	0.00021	0.00017	1.24E+00	0.3049
ENA|CAJOCC010000091|CAJOCC010000091.1	autosomal	24.148	5.00E-05	4.00E-05	1.25E+00	0.3219
ENA|CAJOCC010000221|CAJOCC010000221.1	autosomal	7.096	5.00E-05	4.00E-05	1.25E+00	0.3219
ENA|CAJOCC010000439|CAJOCC010000439.1	autosomal	5.738	5.00E-05	4.00E-05	1.25E+00	0.3219
ENA|CAJOCC010000496|CAJOCC010000496.1	autosomal	28.318	5.00E-05	4.00E-05	1.25E+00	0.3219
ENA|CAJOCC010000506|CAJOCC010000506.1	autosomal	166.741	5.00E-05	4.00E-05	1.25E+00	0.3219
ENA|CAJOCC010000736|CAJOCC010000736.1	autosomal	63.374	5.00E-05	4.00E-05	1.25E+00	0.3219
ENA|CAJOCC010000910|CAJOCC010000910.1	autosomal	9.211	5.00E-05	4.00E-05	1.25E+00	0.3219
ENA|CAJOCC010001115|CAJOCC010001115.1	autosomal	12.147	5.00E-05	4.00E-05	1.25E+00	0.3219
ENA|CAJOCC010001516|CAJOCC010001516.1	autosomal	36.334	5.00E-05	4.00E-05	1.25E+00	0.3219
ENA|CAJOCC010001527|CAJOCC010001527.1	autosomal	9.826	5.00E-05	4.00E-05	1.25E+00	0.3219
ENA|CAJOCC010001689|CAJOCC010001689.1	autosomal	132.394	0.00015	0.00012	1.25E+00	0.3219
ENA|CAJOCC010001732|CAJOCC010001732.1	autosomal	63.155	5.00E-05	4.00E-05	1.25E+00	0.3219
ENA|CAJOCC010001933|CAJOCC010001933.1	autosomal	55.938	5.00E-05	4.00E-05	1.25E+00	0.3219
ENA|CAJOCC010000955|CAJOCC010000955.1	autosomal	40.475	0.00014	0.00011	1.27E+00	0.3479
ENA|CAJOCC010000185|CAJOCC010000185.1	autosomal	34.858	9.00E-05	7.00E-05	1.29E+00	0.3626
ENA|CAJOCC010001827|CAJOCC010001827.1	autosomal	61.337	0.00036	0.00028	1.29E+00	0.3626
ENA|CAJOCC010001714|CAJOCC010001714.1	autosomal	163.833	4.00E-04	0.00031	1.29E+00	0.3677
ENA|CAJOCC010000006|CAJOCC010000006.1	autosomal	24.573	4.00E-05	3.00E-05	1.33E+00	0.4150
ENA|CAJOCC010000187|CAJOCC010000187.1	autosomal	8.919	8.00E-05	6.00E-05	1.33E+00	0.4150
ENA|CAJOCC010000404|CAJOCC010000404.1	autosomal	13.516	4.00E-05	3.00E-05	1.33E+00	0.4150

ENA|CAJOCC010000500|CAJOCC010000500.1	autosomal	61.034	4.00E-05	3.00E-05	1.33E+00	0.4150
ENA|CAJOCC010000723|CAJOCC010000723.1	autosomal	46.802	4.00E-05	3.00E-05	1.33E+00	0.4150
ENA|CAJOCC010000735|CAJOCC010000735.1	autosomal	101.366	4.00E-05	3.00E-05	1.33E+00	0.4150
ENA|CAJOCC010000839|CAJOCC010000839.1	autosomal	28.112	4.00E-05	3.00E-05	1.33E+00	0.4150
ENA|CAJOCC010000896|CAJOCC010000896.1	autosomal	26.25	4.00E-05	3.00E-05	1.33E+00	0.4150
ENA|CAJOCC010000972|CAJOCC010000972.1	autosomal	38.916	4.00E-05	3.00E-05	1.33E+00	0.4150
ENA|CAJOCC010001044|CAJOCC010001044.1	autosomal	12.935	4.00E-05	3.00E-05	1.33E+00	0.4150
ENA|CAJOCC010001146|CAJOCC010001146.1	autosomal	16.97	4.00E-05	3.00E-05	1.33E+00	0.4150
ENA|CAJOCC010001197|CAJOCC010001197.1	autosomal	34.081	4.00E-05	3.00E-05	1.33E+00	0.4150
ENA|CAJOCC010001417|CAJOCC010001417.1	autosomal	53.88	4.00E-05	3.00E-05	1.33E+00	0.4150
ENA|CAJOCC010000414|CAJOCC010000414.1	autosomal	248.16	0.00047	0.00035	1.34E+00	0.4253
ENA|CAJOCC010000289|CAJOCC010000289.1	autosomal	44.078	0.00011	8.00E-05	1.38E+00	0.4594
ENA|CAJOCC010000088|CAJOCC010000088.1	autosomal	78.062	0.00014	1.00E-04	1.40E+00	0.4854
ENA|CAJOCC010000741|CAJOCC010000741.1	autosomal	57.51	0.00014	1.00E-04	1.40E+00	0.4854
ENA|CAJOCC010001795|CAJOCC010001795.1	autosomal	42.721	7.00E-05	5.00E-05	1.40E+00	0.4854
ENA|CAJOCC010000806|CAJOCC010000806.1	autosomal	25.707	0.00026	0.00018	1.44E+00	0.5305
ENA|CAJOCC010001386|CAJOCC010001386.1	autosomal	16.066	0.00013	9.00E-05	1.44E+00	0.5305
ENA|CAJOCC010000116|CAJOCC010000116.1	autosomal	35.989	3.00E-05	2.00E-05	1.50E+00	0.5850
ENA|CAJOCC010000130|CAJOCC010000130.1	autosomal	69.983	6.00E-05	4.00E-05	1.50E+00	0.5850
ENA|CAJOCC010000154|CAJOCC010000154.1	autosomal	69.261	3.00E-05	2.00E-05	1.50E+00	0.5850
ENA|CAJOCC010000182|CAJOCC010000182.1	autosomal	3.335	3.00E-05	2.00E-05	1.50E+00	0.5850
ENA|CAJOCC010000191|CAJOCC010000191.1	autosomal	52.895	6.00E-05	4.00E-05	1.50E+00	0.5850
ENA|CAJOCC010000244|CAJOCC010000244.1	autosomal	18.145	0.00015	1.00E-04	1.50E+00	0.5850
ENA|CAJOCC010000395|CAJOCC010000395.1	autosomal	37.663	3.00E-05	2.00E-05	1.50E+00	0.5850
ENA|CAJOCC010000567|CAJOCC010000567.1	autosomal	15.356	3.00E-05	2.00E-05	1.50E+00	0.5850
ENA|CAJOCC010000606|CAJOCC010000606.1	autosomal	23.011	3.00E-05	2.00E-05	1.50E+00	0.5850
ENA|CAJOCC010000607|CAJOCC010000607.1	autosomal	70.642	6.00E-05	4.00E-05	1.50E+00	0.5850
ENA|CAJOCC010000721|CAJOCC010000721.1	autosomal	2.35	3.00E-05	2.00E-05	1.50E+00	0.5850
ENA|CAJOCC010000771|CAJOCC010000771.1	autosomal	1.236	3.00E-05	2.00E-05	1.50E+00	0.5850
ENA|CAJOCC010000860|CAJOCC010000860.1	autosomal	64.087	6.00E-05	4.00E-05	1.50E+00	0.5850
ENA|CAJOCC010000926|CAJOCC010000926.1	autosomal	28.898	3.00E-05	2.00E-05	1.50E+00	0.5850
ENA|CAJOCC010001045|CAJOCC010001045.1	autosomal	30.109	3.00E-05	2.00E-05	1.50E+00	0.5850

ENA|CAJOCC010001240|CAJOCC010001240.1	autosomal	44.522	3.00E-05	2.00E-05	1.50E+00	0.5850
ENA|CAJOCC010001292|CAJOCC010001292.1	autosomal	12.954	3.00E-05	2.00E-05	1.50E+00	0.5850
ENA|CAJOCC010001297|CAJOCC010001297.1	autosomal	11.564	3.00E-05	2.00E-05	1.50E+00	0.5850
ENA|CAJOCC010001309|CAJOCC010001309.1	autosomal	153.713	3.00E-04	2.00E-04	1.50E+00	0.5850
ENA|CAJOCC010001398|CAJOCC010001398.1	autosomal	29.915	3.00E-05	2.00E-05	1.50E+00	0.5850
ENA|CAJOCC010001502|CAJOCC010001502.1	autosomal	9.938	3.00E-05	2.00E-05	1.50E+00	0.5850
ENA|CAJOCC010001546|CAJOCC010001546.1	autosomal	34.81	3.00E-05	2.00E-05	1.50E+00	0.5850
ENA|CAJOCC010001568|CAJOCC010001568.1	autosomal	54.958	3.00E-05	2.00E-05	1.50E+00	0.5850
ENA|CAJOCC010001569|CAJOCC010001569.1	autosomal	16.47	3.00E-05	2.00E-05	1.50E+00	0.5850
ENA|CAJOCC010001637|CAJOCC010001637.1	autosomal	71.364	3.00E-05	2.00E-05	1.50E+00	0.5850
ENA|CAJOCC010001834|CAJOCC010001834.1	autosomal	39.024	3.00E-05	2.00E-05	1.50E+00	0.5850
ENA|CAJOCC010001856|CAJOCC010001856.1	autosomal	21.163	3.00E-05	2.00E-05	1.50E+00	0.5850
ENA|CAJOCC010001218|CAJOCC010001218.1	autosomal	67.407	9.00E-05	6.00E-05	1.50E+00	0.5850
ENA|CAJOCC010001800|CAJOCC010001800.1	autosomal	235.727	0.00072	0.00047	1.53E+00	0.6153
ENA|CAJOCC010001206|CAJOCC010001206.1	autosomal	110.565	0.00025	0.00016	1.56E+00	0.6439
ENA|CAJOCC010001728|CAJOCC010001728.1	autosomal	100.527	0.00025	0.00016	1.56E+00	0.6439
ENA|CAJOCC010000483|CAJOCC010000483.1	autosomal	15.379	0.00022	0.00014	1.57E+00	0.6521
ENA|CAJOCC010001769|CAJOCC010001769.1	autosomal	170.469	0.00011	7.00E-05	1.57E+00	0.6521
ENA|CAJOCC010000939|CAJOCC010000939.1	autosomal	26.689	0.00016	1.00E-04	1.60E+00	0.6781
ENA|CAJOCC010001680|CAJOCC010001680.1	autosomal	58.249	8.00E-05	5.00E-05	1.60E+00	0.6781
ENA|CAJOCC010000225|CAJOCC010000225.1	autosomal	86.957	0.00021	0.00013	1.62E+00	0.6919
ENA|CAJOCC010000731|CAJOCC010000731.1	autosomal	4.064	5.00E-05	3.00E-05	1.67E+00	0.7370
ENA|CAJOCC010000759|CAJOCC010000759.1	autosomal	41.163	5.00E-05	3.00E-05	1.67E+00	0.7370
ENA|CAJOCC010001459|CAJOCC010001459.1	autosomal	76.926	5.00E-05	3.00E-05	1.67E+00	0.7370
ENA|CAJOCC010001531|CAJOCC010001531.1	autosomal	37.672	5.00E-05	3.00E-05	1.67E+00	0.7370
ENA|CAJOCC010001821|CAJOCC010001821.1	autosomal	7.06	5.00E-05	3.00E-05	1.67E+00	0.7370
ENA|CAJOCC010000407|CAJOCC010000407.1	autosomal	76.047	0.00012	7.00E-05	1.71E+00	0.7776
ENA|CAJOCC010000073|CAJOCC010000073.1	autosomal	43.202	7.00E-05	4.00E-05	1.75E+00	0.8074
ENA|CAJOCC010001152|CAJOCC010001152.1	autosomal	56.722	7.00E-05	4.00E-05	1.75E+00	0.8074
ENA|CAJOCC010000110|CAJOCC010000110.1	y_contig	20.096	2.00E-05	1.00E-05	2.00E+00	1.0000
ENA|CAJOCC010000203|CAJOCC010000203.1	y_contig	32.315	2.00E-05	1.00E-05	2.00E+00	1.0000
ENA|CAJOCC010000206|CAJOCC010000206.1	y_contig	2.755	2.00E-05	1.00E-05	2.00E+00	1.0000

ENA|CAJOCC010000265|CAJOCC010000265.1	y_contig	37.351	2.00E-05	1.00E-05	2.00E+00	1.0000
ENA|CAJOCC010000273|CAJOCC010000273.1	y_contig	27.655	2.00E-05	1.00E-05	2.00E+00	1.0000
ENA|CAJOCC010000340|CAJOCC010000340.1	y_contig	2.737	2.00E-05	1.00E-05	2.00E+00	1.0000
ENA|CAJOCC010000469|CAJOCC010000469.1	y_contig	27.805	2.00E-05	1.00E-05	2.00E+00	1.0000
ENA|CAJOCC010000497|CAJOCC010000497.1	y_contig	16.352	2.00E-05	1.00E-05	2.00E+00	1.0000
ENA|CAJOCC010000508|CAJOCC010000508.1	y_contig	15.237	2.00E-05	1.00E-05	2.00E+00	1.0000
ENA|CAJOCC010000529|CAJOCC010000529.1	y_contig	52.138	2.00E-05	1.00E-05	2.00E+00	1.0000
ENA|CAJOCC010000536|CAJOCC010000536.1	y_contig	22.886	2.00E-05	1.00E-05	2.00E+00	1.0000
ENA|CAJOCC010000537|CAJOCC010000537.1	y_contig	26.498	8.00E-05	4.00E-05	2.00E+00	1.0000
ENA|CAJOCC010000571|CAJOCC010000571.1	y_contig	3.507	2.00E-05	1.00E-05	2.00E+00	1.0000
ENA|CAJOCC010000642|CAJOCC010000642.1	y_contig	22.372	4.00E-05	2.00E-05	2.00E+00	1.0000
ENA|CAJOCC010000656|CAJOCC010000656.1	y_contig	13.602	2.00E-05	1.00E-05	2.00E+00	1.0000
ENA|CAJOCC010000664|CAJOCC010000664.1	y_contig	25.173	2.00E-05	1.00E-05	2.00E+00	1.0000
ENA|CAJOCC010000746|CAJOCC010000746.1	y_contig	41.952	2.00E-05	1.00E-05	2.00E+00	1.0000
ENA|CAJOCC010000887|CAJOCC010000887.1	y_contig	4.087	2.00E-05	1.00E-05	2.00E+00	1.0000
ENA|CAJOCC010000928|CAJOCC010000928.1	y_contig	18.243	4.00E-05	2.00E-05	2.00E+00	1.0000
ENA|CAJOCC010001025|CAJOCC010001025.1	y_contig	25.928	4.00E-05	2.00E-05	2.00E+00	1.0000
ENA|CAJOCC010001031|CAJOCC010001031.1	y_contig	11.078	4.00E-05	2.00E-05	2.00E+00	1.0000
ENA|CAJOCC010001050|CAJOCC010001050.1	y_contig	13.466	2.00E-05	1.00E-05	2.00E+00	1.0000
ENA|CAJOCC010001053|CAJOCC010001053.1	y_contig	13.084	2.00E-05	1.00E-05	2.00E+00	1.0000
ENA|CAJOCC010001071|CAJOCC010001071.1	y_contig	8.839	2.00E-05	1.00E-05	2.00E+00	1.0000
ENA|CAJOCC010001178|CAJOCC010001178.1	y_contig	7.197	2.00E-05	1.00E-05	2.00E+00	1.0000
ENA|CAJOCC010001275|CAJOCC010001275.1	y_contig	33.954	6.00E-05	3.00E-05	2.00E+00	1.0000
ENA|CAJOCC010001484|CAJOCC010001484.1	y_contig	11.54	2.00E-05	1.00E-05	2.00E+00	1.0000
ENA|CAJOCC010001513|CAJOCC010001513.1	y_contig	29.896	6.00E-05	3.00E-05	2.00E+00	1.0000
ENA|CAJOCC010001533|CAJOCC010001533.1	y_contig	16.381	2.00E-05	1.00E-05	2.00E+00	1.0000
ENA|CAJOCC010001534|CAJOCC010001534.1	y_contig	9.837	2.00E-05	1.00E-05	2.00E+00	1.0000
ENA|CAJOCC010001617|CAJOCC010001617.1	y_contig	11.31	2.00E-05	1.00E-05	2.00E+00	1.0000
ENA|CAJOCC010001636|CAJOCC010001636.1	y_contig	26.868	2.00E-05	1.00E-05	2.00E+00	1.0000
ENA|CAJOCC010001649|CAJOCC010001649.1	y_contig	26.156	2.00E-05	1.00E-05	2.00E+00	1.0000
ENA|CAJOCC010001797|CAJOCC010001797.1	y_contig	44.186	2.00E-05	1.00E-05	2.00E+00	1.0000
ENA|CAJOCC010001842|CAJOCC010001842.1	y_contig	32.37	2.00E-05	1.00E-05	2.00E+00	1.0000

ENA|CAJOCC010001870|CAJOCC010001870.1	y_contig	7.604	2.00E-05	1.00E-05	2.00E+00	1.0000
ENA|CAJOCC010000099|CAJOCC010000099.1	y_contig	17.028	0.00017	8.00E-05	2.13E+00	1.0875
ENA|CAJOCC010000420|CAJOCC010000420.1	y_contig	28.923	0.00011	5.00E-05	2.20E+00	1.1375
ENA|CAJOCC010001774|CAJOCC010001774.1	y_contig	116.016	0.00011	5.00E-05	2.20E+00	1.1375
ENA|CAJOCC010000092|CAJOCC010000092.1	y_contig	62.739	5.00E-05	2.00E-05	2.50E+00	1.3219
ENA|CAJOCC010001358|CAJOCC010001358.1	y_contig	30.67	5.00E-05	2.00E-05	2.50E+00	1.3219
ENA|CAJOCC010001488|CAJOCC010001488.1	y_contig	21.264	5.00E-05	2.00E-05	2.50E+00	1.3219
ENA|CAJOCC010001699|CAJOCC010001699.1	y_contig	45.271	0.00018	7.00E-05	2.57E+00	1.3626
ENA|CAJOCC010001808|CAJOCC010001808.1	y_contig	33.011	0.00013	5.00E-05	2.60E+00	1.3785
ENA|CAJOCC010000097|CAJOCC010000097.1	y_contig	19.085	8.00E-05	3.00E-05	2.67E+00	1.4150
ENA|CAJOCC010000103|CAJOCC010000103.1	y_contig	187.103	8.00E-05	3.00E-05	2.67E+00	1.4150
ENA|CAJOCC010001166|CAJOCC010001166.1	y_contig	20.587	8.00E-05	3.00E-05	2.67E+00	1.4150
ENA|CAJOCC010001192|CAJOCC010001192.1	y_contig	31.413	0.00016	6.00E-05	2.67E+00	1.4150
ENA|CAJOCC010001542|CAJOCC010001542.1	y_contig	25.757	0.00017	6.00E-05	2.83E+00	1.5025
ENA|CAJOCC010000134|CAJOCC010000134.1	y_contig	35.97	0.00012	4.00E-05	3.00E+00	1.5850
ENA|CAJOCC010000137|CAJOCC010000137.1	y_contig	13.884	3.00E-05	1.00E-05	3.00E+00	1.5850
ENA|CAJOCC010000181|CAJOCC010000181.1	y_contig	7.231	3.00E-05	1.00E-05	3.00E+00	1.5850
ENA|CAJOCC010000326|CAJOCC010000326.1	y_contig	23.778	3.00E-05	1.00E-05	3.00E+00	1.5850
ENA|CAJOCC010000647|CAJOCC010000647.1	y_contig	12.857	3.00E-05	1.00E-05	3.00E+00	1.5850
ENA|CAJOCC010000694|CAJOCC010000694.1	y_contig	49.41	0.00015	5.00E-05	3.00E+00	1.5850
ENA|CAJOCC010000769|CAJOCC010000769.1	y_contig	30.632	3.00E-05	1.00E-05	3.00E+00	1.5850
ENA|CAJOCC010000895|CAJOCC010000895.1	y_contig	6.811	3.00E-05	1.00E-05	3.00E+00	1.5850
ENA|CAJOCC010000900|CAJOCC010000900.1	y_contig	54.972	6.00E-05	2.00E-05	3.00E+00	1.5850
ENA|CAJOCC010001429|CAJOCC010001429.1	y_contig	20.664	0.00012	4.00E-05	3.00E+00	1.5850
ENA|CAJOCC010001447|CAJOCC010001447.1	y_contig	24.248	3.00E-05	1.00E-05	3.00E+00	1.5850
ENA|CAJOCC010001461|CAJOCC010001461.1	y_contig	20.273	3.00E-05	1.00E-05	3.00E+00	1.5850
ENA|CAJOCC010001464|CAJOCC010001464.1	y_contig	53.43	3.00E-05	1.00E-05	3.00E+00	1.5850
ENA|CAJOCC010001630|CAJOCC010001630.1	y_contig	20.45	3.00E-05	1.00E-05	3.00E+00	1.5850
ENA|CAJOCC010001388|CAJOCC010001388.1	y_contig	17.527	0.00016	5.00E-05	3.20E+00	1.6781
ENA|CAJOCC010001052|CAJOCC010001052.1	y_contig	1.043	7.00E-05	2.00E-05	3.50E+00	1.8074
ENA|CAJOCC010001378|CAJOCC010001378.1	y_contig	21.889	7.00E-05	2.00E-05	3.50E+00	1.8074
ENA|CAJOCC010001528|CAJOCC010001528.1	y_contig	18.464	7.00E-05	2.00E-05	3.50E+00	1.8074

ENA|CAJOCC010001467|CAJOCC010001467.1	y_contig	21.255	0.00019	5.00E-05	3.80E+00	1.9260
ENA|CAJOCC010000055|CAJOCC010000055.1	y_contig	25.879	8.00E-05	2.00E-05	4.00E+00	2.0000
ENA|CAJOCC010000377|CAJOCC010000377.1	y_contig	51.614	4.00E-05	1.00E-05	4.00E+00	2.0000
ENA|CAJOCC010000722|CAJOCC010000722.1	y_contig	23.954	4.00E-05	1.00E-05	4.00E+00	2.0000
ENA|CAJOCC010001008|CAJOCC010001008.1	y_contig	15.145	8.00E-05	2.00E-05	4.00E+00	2.0000
ENA|CAJOCC010001137|CAJOCC010001137.1	y_contig	0.896	4.00E-05	1.00E-05	4.00E+00	2.0000
ENA|CAJOCC010001507|CAJOCC010001507.1	y_contig	22.973	4.00E-05	1.00E-05	4.00E+00	2.0000
ENA|CAJOCC010001678|CAJOCC010001678.1	y_contig	44.293	8.00E-05	2.00E-05	4.00E+00	2.0000
ENA|CAJOCC010001811|CAJOCC010001811.1	y_contig	17.772	4.00E-05	1.00E-05	4.00E+00	2.0000
ENA|CAJOCC010001843|CAJOCC010001843.1	y_contig	13.776	4.00E-05	1.00E-05	4.00E+00	2.0000
ENA|CAJOCC010001857|CAJOCC010001857.1	y_contig	31.875	4.00E-05	1.00E-05	4.00E+00	2.0000
ENA|CAJOCC010001784|CAJOCC010001784.1	y_contig	11.926	0.00021	5.00E-05	4.20E+00	2.0704
ENA|CAJOCC010000818|CAJOCC010000818.1	y_contig	4.705	0.00017	4.00E-05	4.25E+00	2.0875
ENA|CAJOCC010000864|CAJOCC010000864.1	y_contig	15.269	0.00057	0.00013	4.38E+00	2.1325
ENA|CAJOCC010000845|CAJOCC010000845.1	y_contig	37.703	0.00022	5.00E-05	4.40E+00	2.1375
ENA|CAJOCC010001329|CAJOCC010001329.1	y_contig	13.188	0.00022	5.00E-05	4.40E+00	2.1375
ENA|CAJOCC010000501|CAJOCC010000501.1	y_contig	29.823	9.00E-05	2.00E-05	4.50E+00	2.1699
ENA|CAJOCC010000673|CAJOCC010000673.1	y_contig	23.74	0.00014	3.00E-05	4.67E+00	2.2224
ENA|CAJOCC010001258|CAJOCC010001258.1	y_contig	23.178	5.00E-05	1.00E-05	5.00E+00	2.3219
ENA|CAJOCC010001669|CAJOCC010001669.1	y_contig	73.646	5.00E-05	1.00E-05	5.00E+00	2.3219
ENA|CAJOCC010001216|CAJOCC010001216.1	y_contig	2.615	0.00016	3.00E-05	5.33E+00	2.4150
ENA|CAJOCC010000551|CAJOCC010000551.1	y_contig	46.343	7.00E-05	1.00E-05	7.00E+00	2.8074
ENA|CAJOCC010001334|CAJOCC010001334.1	y_contig	30.116	7.00E-05	1.00E-05	7.00E+00	2.8074
ENA|CAJOCC010000556|CAJOCC010000556.1	y_contig	4.829	8.00E-05	1.00E-05	8.00E+00	3.0000
ENA|CAJOCC010001580|CAJOCC010001580.1	y_contig	48.71	8.00E-05	1.00E-05	8.00E+00	3.0000
ENA|CAJOCC010001701|CAJOCC010001701.1	y_contig	54.173	9.00E-05	1.00E-05	9.00E+00	3.1699
ENA|CAJOCC010000829|CAJOCC010000829.1	y_contig	15.329	1.00E-04	1.00E-05	1.00E+01	3.3219
ENA|CAJOCC010001359|CAJOCC010001359.1	y_contig	35.111	1.00E-04	1.00E-05	1.00E+01	3.3219
ENA|CAJOCC010000063|CAJOCC010000063.1	y_contig	31.348	0.00026	1.00E-05	2.60E+01	4.7004

Supplemental Table 6. Data availability. Project ID beginning in 'PRJNA' can be accessed through the National Center for Biotechnology Information Sequencing Read Archive (https://www.ncbi.nlm.nih.gov/sra). Project ID beginning with 'PRJEB' can be accessed in European Bioinformatics Institute's European Nucleotide Archive (https://www.ebi.ac.uk/ena). Scripts and pipelines can be found at https://github.com/ljmfong/Poecilia_picta_Evol_Hist

Species	Data Type	Project ID
P. picta	RNA-Seq	PRJNA856299
P. reticulata	RNA-Seq	PRJEB39998
P. wingei	RNA-Seq	PRJEB39998
P. reticulata	DNA-Seq Paired-End	PRJNA858015
P. wingei	DNA-Seq Paired-End	PRJNA528814
P. picta	DNA-Seq Paired-End	PRJNA528814
P. parae	DNA-Seq Paired-End	PRJNA714257
P. picta	Genome Assembly	PRJNA862953

Pipelines and scripts can be found at https://github.com/ljmfong/Poecilia_picta_Evol_Hist

Supplemental Table 7. Sequencing statistics of individuals and their respective families.

Family	Individual	Sex	Untrimmed Reads	Trimmed Reads
	Dame	Female	139,156,351	266,121,840
	F1	Female	102,460,735	195,269,844
	F2	Female	71,380,907	136,325,452
	F3	Female	55,902,033	107,025,652
	F4	Female	93,872,399	179,552,078
G3	F5	Female	46,007,780	87,899,032
	M10	Male	61,004,729	116,639,716
	M11	Male	70,481,997	134,845,296
	M3	Male	42,995,381	80,362,900
	M6	Male	39,181,469	74,065,600
	M7	Male	63,775,971	120,399,324
	Sire	Male	53,608,654	102,280,490
	Dame	Female	46,870,797	89,221,548
	F1	Female	71,699,389	137,467,330
	F2	Female	73,243,228	140,126,418
	F3	Female	60,610,985	115,526,626
	F4	Female	67,808,839	129,647,610
G5	F6	Female	80,932,127	155,941,940
	M1	Male	92,016,896	177,686,072
	M2	Male	89,326,145	172,507,784
	M4	Male	75,563,218	145,780,888
	M5	Male	81,090,676	156,385,360
	M6	Male	77,480,797	149,773,652
	Sire	Male	61,468,893	118,871,490
	Dame	Female	68,239,055	132,345,448
	F1	Female	86,050,656	166,188,898
	F2	Female	90,082,129	172,830,594
	F3	Female	81,820,496	157,315,556
	F5	Female	68,529,927	133,959,084

T1	F6	Female	41,983,935	79,847,576
	M3	Male	99,293,931	191,628,256
	M5	Male	80,728,473	155,830,096
	M6	Male	107,744,661	206,927,364
	M7	Male	89,760,470	172,414,844
	M8	Male	78,462,125	151,905,610
	Sire	Male	69,460,889	133,735,134
	Dame	Female	75,848,791	145,538,446
	F1	Female	71,146,167	134,132,728
	F3	Female	83,365,974	161,514,930
	F4	Female	76,822,488	147,902,476
	F6	Female	103,390,369	199,606,444
T13	F7	Female	96,147,498	185,898,404
	M2	Male	79,093,302	53,396,430
	M3	Male	102,733,482	199,453,602
	M5	Male	84,363,344	163,299,754
	M7	Male	78,086,967	150,936,426
	M9	Male	80,301,722	155,509,218
	Sire	Male	79,479,698	153,712,260

Supplemental Table 8. Summary statistics of number of DNA-seq paired reads and their coverage from different studies. Paired reads and reported coverage is after trimming. *P. picta* and *P. wingei* statistics are reported from Darolti, et al. 2019 and *P. parae* statistics are reported from Sandkam, et al. 2021.

Species	Individual	Sex	No. of Paired Reads (Trimmed)	Anticipated Coverage
P. picta	247	Female	201,783,529	70X
	248	Female	248,146,529	86X
	265	Female	251,440,989	87X
	266	Male	264,471,289	91X
	267	Male	209,266,241	72X
	268	Male	213,098,477	74X
P. wingei	291	Female	222,019,309	77X
	292	Female	209,095,391	72X
	293	Female	244,778,587	85X
	294	Male	221,308,140	76X
	295	Male	245,199,642	85X
	296	Male	214,802,737	74X
P. reticulata	FR26	Female	130,410,340	26X
	P433	Female	147,436,932	30X
	P481	Female	153,784,466	31X
	P271	Male	173,020,372	35X
	P363	Male	137,775,650	28X
	P409	Male	126,610,970	25X
P. parae	P16	Female	755,768,880	154X
	P25	Female	861,217,302	176X
	P35	Female	813,848,774	166X
	P38 (Red Melanzona)	Male	789,618,098	161X
	P07 (Blue Melanzona)	Male	771,876,214	158X
	P18 (Blue Melanzona)	Male	813,513,894	166X
